# Vascular Reconstruction in Extremity Soft Tissue Sarcomas: A Systematic Review and Single‐Arm Meta‐Analysis

**DOI:** 10.1002/jso.70194

**Published:** 2026-01-08

**Authors:** Lucas Monteiro Delgado, Bernardo Fontel Pompeu, Vinícius dos Santos Macedo, Gabriel Henrique Acedo Martins, Eric Pasqualotto, Matheus Reginato Araujo, Julia Hoici Brunini, Victor Andrade Nunes, Cláudia Theis, Samuel Aguiar Junior

**Affiliations:** ^1^ Universidade Federal de Minas Gerais (UFMG), Belo Horizonte‐MG Belo Horizonte Brazil; ^2^ Surgery Department Hospital Heliópolis São Paulo Brazil; ^3^ University of São Caetano do Sul São Caetano do Sul Brazil; ^4^ Universidade Federal de Santa Catarina (UFSC) Florianópolis Brazil; ^5^ University of North Carolina, Chapel Hill Chapel Hill North Carolina USA; ^6^ Sarcoma and Bone Tumors Reference Center ‐ A.C. Camargo Cancer Center São Paulo Brazil

**Keywords:** limb salvage, limb‐sparing surgery, meta‐analysis, soft tissue sarcoma, vascular reconstruction

## Abstract

**Introduction:**

The management of extremity soft tissue sarcomas (STS) involving major vessels presents unique challenges, historically leading to amputation. Advances in vascular reconstruction have enabled limb‐sparing surgery (LSS), but outcomes and perioperative risks remain uncertain. This systematic review and meta‐analysis aimed to evaluate oncologic results following LSS with vascular reconstruction in extremity STS.

**Methods:**

A systematic review and single‐arm meta‐analysis were performed according to PRISMA guidelines, with registration in PROSPERO. PubMed, Embase, and Cochrane Library were searched from inception to June 2025 for studies reporting outcomes in patients with extremity STS undergoing LSS with vascular reconstruction. Pooled analyses estimated limb salvage, survival, and complication rates using random‐effects models.

**Results:**

Thirty‐one studies comprising 520 patients were included. Approximately 58% were male, with a mean age ranging from 29.3 to 59 years. The most common tumor localizations were the thigh (59.5%), inguinal region (15.9%), and popliteal fossa (8.6%). Liposarcoma (24.0%), synovial sarcoma (19.6%), and osteosarcoma (14.8%) were the most frequent histological subtypes. The pooled limb salvage rate was 89% (95% CI, 86%–92%), while amputation occurred in 10% (95% CI, 8%–14%). One‐ and 5‐year overall survival rates were 89% and 62%, respectively, with disease‐free survival rates of 74% and 55%. Major complications included graft thrombosis (19%), wound complications (29%), and wound infection (22%).

**Conclusions:**

Limb‐sparing surgery with vascular reconstruction is effective for extremity STS involving major vessels, enabling high limb salvage and favorable long‐term survival without compromising oncologic outcomes. However, substantial perioperative morbidity persists, underscoring the need for multidisciplinary care, careful patient selection, and prospective studies to refine indications and enhance quality of life.

**Abbreviations**
CI: confidence interval
DFS: Disease‐Free Survival
FA: Femoral Artery
GSV: great saphenous vein
LSS: Limb‐Sparing Surgery
OS: overall survival
PRISMA: Preferred Reporting Items for Systematic Reviews and Meta‐Analyses
PROSPERO: International Prospective Register of Systematic Reviews
ROBINS‐I V2: Risk of Bias In Non‐randomized Studies of Interventions, Version 2
STS: soft tissue sarcoma

## 1 Introduction

Soft tissue sarcomas (STS) of the extremities are a heterogeneous group of malignant mesenchymal tumors that often pose a therapeutic challenge when adjacent to or infiltrating major vascular structures. Since 1989, limb‐sparing surgery (LSS) has been favored over amputation, as studies have shown that achieving negative margins (R0) is the key determinant of outcomes. Amputation is reserved for cases where R0 resection or satisfactory function cannot be achieved [1, 2].

En bloc resection of tumors with vascular involvement followed by immediate vascular reconstruction enables limb preservation without compromising oncologic outcomes, provided that negative margins are achieved [3]. Autologous vein grafts, especially the great saphenous vein (GSV), remain the conduit of choice due to superior patency and lower infection rates compared to prosthetic alternatives [4, 5, 6]. Nevertheless, vascular reconstruction in this setting is technically demanding and associated with significant morbidity, including graft thrombosis, deep wound infections, and local recurrence. Furthermore, the need for venous reconstruction remains controversial, with some authors advocating selective reconstruction based on venous caliber and intraoperative findings [7, 8].

Recent institutional experiences have underscored the critical role of a multidisciplinary approach, integrating orthopedic, vascular, and plastic surgery teams, in reducing complications and optimizing both functional and oncologic outcomes [9]. However, most available evidence is limited to small retrospective cohorts, and there is no consensus on optimal reconstruction techniques, graft types, or perioperative management. Therefore, we conducted a systematic review and single‐arm meta‐analysis to synthesize existing data on vascular reconstruction in extremity STS. Our objectives were to estimate limb salvage rates, graft patency, and perioperative complication profiles, and to provide evidence to guide clinical decision‐making in this complex surgical population.

## 2 Methods

We performed the systematic review and meta‐analysis according to the Cochrane Handbook for Systematic Reviews of Interventions and structured it according to the Preferred Reporting Items for Systematic Reviews and Meta‐Analysis (PRISMA) guidelines, presented in Supplementary Table S1 [10, 11]. The study protocol was registered in the International Prospective Register of Systematic Reviews (PROSPERO) under registration number CRD420251082601 [12].

### 2.1 Outcomes and Subgroup Analysis

The outcomes of interest were: (1) overall survival (OS); (2) disease‐free survival (DFS); (3) local recurrence; (4) distant metastasis; (5) limb salvage; (6) amputation; (7) early (≤ 6 months) and late (> 6 months) graft thrombosis; (8) graft patency; (9) reperfusion injury; (10) compartment syndrome; (11) any wound‐related complication (including dehiscence, infection, necrosis, delayed healing, seroma, and hematoma); (12) wound infection; and (13) mortality due to disease at any follow‐up time point.

We conducted subgroup analyses to explore potential sources of heterogeneity, stratifying studies by follow‐up duration (short‐term < 24 months, mid‐term 24–60 months, and long‐term > 60 months), graft type (arterial autologous, arterial prosthetic, venous autologous, venous prosthetic, arterial and venous autologous, arterial autologous with venous prosthetic, arterial prosthetic with venous autologous, and arterial and venous prosthetic grafts), and reconstruction type (arterial‐only vs combined arterial and venous reconstruction) for limb salvage. For the graft‐type subgroup analysis, studies in which no interposition graft was used (primary anastomosis) and those using allografts were excluded to preserve consistent classification of conduit material. We additionally performed a cause‐specific subgroup analysis for amputation outcomes, categorizing events according to the reported indication (vascular complications, local tumor recurrence, or postoperative infection) when such information was available and at least one event was observed.

### 2.2 Eligibility Criteria

Inclusion in this meta‐analysis was limited to studies that met all of the following eligibility criteria: (1) enrolled patients with resectable STS or osteosarcoma of the extremities; (2) included patients undergoing LSS with planned vascular reconstruction; and (3) reported at least one of the outcomes of interest.

Studies were excluded if they met any of the following criteria: (1) included retroperitoneal or trunk sarcomas without stratified outcome reporting; (2) involved patients undergoing amputation; (3) lacked full‐text availability; or (4) were case reports, trial registrations without available results, meta‐analyses, reviews, or animal studies.

### 2.3 Search Strategy and Study Selection

We systematically searched PubMed, Embase, and Cochrane Library databases from inception to June 20th, 2025. The search strategy was (“Soft Tissue Sarcoma” OR “Sarcoma Of The Extremities” OR “Extremity Sarcoma”) AND (“Vascular Reconstruction” OR “Reconstructive Surgical Procedures” OR “Revascularization” OR “Limb‐Sparing” OR “Vessel Reconstruction”). We also searched the references of the included studies and previous systematic reviews and meta‐analyses aiming for the inclusion of additional studies [13].

Two authors (L.M.D. and V.S.M.) independently conducted the search, imported results into Rayyan, a web‐based systematic review tool, and triaged the studies. After the exclusion of duplicates and titles/abstracts unrelated to the clinical question, the eligibility of each remaining study was assessed based on the review of the full‐text articles. Disagreements were solved by consensus.

### 2.4 Data Extraction

Two authors (V.S.M. and G.H.A.M) independently extracted data from the included studies using a standardized form. Extracted information included: (1) general study data (first author, year of publication, country, study design, and study period); (2) sample characteristics (number of patients, number and percentage of females, mean age, neoadjuvant treatment use, adjuvant treatment use, and follow‐up time in months); (3) tumor characteristics (tumor site, localization, histological type, mean tumor size in centimeters, and presentation status); and (4) vascular resection and reconstruction details (type of materials used for arterial and venous reconstruction, mean graft length, anticoagulation regimen, and the number and anatomical identity of arteries resected and reconstructed).

### 2.5 Risk of Bias and Quality Assessment

Two independent reviewers (L.M.D and V.S.M.) assessed the Risk of Bias In Non Randomized Studies of Interventions (ROBINS‐I V2) tool [14]. Disagreements were resolved through consensus. Funnel plots were not performed because they are not recommended for single‐arm meta‐analyses, as the relationship between study size and effect is unclear and these methods are unreliable for detecting publication bias in meta‐analyses of proportions [15, 16].

### 2.6 Statistical Analysis

Pooled proportions of outcomes were calculated using the inverse variance method with logit transformation. Between‐study heterogeneity was assessed using the Cochran Q test and the I² statistic, with heterogeneity considered significant when *p* < 0.10 and I² > 25%. In the presence of significant heterogeneity, leave‐one‐out sensitivity analysis was performed by sequentially excluding each study from the meta‐analysis to assess the stability of the results and ensure that findings were not driven by any single study. We regarded a *p* value < 0.10 as statistically significant for subgroup interaction, as per Cochrane guidelines. All statistical analyses were performed using R statistical software (version 4.5; R Foundation for Statistical Computing, Vienna, Austria).

## 3 Results

### 3.1 Study Selection

**Figure 1:**
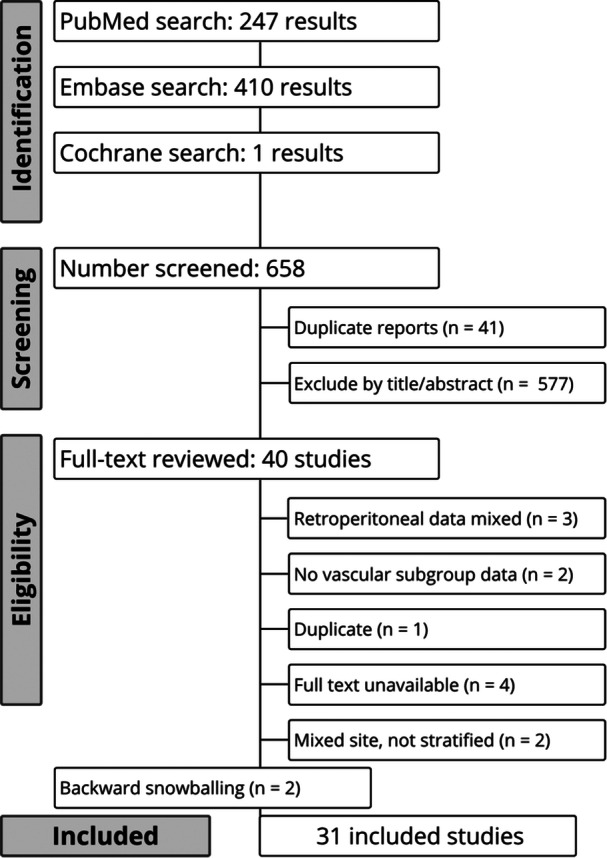
Preferred reporting items for systematic reviews and meta‐analysis (PRISMA) flow diagram of study screening and selection.

As detailed in Figure 1, the initial search identified 658 results. After removal of duplicate records and assessment of the studies based on title and abstract, 42 full‐text studies remained for full review according to prespecified criteria. Of these, 31 studies were included [2, 4, 5, 6, 7, 8, 17, 18, 19, 20, 21, 22, 23, 24, 25, 26, 27, 28, 29, 30, 31, 32, 33, 34, 35, 36, 37, 38, 39, 40, 41]. Supplementary Table S2 lists the excluded studies from the full‐text screening stage and the reasons for their exclusion.

### 3.2 Patient Characteristics

The cohort comprised 520 patients with STS of the extremities who underwent LSS with vascular reconstruction. Approximately 40.4% of the patients were female, and the mean age across studies ranged from 29.3 to 59 years. Pediatric patients were uncommon: at least 26 participants aged 6–17 years were identified among 520 total patients (5%); 14 studies reported none, and six studies did not provide sufficient data to quantify pediatric cases.

Neoadjuvant therapy was not consistently reported across studies; only 17 studies provided these data, encompassing a total of 282 patients. Among studies reporting neoadjuvant treatment, chemotherapy was the most common modality (108 patients, 38.3%), followed by radiotherapy alone (61 patients, 21.6%) and chemoradiotherapy (19 patients, 6.7%). Similarly, adjuvant therapy was inconsistently reported, with data available from 20 studies including 311 patients. Within this subset, radiotherapy was the most frequently used modality (88 patients, 28.3%), followed by chemotherapy (73 patients, 23.5%) and chemoradiotherapy (19 patients, 6.1%). General study characteristics and patient demographics are summarized in Table 1.

**Table 1:** General study characteristics and patient demographics.

First author, year	Country	Study design	Study period	No. of patients	Pediatric patients, *n* (%) [age range]	Female, *n* (%)	Mean age, years	Neoadjuvant treatment, %	Adjuvant treatment, *n* (%)	Follow‐up time, months
Adelani, 2007	United States, single‐center	Retrospective	1990–2005	14	1 (7.14) [17]	6 (42.9)	54	NA	RT: 6 (42.9) CT: 1 (7.1) RT + CT: 5 (35.7) None: 1 (7.1)	19 (12–83)^a^
Akgul, 2018	Turkey, single‐center	Retrospective	2004–2007	17	1 (5.88) [14]	8 (47.1)	37.8	NA	NA	39 (3–120)
Arikawa, 2024	Japan, single‐center	Retrospective	2010–2023	37	0	17 (45.9)	50.8	RT: 15 (40.5) CT: 17 (46.0)	NA	38 (3–161)^j^
Baxter, 2007	United States, single‐center	Retrospective	1991–2004	19	9 (47.4) [6–17]	1 (10)	45.2	CT: 12 (63.2) CT + RT: 4 (21.1) CT + intraoperative RT: 3 (15.8)	NA	68.5^b^
Bonardelli, 2000	Italy, single‐center	Retrospective	1995–1999	7	0	2 (28.6)	56.7	RT: 1 (14.3)	NA	30.9
Cetinkaya, 2019	Turkey, single‐center	Retrospective	2002–2014	13	0	7 (53.8)	47.2	CT: 3 (23.1)	RT: 6 (46.1) CT: 2 (15.4) RT + CT: 4 (30.8) None: 1 (7.2)^c^	80.6
Davis, 2017	Canada, single‐center	Retrospective	2005–2013	9	0	5 (55.6)	50.3	CT: 3 (33.3) RT: 6 (66.7)	RT: 4 (44.4) CT: 2 (22.2) None: 3 (33.3)	74.7
Emori, 2012	Japan, single‐center	Retrospective	1997–2009	10	2 (20) [12–16]	5 (62.5)	38.8	CT: 5 (62.5)	RT: 2 (25)	39^j^
Ghert, 2005	Canada, single‐center	Retrospective	1989–2000	19	0	7 (36.8)	48.3	RT: 14 (74)	CT: 1 (6) RT: 4 (24)	47
Hohenberger, 1999	Germany, multicenter	Retrospective	1989–1992	20	0	6 (30)	47^j^	NA	CT: 1 (2)	29^j^
Homsy, 2022	Finland, single‐center	Retrospective	2014–2020	8	0	2 (25)	54.6	NA	RT: 6 (75) None: 2 (25)	35.3
Kang, 2023	South Korea, multicenter	Retrospective	2005–2020	43	0	21 (k48.8)	53^j^	NA	CT: 34 (79.1) RT: 23 (53.5)ᵈ	23.8^j^
Karimi, 2025	Iran, single‐center	Prospective	2018–2020	13	0	4(30.8)	41.54	RT: 8 (61.5) CT: 5 (38.5)	CT: 2 (15.4)	20(12–24)^a^
Kawai, 1996	Japan, single‐center	Retrospective	1982–1994	8	0	6 (75)	47,5	CT + RT: 6 (80)	NA	30
Koperna, 1996	Austria, single‐center	Retrospective	1984–1992	14	4 (35) [16–17]	5 (35.7)	30	CT: 10 (71.4) RT: 1 (7.1)	CT + RT: 2 (14.3) RT: 1 (7.1)	55
Leggon, 2001	United States, single‐center	Retrospective	1969–1996	16	1 (6.25) [17]	8 (50.0)	45	NA	CT: 8 (50) RT: 4 (25)	56
Mlees, 2020	Egypt, single‐center	Prospective	2017–2019	29	k	13 (44.8)	43.5	NA	NA	12
Muramatsu, 2011	Japan, single‐center	Retrospective	1995–2010	15	1 (6.66) [17]	4	54	CT: 13 (86.7) RT:4 (26.7)	NA	69
Nishinari, 2015	Brazil, single‐center	Retrospective	1995–2010	25	k	13 (52)	38^j^	Surgery + RT + CT: 7 (28) CT: 6 (24) CT + RT: 5 (20) Surgery + CT: 3(12) Surgery + RT: 1(4)	NA	65.5
Okamoto, 2018	Japan, single‐center	Retrospective	2006–2018	8	1 (12.5) [16]	2 (25.0)	59	CT + RT: 1 (12.5)	NA	24j
Park, 2018	South Korea, single‐center	Retrospective	2009–2015	17	k	9 (52.9)	47^j^	NA	NA	23.3^j^
Ramamurthy, 2009	India, single‐center	Retrospective	2004–2008	4	0	2 (50.0)	36	NA	RT: 2 (50.0) RT + CT: 1 (25.0)	NA
Schwarzbach, 2005	Germany, single‐center	Prospective	1998–2003	21	0	11 (52.0)	56^j^	NA	RT: 13 (61.9) None: 8 (38.1)	34^j^
Shah, 2021	India, single‐center	Retrospective	1995–2019	14	k	8 (57.1)	33	NA	CT + RT: 4 (28.6) CT: 6 (42.9) RT: 1 (7.1) None: 3 (21.4)	NA
Spark, 2009	Australia, single‐center	Retrospective	2002–2006	9	k	4 (44.4)	32^j^	CT: 1 (11.1)	RT: 2 (22.2)	5‐48e
Teixeira, 2017	Brazil, single‐center	Retrospective	2002–2015	13	3 (23.1) [12–16]	4 (30.7)	36.4	CT: 7 (53.85) RT: 1 (7.6)	RT: 6 (46.1)	NA
Tsukushi, 2008	Japan, single‐center	Retrospective	1990–2004	25	0	10 (40)	46	CT: 10 (40) RT: 2 (8) IORT: 2 (8)	14 (56)^f^	70
Umezawa, 2013	Japan, single‐center	Retrospective	1996–2010	23	2 (8.7) [16–17]	7 (30.4)	44.3	CT 5 (21.7) CT + RT 3 (13.0) RT 5 (21.7)	CT: 3 (13.0) RT: 1 (4.3)	2–240^e^
Viñals, 2013	Spain, single‐center	Retrospective	2003–2010	8	0	5 (62.5)	34.9	NA	RT: 4 (50) CT: 1 (12.5) RT + CT: 3 (37.5)	34.8
Wortmann, 2017	Germany, single‐center	Retrospective	1990–2014	27	NA^i^	NAʰ	NAʰ	NAʰ	NAʰ	24.5^j^
Zheng, 2023	China, single‐center	Retrospective	2016–2021	15	1 (6.66) [15]	5 (66.6)	36	CT: 11 (73.3) RT: 1 (6.7) None: 3 (20)	CT: 12 (80) RT: 3 (20)	12.5^j^

Abbreviations: CT, chemotherapy; IORT, intraoperative radiotherapy; NA, not available; RT, radiotherapy.

^a^ Data presented as mean (range).

^b^ Data from whole cohort, considering pediatric population.

^c^ Pathological findings revealed an intramuscular lipoma, and the patient did not receive oncologic treatment.

^d^ The exact number of patients who received both or none adjuvant therapies is unknown.

^e^ Only follow‐up range was available.

^f^ Adjuvant therapy not specified.

^g^ With follow‐up presumed completed by 2024.

^h^ Data mixed with retroperitoneal sarcomas cohort.

^i^ Age was reported only in dichotomous categories; the study does not specify whether pediatric patients were included or excluded.

j Median.

^k^ Pediatric cases were reported, but insufficient data were provided to quantify the pediatric subgroup.

The most frequently reported tumor localizations were the thigh (285 patients; 59.5%), inguinal region (76 patients; 15.9%), and popliteal fossa (41 patients; 8.6%), highlighting the predominance of sarcomas in the lower extremities. The most frequently reported histological subtypes were liposarcoma (115 patients; 24.0%), synovial sarcoma (94 patients; 19.6%), and osteosarcoma (71 patients; 14.8%). The vast majority of studies reported performing en bloc resection. Among studies that contained surgical resection margin analysis, most included only patients with negative margins, although one study reported positive margins in up to 51% of patients [41]. Furthermore, minimum margin sizes were mentioned in only nine studies, ranging between 2 and 3 centimeters. Anatomopathological features and tumor profiles of included patients are summarized in Table 2.

**Table 2:** Tumor profiles and histopathological features.

First author, year	Tumor site, *n* (%)	Tumor localization, *n* (%)	Tumor type, *n* (%)	Mean tumor size, cm	Surgical margin	Presentation status, *n* (%)
Adelani, 2007	Lower extremity: 14 (100)	Thigh, 6 (42.9) Groin, 7 (50) Popliteal fossa, 1 (7.1)	Synovial sarcoma: 5 (35.7) Malignant fibrous histiocytoma: 4 (28.6) Liposarcoma: 4 (28.6) Leiomyosarcoma: 1 (7.1)	12	NA	Primary tumor: 11 (79,7) Local recurrence: 2 (14,2) Metastatic tumors: 1 (7,1)
Akgul, 2018	Upper extremity: 2 (11.8) Lower extremity: 15 (88.2)	Popliteal fossa: 6 (35.3) Distal femur: 3 (17.6) Proximal tibia: 3 (17.6) Thigh: 3 (17.6) Shoulder: 1 (5.9) Wrist: 1 (5.9)	Osteosarcoma: 4 (23.5) Ewing′s sarcoma: 3 (17.6) Chondrosarcoma: 2 (11.8) Fusiform cell sarcoma: 2 (11.8) Fibrosarcoma: 1 (5.9) Rhabdomyosarcoma: 1 (5.9) Desmoid tumor: 1 (5.9) Pleomorphic sarcoma: 1 (5.9) Giant cell tumor of tendon sheath: 1 (5.9) Synovial sarcoma: 1 (5.9)	NA	R0 (100%)	Primary tumor: 9 (56) Local recurrence: 7 (44)
Arikawa, 2024	Lower extremity: 37 (100)	Thigh 33 (89) Genicular Region 4 (11)	Myxofibrosarcoma 7 (18.9) Pleomorphic sarcoma 4 (10.8) Leiomyosarcoma 2 (5.4) Solitary fibrous tumor 2 (5.4) Myxoid liposarcoma 3 (8.1) Pleomorphic rhabdomyosarcoma 1 (2.7) Synovial Sarcoma 2 (5.4) Parosteal osteosarcoma 1 (2.7) Extraskeletal myxoid chondrosarcoma 1 (2.7) Synovial sarcoma 3 (8.1) Angiomatoid fibrous histiocytoma 2 (5.4) Liposarcoma 1 (2.7) Alveolar soft part sarcoma 1 (2.7) Extraskeletal osteosarcoma 1 (2.7) Pleomorphic spindle cell sarcoma 1 (2.7) Angiosarcoma 1 (2.7) Mesenchymal chondrosarcoma 1 (2.7) Unclassifiable sarcoma 1 (2.7)	NA	NA	NA
Baxter, 2007	Lower extremity: 19 (100)	Distal femur: 7 (36.8) Groin: 3 (15.8) Posterior compartment distal thigh: 2 (10.5) Groin and proximal medial thigh compartment: 1 (5.3) Anterior thigh compartment: 1 (5.3) Medial thigh compartment: 1 (5.3) Proximal fibula: 1 (5.3) Knee: 1 (5.3) Proximal tibia: 1 (5.3)	Osteosarcoma: 8 (42.1) Leiomyosarcoma: 2 (10.5) Angiosarcoma: 1 (5.3) Malignant fibrous histiocytoma: 1 (5.3) Fibrous histiocytoma: 1 (5.3) Myxoid liposarcoma: 1 (5.3) Extraskeletal Ewing's sarcoma: 1 (5.3) Chondrosarcoma: 1 (5.3) Osteoblastic osteosarcoma: 1 (5.3) Synovial sarcoma: 1 (5.3)	NA	NA	Primary 19 (100)
Bonardelli, 2000	Lower extremity: 7 (100)	Anterior thigh: 2 (28.6) Medial thigh: 4 (57.1) Posterior thigh: 1 (14.3)	Malignant fibrous histiocytoma: 6 (85.7) Myxoid liposarcoma: 1 (14.3)	NA	Wide surgical margin by Enneking′s criteria	Primary tumor: 5 (72) Residual: 2 (28)
Cetinkaya, 2019	Upper extremity: 1 (7.7) Lower extremity: 12 (92.3)	Forearm: 1 (7.7) Inguinal: 2 (15.4) Thigh: 8 (61.5) Popliteal: 2 (15.4)	Synovial sarcoma: 3 (23.1) Pleomorphic sarcoma: 3 (23.1) Liposarcoma: 2 (15.4) Leiomyosarcoma: 2 (15.4) Undifferentiated sarcoma: 1 (7.7) Intramuscular lipoma: 1 (7.7) Osteosarcoma: 1 (7.7)	9.1	R0 (100%)	Primary tumor: 12 (92.3) Local recurrence: 1 (7.7)
Davis, 2017	Lower extremity: 9 (100)	Distal thigh: 2 (22.2) Proximal thigh: 77.8)	Leiomyosarcoma: 3 (33.3) Epithelioid sarcoma: 1 (11.1) Malignant peripheral nerve sheath tumor: 1 (11.1) Liposarcoma: 1 (11.1) Sclerosing epithelioid fibrosarcoma: 1(11.1) Synovial sarcoma: 2 (22.2)	NA	NA	NA
Emori, 2012	Lower extremity: 10 (100)	Inguinal: 10 (100)	Synovial sarcoma: 4 (40) Leiomyosarcoma: 3 (30) Malignant fibrous histiocytoma: 1 (10) Extraskeletal mesenchymal chondrosarcoma: 1 (10) Ewing′s sarcoma: 1 (10)	7.0	R0 (100%)	Primary tumor: 6 (60) Local recurrence: 2 (20) Residual tumors: 2 (20)
Ghert, 2005	Lower extremity: 19 (100)	NA	Myxoid liposarcoma: 3 (15.8) Hemangiopericytoma: 1 (5.3) Malignant peripheral nerve sheath tumor: 2 (10.6) Malignant fibrous histiocytoma: 4 (21.2) Other: 9 (47.4)	10.9	R0 (84%); R1 (16%)	Primary tumor: 18 (94.7) Local recurrence: 1 (5.3)
Hohenberg, 1999	Lower extremity: 20 (100)	Hunter's canal: 7 (35) Groin: 4 (20) Thigh: 2 (10) Popliteal fossa: 4 (20) Small pelvis and groin: 1 (5) Quadriceps and adductor muscle: 1 (5) Flexor group lower limb: 1 (5)	Malignant fibrous histiocytoma: 6 patients (30) Leiomyosarcoma: 5 patients (25) Liposarcoma: 4 patients (20) Alveolar type: 2 patients (10) Mesenchymoma: 1 patient (5) Embryonal type: 1 patient (5) Synovial sarcoma: 1 patient (5)	NA	R0 (100%)	Primary 10 (50) Local Recurrence 5 (25) Residual 5 (25)
Homsy, 2022	Lower extremity: 8 (100)	Proximal thigh or inguinal: 8 (100)	Leiomyosarcoma: 4 (50) Myoepithelioma: 1 (12.5) Myxoid lipossarcoma: 1 (12.5) Alveolar soft tissue sarcoma: 1 (12.5) Synovial sarcoma: 1 (12.5)	NA	NA	Primary tumor: 6 (75) Local recurrence: 2 (25)
Kang, 2023	Upper extremity: 7 (16.3) Lower extremity: 36 (83.7)	NA	Osteosarcoma: 8 (18.6) Pleomorphic sarcoma: 6 (14) Liposarcoma: 6 (14) Leiomyosarcoma: 5 (11.6) Myxofibrosarcoma: 4 (9.3) Synovial sarcoma: 4 (9.3) Ewing sarcoma: 2 (4.7) Chondrosarcoma: 1 (2.3) Epithelioid sarcoma: 2 (4.7) Fibromatosis: 2 (4.7) Other: 3 (7)^a^	7.9 and 4.5^b^	R0 (69,8%); R1 (23,3%); R2 (7%)	Primary tumor: 26 (60.5) Local recurrence: 17 (39.5)
Karimi, 2025	Upper extremity: 1 (7,7) Lower extremity: 12 (92,3)	NA	Chondrosarcoma: 1 (7.7). Liposarcoma: 2 (15.4) Osteosarcoma: 4 (30.8) Synovial sarcoma: 3 (23.1) Undifferentiated Pleomorphic Sarcoma: 3 (23.1)	10.92	R0 (69,2%); R1 (30,8%)	Primary tumor: 8 (61.5) Local recurrence: 5 (38.5)
Kawai, 1996	Lower extremity: 8 (100)	Thigh: 6 (75) Popliteal fossa: 2 (25)	Synovial sarcoma: 3 patients Malignant fibrous histiocytoma: 1 patient Malignant schwannoma: 1 patient Desmoid: 1 patient Liposarcoma: 1 patient Clear cell sarcoma: 1 patient	NA	NA	NA
Koperna, 1996	Lower extremity: 15 (100)	Femur: 9 (64.3) Pelvic bone: 2 (14.3) Proximal tibia: 2 (14.3) Distal femur: 1 (7.1) Pubic bone: 1 (7.1)	Osteosarcoma: 8 (57.1) Liposarcoma: 2 (14.3) Ewing sarcoma: 1 (7.1) Malignant fibrous histiocytoma: 1 (7.1) Synovial sarcoma: 1 (7.1) Fibrosarcoma: 1 (7.1)	NA	R0 (100%)	NA
Leggon, 2001	Lower Extremity: 14 (87.5) Upper Extremity: 2 (12.5)	Thigh: 6 (40) Groin: 2 (13.33) Proximal Tibia: 1 (6.67) Pelvis: 1 (6.67) Calf: 1 (6.67) Knee: 1 (6.67) Arm: 1 (6.67) Proximal fibula and anterior tibia: 1 (6.67) Distal femur: 1 (6.67)	Liposarcoma: 3 (21.43) Malignant fibrous histiocytoma: 2 (14.29) Fibrosarcoma: 1 (7.14) Rhabdomyosarcoma: 1 (7.14) Synovial sarcoma: 1 (7.14) Spindle cell sarcoma: 1 (7.14) Osteogenic sarcoma: 3 (21.43) Dedifferentiated parosteal osteosarcoma: 1 (7.14) Chondrosarcoma: 1 (7.14)	9.5	NA	Primary tumor: 7 (43) Local recurrence: 2 (14) Residual 7 (43)
Mlees, 2020	Upper extremity: 7 (24.2) Lower extremity: 22 (75.8)	Arm: 5 (17.2) Forearm: 2 (6.8) Inguinal: 2 (6.8) Thigh: 19 (65.8) Infragenicular: 1 (3.4)	Rhabdomyosarcoma: 10 (34.4) Well‐differentiated liposarcoma: 6 (20.6) Myxoid liposarcoma: 2 (7.1) High‐grade pleomorphic sarcoma:: 11 (37.9)	12.34	R0 (100%)	Primary tumor 29 (100)
Muramatsu, 2011	Lower extremity: 14 (100)	Proximal: 7 (46.67) Distal: 4 (26.67) Middle: 2 (13.33) Groin: 2 (13.33)	Malignant fibrous histiocytoma: 5 (33.33) Synovial sarcoma: 4 (26.67) Liposarcoma: 2 (13.33) Osteosarcoma: 2 (13.33) Chondrosarcoma: 1 (6.67) Leiomyosarcoma: 1 (6.67)	16	NA	Primary tumor 14 (100)
Nishinari, 2015	Lower extremity: 25 (100)	Tight: 20 (80) Inguinal region: 4 (16) Infrageniculate region: 1 (4)	Synovial sarcoma: 8 (32) Liposarcoma: 3 (12) Fibrosarcoma: 2 (8) Pleomorphic sarcoma: 2 (8) Leiomyosarcoma: 2 (8) Fibromyxoid tumor: 1 (4) Fibrohistiocytoma: 1 (4) Myxofibrosarcoma: 1 (4) Malignant peripheral nerves sheath tumor: 1 (4) Chondrosarcoma: 1 (4) Hemangiopericytoma: 1 (4) High‐grade fusocellular sarcoma: 1 (4) Ewing Sarcoma: 1 (4)	NA	R0 (72%); R1 (28%)	NA
Park, 2018	Upper extremity: 2 (11.9) Lower extremity: 15 (88.2)	Tight: 12 (70.6) Calf: 2 (11.7) Inguinal: 1 (5.9) Axilla: 1 (5.9) Upper arm: 1 (5.9)	Osteosarcoma: 3 (17.7) Synovial sarcoma: 2 (11.7) Chondrosarcoma: 2 (11.7) Fibromatosis: 2 (11.7) Ewing sarcoma: 1 (5.9) Pleomorphic spindle cell sarcoma: 1 (5.9) Angiosarcoma: 1 (5.9) Myxoid malignant fibrous histocytoma: 1 (5.9) Undifferentiated sarcoma: 1 (5.9) Malignant hemangiopericytoma: 1 (5.9) Fibrohistiocytoma: 1 (5.9) Hemangioendoendothelioma: 1 (5.9)	NA	NA	Primary tumor: 11 (64.7) Local recurrence: 6 (35.3)
Ramamurthy, 2009	Lower extremity: 4 (100)	Tight: 4 (100)	Synovial Sarcoma: 1 (25.0) Rhabdomyosarcoma: 1 (25.0) Malignant peripheral nerve sheath tumor: 1 (25.0) MFH: 1 (25.0)	13.5	NA	NA
Schwarzbach, 2005	Lower extremity: 21 (100)	Tight: 16 (76.0) Groin: 5 (24.0)	Liposarcoma: 11 (52.0) Leiomyosarcoma: 4 (19.0) Malignant fibrous histiocytoma: 3 (14.0) Synovial sarcoma: 2 (10.0) Angiosarcoma: 1 (6.0)	NA	R0 (100%)	Primary tumor: 15 (71.0) Local recurrence: 6 (29.0)
Shah, 2021	Upper extremity: 2 (16.6) Lower extremity: 12 (83.4)	Tight: 11 (78.5) Leg: 1 (7.1) Arm: 2 (14.4)	Osteosarcoma 5 (35.7) Synovial sarcoma 4 (28.6) Fibromatosis: 2 (14.3) Liposarcoma: 1 (7.1) Malignant peripheral nerve sheath tumor: 1 (7.1) Giant cell tumor: 1 (7.1)	NA	R0 (74%) R1 (26%)	NA
Spark, 2009	Lower extremity: 8 (100)	Popliteal fossa: 4 (50) Groin or medial thigh: 4 (50)	Leiomyosarcoma: 4 (57) Synovial chondrosarcoma: 1 (14) Synovial sarcoma: 1 (14) Liposarcoma: 1 (14) Malignant peripheral nerve sheath tumour: 1 (14)	NA	R0 (100%)	Primary tumor: 7 (87.5) Local recurrence: 1 (12.5)
Teixeira, 2017	Upper extremity: 1 (7.7) Lower extremity: 12 (92.3)	Popliteal: 3 (23.5) Femur: 2 (15.3) Tibia: 4 (30.7) Thigh: 1 (7.6) Arm: 1 (7.6) Inguinal: 2 (15.3)	Malignant fibrous histiocytoma: 4 (30.7) Osteosarcoma: 3 (20.3) Fibrosarcoma: 1 (7) Ewing's sarcoma: 1 (7) Synovial sarcoma: 1 (7) Pleomorphic sarcoma: 1 (7) Chondrosarcoma: 1 (7) Hemangiopericytoma: 1 (7) Hemangiopericytoma: 1 (7)	NA	NA	NA
Tsukushi, 2008	Lower extremity: 25 (100)	Groin 6 (24) Thigh 17 (68) Popliteal 2 (8)	Liposarcoma 9 (36) Synovial sarcoma 5 (20) Malignant fibrous histiocytoma 3 (12) Leiomyosarcoma 2 (8) Rhabdomyosarcoma 2 (8) Epithelioid sarcoma 1 (4) Extraskeletal myxoid chondrosarcoma 1 (4) Extraskeletal osteosarcoma 1 (4) Malignant peripheral nerve sheath tumor 1 (4)	NA	NA	NA
Umezawa, 2013	Lower extremity: 23 (100)	Thigh: 15 (65.2) Groin: 5 (21.7) Popliteal: 2 (8.7) Lower leg: 1 (4.2)	Liposarcoma: 9 (39.1) Osteosarcoma: 4 (17.4) Myxofibrosarcoma: 3 (13.0) Synovial sarcoma: 3 (13.0) Chondrosarcoma: 2 (8.7) Angiomatoid fibrous histiocytoma: 2 (8.7)	NA	R0 (100%)	Primary tumor: 21 (100)
Viñals, 2013	Upper extremity: 2 (25) Lower extremity: 6 (75)	Thigh: 6 (75) Axilla: 1 (12.5) Forearm: 1 (12.5)	Leiomyosarcoma: 1 (12.5) Synovial sarcoma: 5 (62.5) Malignant peripheral nerves sheath tumor: 1 (12.5) Extraskeletal osteosarcoma: 1 (12.5)	NA	R0 (98%) R1 (2%)	Primary tumor: 2 (25) Local recurrence: 6 (75)
Wortmann, 2017	Lower extremity: 27 (100)	NA^d^	NA^d^	10.5	R0 (88%); R1 (12%)	Primary tumor: 18 (66.6) Local recurrence: 9 (33.3)
Zheng, 2023	Lower extremity: 15 (100)	Groin: 4 (26.7) Thigh: 7 (46.7) Popliteal: 3 (20) Lower leg: 1 (6.7)	Liposarcoma: 3 (20.0) Synovial sarcoma: 3 (20.0) Undifferentiated pleomorphic sarcoma: 3 (20.0) Leiomyosarcoma: 1 (6.7) Osteosarcoma: 5 (33.3)	≤ 10 cm: 5 (33.3) > 10 cm: 10 (66.7)^c^	R0 (49%); R1 (32%); R2 (6%); Rx (13%)	Primary tumor: 11 (73.3) Local recurrence: 4 (26.7)
Okamoto 2018	Lower extremity: 8 (100)	Thigh: 6 (75) Groin: 2 (25)	Liposarcoma: 3 (37) Myxofibrosarcoma: 2 (25) Synovial sarcoma: 1 (12.5) Leiomyosarcoma: 1 (12.5)	9	R0 (100%)	Primary 8 (100)

Abbreviation: NA, not available.

^a^ Other histologies: angiosarcoma, undifferentiated sarcoma, and myxoid malignant fibrous histiocytoma.

^b^ Anatomic site: data reported separately for lower and upper extremities, respectively.

^c^ Tumor size reporting: tumor size data were dichotomized.

^d^ Mixed cohort: data were reported together with a retroperitoneal sarcoma cohort.

Autologous grafts were predominantly used for both arterial (347 patients, 72.1%) and venous (214 patients, 64.8%) reconstructions. Average graft lengths ranged from 12.5 to 25.7 centimeters. While many authors did not report postoperative anticoagulation regimens, heparin and warfarin were the most commonly used agents among those who did. Further details on vascular reconstruction procedures and postoperative anticoagulation are presented in Table 3.

**Table 3:** Vascular involvement, resection, and reconstruction techniques.

First author, year	Arterial reconstruction, *n* (%)				Venous reconstruction, *n* (%)				Average graft length, cm	Anticoagulation therapy	Reconstructed artery by location
	Autologous graft	Synthetic graft	Primary anastomosis	Other	Autologous graft	Synthetic graft	Primary anastomosis	Other			
Adelani, 2007	7 (50)	7 (50)	0	0	6 (100)	0	0	0	16.9	Warfarin or aspirin	EIA—SFA DFA EIA—CFA EIA—PopA CFA—SFA SFA SFA—PopA DFA PopA
Akgul, 2018	15 (88.2)	0	2 (11.8)	0	0	2 (11.8)	0	0	NA	Warfarin or aspirin	FA PopA PTA AX RAD UL
Arikawa, 2024	37 (100)	0	0	0	10 (27.0)	0	0	0	14.2	None	NA
Baxter, 2007	9 (47.4)	0	10 (52.6)	0	9 (47.4)	2 (10.5)^a^	10 (52.6)	0	NA	NA	CFA SFA PopA
Bonardelli, 2000	2 (28.6)	2 (28.6)	0	2 (28.6)^b^	5 (71.4)	0	0	2 (28.6)^c^	NA	Dicumarolic drugs^c^	NA
Cetinkaya, 2019	11 (84.6)	1 (7.7)	0	0	12 (92.3)	0	0	0	25.7	Heparin and warfarin	BRA FA PopA
Davis, 2017	8 (88.9)	0	1 (11.1)	**0**	0	0	1 (11.1)	0	NA	NA	EIA—SFA CFA—PopA FA—PopA SFA—SFA SFA—PopA
Emori, 2012	0	5 (50)	0	5 (50)^d^	0	9 (90.0)	0	0	13.6	Cilostazol, heparin, and warfarin	EIA—SFA + DFA EIA—SFA CFA—SFA CFA—SFA + DFA
Ghert, 2005	12 (63.2)	4 (21.1)	2 (10.5)	0	11 (57.9)	0	1 (5.3)	1	NA	Heparin and warfarin	EIA—FA FA PopA PTA
Hohenberger, 1999	10 (52.6)	9 (47.4)	0	0	6 (54.5)	4 (36.4)	1 (9.1)	0	NA	NA	SFA PopA PopA—ATA FA FA—PopA
Homsy, 2022	6 (37.5)	0	0	10 (62.5)^e^	6 (37.5)	0	0	10 (62.5)ᵉ	NA	Aspirin, clopidogrel, and heparin	EIA—SFA SFA SFA DFA
Kang, 2023	28 (65.1)	10 (23.3)	0	5 (11.6)^f^	12 (57.1)	5 (23.8)	0	4 (19.0)^f^	NA	NA	EIA—FA EIA—PopA FA FA—PopA PopA PopA—PTA AX BRA RAD
Karimi, 2025	3 (23.1)	0	0	0	0	0	0	0	NA	NA	NA
Kawai, 1996	4 (50)	4 (50)	0	0	2 (28.6)	5 (71.4)	0	0	12.5	Warfarin	PopA 2 SFA 6
Koperna, 1996	9 (60.0)	3 (20.0)	0	0	6 (40.0)	2 (13.3)	0	0	NA	Heparin, antiplatelet therapy, and oral anticoagulants^h^	PopA FA—PopA FA
Leggon, 2001	13 (81.3)	1 (6.3)	2 (12.5)	0	10 (71.4)	0	2 (14.3)	0	14,00	NA	FA PopA EIA—FA PTA PTA/ATA
Mlees, 2020	16 (55.2)	13 (44.8)	0	0	4 (57.1)	3 (42.9)	0	0	12 (autologous)/15 (synthetic)	Novel oral anticoagulants and antiplatelet therapy^h^	EIA—FA FA FA—PopA Pop A—PTA AX—BRA BRA
Muramatsu, 2011	12 (85.7)	2 (14.3)	0	0	10 (83.3)	2 (16.7)	0	0	21,00	Warfarin and panalzine	FA
Nishinari, 2015	17 (81.0)	4 (19.0)	0	0	18 (78.3)	5 (21.7)	0	0	NA	NA	EIA—SFA EIA—DFA PopA CFA—SFA FA—PopA SFA—SFA DFA—PopA Pop A—PTA
Okamoto 2018	6 (85.7)	1 (14.3)	0	0	8 (100)	0	0	0	NA	NA	EIA—SFA DFA 1 CFA—SFA DFA 4 SFA 2
Park, 2018	11 (78.6)	3 (21.4)	0	0	10 (77.0)	3 (23.0)	0	0	NA	Heparin plus others (according to surgeon′s preference)^h^	FA PopA PopA—PTA AX—BRA BRA
Ramamurthy, 2009	3 (100)	0	0	0	2 (100)	0	0	0	NA	Heparin and warfarin	SFA
Schwarzbach, 2005	8 (40.0)	12 (60)	0	0	2 (16.7)	10 (83.3)	0	0	NA	Heparin and phenprocoumon	EIA—FA EIA—CRU FA—FA FA—PopA DFA
Shah, 2021	7 (50.0)	7 (50.0)	0	0	5 (50.0)	5 (50.0)	0	0	NA	Heparin, aspirin, and acenocoumarol	FA 7 PopA 4 BRA 2 PTA 1
Spark, 2009	8 (100)	0	0	0	8 (100)	0	0	0	NA	Clexane for 48 h, then standard DVT prophylaxis	CFA SFA SFA—PopA PopA
Teixeira, 2017	12 (92.3)	1 (7.7)	0	0	NA	NA	NA	NA	NA	NA	EIA—FA FA—PopA PopA BRA
Tsukushi, 2008	20 (80.0)	5 (20.0)	0	0	7 (58.3)	5 (41.7)	0	0	12.8	Warfarin	NA
Umezawa, 2013	22 (95.7)	1 (4.3)	0	0	13 (100)	0	0	0	13	Heparin (given to one patient only)	EIA FA PopA ATA
Viñals, 2013	8 (100)	0	0	0	1 (100)	0	0	0	NA	NA	EIA—FA SFA CUB AX
Wortmann, 2017	23 (49)	24 (51)	0	1^g^	2 (18)	9 (82)	0	0	NA	NA	EIA—FA FA SFA FA—PopA PopA PopA—CRU
Zheng, 2023	0	15 (100)	0	0	0	3 (100)	0	0	NA	Enoxaparin until oral anticoagulation was feasible^h^, plus aspirin	FA SFA PopA PTA

Abbreviations: ATA, anterior tibial artery; AX, axillary artery; BRA, brachial artery; CFA, common femoral artery; CIA, common iliac artery; CRU, crural arteries; CUB, cubital artery; DFA, deep femoral artery; EIA, external iliac artery; FA, femoral artery; NA, not applicable; PopA, popliteal artery; PTA, posterior tibial artery; RAD, radial artery; SFA, superficial femoral artery; UL, ulnar artery.

^a^ Synthetic venous grafts (PTFE) were used only as salvage in 2 adult patients following early graft failure.

^b^ Other (arterial) = subadventitial dissection without arterial replacement (artery preserved; no graft).

^c^ “Other (venous): distal saphenous vein transposition onto the femoral vein (veno‐venous transposition); one case used external rigid support at the anastomosis.

^d^ Combined reconstruction: autologous and synthetic grafts used in combination.

^e^ Other: cryopreserved vascular allografts.

^f^ Other: cryopreserved vascular allografts (cadaveric femoral artery/vein).

^g^ Other: one extra‐anatomic axillo‐bifemoral bypass.

^h^ Drug protocol: authors provided no further clarification.

### 3.3 Pooled Analyses

#### 3.3.1 Limb Salvage and Amputation

**Figure 2:**
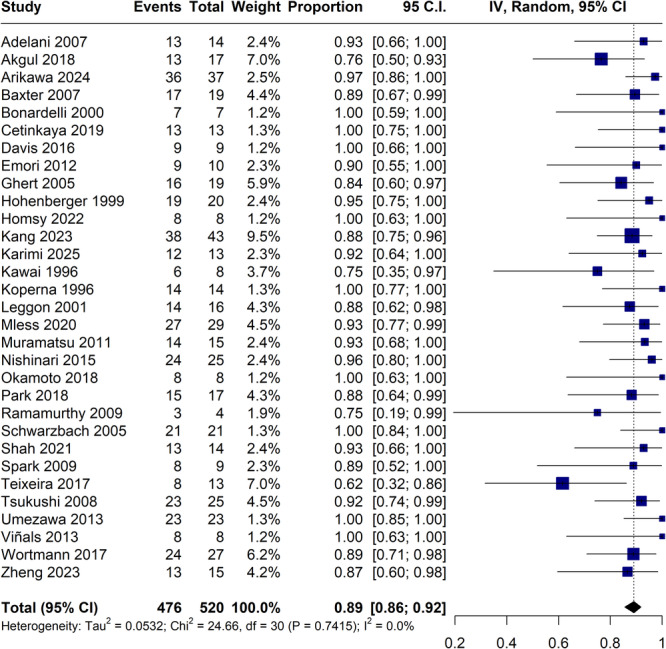
Forest plot for limb salvage. CI, confidence interval; IV, inverse variance.

**Figure 3:**
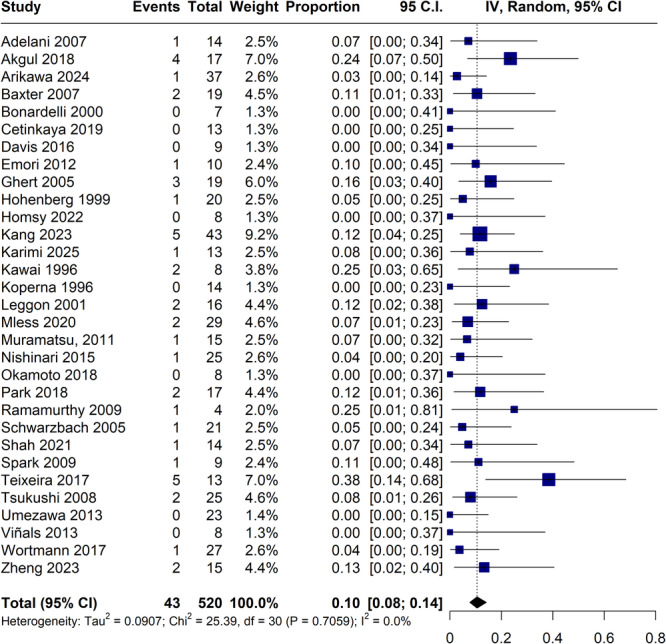
Forest plot for amputation. CI, confidence interval; IV, inverse variance.

The limb salvage rate was 89% (95% CI, 86%–92%; I² = 0%; Figure 2), while the amputation rate was 10% (95% CI, 8%–14%; I² = 0%; Figure 3). Amputations, although infrequent, occurred in diverse clinical contexts across the 31 studies. The main causes were vascular complications such as graft thrombosis or occlusion, as well as local tumor recurrence and postoperative infections. Timing varied from immediate postoperative loss to delayed amputations occurring several months after surgery. While many studies reported no amputations, others lacked detailed reporting on causality or timing. Details are summarized in Table 4.

**Table 4:** Amputation Details Across Included Studies.

Study	Total patients	Amputation events	Cause(s) of amputation	Time interval to amputation
Adelani, 2007	14	1	Acute arterial graft occlusion	Not reported
Akgul, 2018	17	4	Two due to thrombosis and wound infection; two due to local recurrence	Perioperative period (wound infection cases)
Arikawa, 2024	37	1	Graft occlusion	Postoperative day 1
Baxter, 2007	19	2	One due to unrecognized anterior compartment syndrome; one due to arterial thrombosis	Eight months postoperatively (thrombosis case)
Bonardelli, 2000	7	0	NA	NA
Cetinkaya, 2019	13	0	NA	NA
Davis, 2016	9	0	NA	NA
Emori, 2012	10	1	Graft occlusion	Not reported
Ghert, 2005	19	3	One due to wound infection; one due to rupture of iliofemoral repair; one due to vascular anastomosis breakdown	Not reported
Hohenberg, 1999	20	1	Graft failure	Postoperative day 17
Homsy, 2022	8	0	—	—
Kang, 2023	43	5	Not reported	Not reported
Karimi, 2025	13	1	Graft failure	Not reported
Kawai, 1996	8	2	One due to graft infection; one due to acute occlusion of the infrapopliteal arteries	Not reported
Koperna, 1996	14	0	NA	NA
Leggon, 2001	16	2	One due to local recurrence; one due to acute vascular occlusion	Not reported
Mless, 2020	29	2	Local recurrence	Eight and 20 months postoperatively, respectively
Muramatsu, 2011	15	1	Gradual deterioration with ischemic pain	Seven months postoperatively
Nishinari, 2015	25	1	Local recurrence	Eight months postoperatively
Okamoto, 2018	8	0	NA	NA
Park, 2018	17	2	Local recurrence	Not reported
Ramamurthy, 2009	4	1	Limb gangrene	Not reported
Schwarzbach, 2005	21	1	Local recurrence	Twelve months postoperatively
Shah, 2021	14	1	Full‐thickness muscle necrosis	Not reported
Spark, 2009	9	1	Local recurrence	Ten months postoperatively
Teixeira, 2017	13	5	Not reported	Not reported
Tsukushi, 2008	25	2	Postoperative complications	Not reported
Umezawa, 2013	23	0	NA	NA
Viñals, 2013	8	0	NA	NA
Wortmann, 2017	27	1	Life‐threatening bleeding events	Not reported
Zheng, 2023	15	2	Local recurrence	Not reported

#### 3.3.2 Survival and Oncologic Outcomes

The pooled proportion of OS was 89% at 1 year (95% CI, 82%–94%; I² = 56.5%; Supplementary Figure 1), 78% at 2 years (95% CI, 71%–83%; I² = 29.6%; Supplementary Figure 2), 71% at 3 years (95% CI, 58%–81%; I² = 65.4%; Supplementary Figure 3), and 62% at 5 years (95% CI, 51%–72%; I² = 61.8%; Supplementary Figure 4).

The pooled proportion of DFS was 74% at 1 year (95% CI, 60%–84%; I² = 58.3%; Supplementary Figure 5), 56% at 2 years (95% CI, 44%–68%; I² = 45.3%; Supplementary Figure 6), 42% at 3 years (95% CI, 25%–62%; I² = 65.4%; Supplementary Figure 7), and 55% at 5 years (95% CI, 34%–74%; I² = 79.7%; Supplementary Figure 8).

The pooled proportion of mortality related to the disease at any follow‐up was 35% (95% CI, 30%–43%; I² = 21.0%; Supplementary Figure 9); local recurrence, 14% (95% CI, 10%–19%; I² = 10.1%; Supplementary Figure 10); and distant metastasis, 31% (95% CI, 25%–37%; I² = 21.9%; Supplementary Figure 11).

#### 3.3.3 Graft‐Related Outcomes

Overall graft thrombosis rate was 19% (95% CI, 13%–26%; I² = 56.5%; Supplementary Figure 12 **)**; early graft thrombosis, 14% (95% CI, 9%–21%; I² = 408%; Supplementary Figure 13 **)**, and late graft thrombosis, 7% (95% CI, 4%–14%; I² = 41.4%; Supplementary Figure 14 **).**

The pooled proportion of graft patency was 81% at 1 year (95% CI, 74%–87%; I² = 21.1%; Supplementary Figure 15), 72% at 2 years (95% CI, 66%–77%; I² = 1.1%; Supplementary Figure 16), 72% at 3 years (95% CI, 65%–79%; I² = 26.2%; Supplementary Figure 17), and 69% at 5 years (95% CI, 58%–78%; I² = 52.7%; Supplementary Figure 18). We observed only one reperfusion injury and four compartment syndrome events in the entire cohort.

#### 3.3.4 Wound‐Related Outcomes

Wound complications rate was 29% (95% CI, 19%–40%; I² = 66.6%; Supplementary Figure 19); and wound infection was 22% (95% CI, 15%–30%; I² = 37.5%; Supplementary Figure 20).

### 3.4 Sensitivity Analysis

For 1‐year OS, the exclusion of the study by Ghert et al. eliminated heterogeneity and increased the pooled proportion to 90%. The removal of the study by Kang et al. significantly reduced heterogeneity for DSF at years 1, 2, and 3, resulting in a corresponding increase in the pooled proportions at each time point. For early and late thrombosis events, the exclusion of the studies by Shah et al. and Homsy et al., respectively, eliminated heterogeneity. For the remaining outcomes, the exclusion of any single study did not significantly alter heterogeneity. The sensitivity analyses are detailed in Supplementary Figures S21‐S36.

### 3.5 Subgroup Analyses

Supplementary Figure S37 shows amputation rates by cause, including 16/186 amputations due to vascular complications, 4/34 due to postoperative infection, and 12/129 due to local recurrence, with no significant differences between subgroups (*χ*² = 0.11, *p* = 0.95). Supplementary Figure S38 shows limb salvage rates by follow‐up duration, with pooled limb salvage of 0.90 in short‐term (118/131), 0.89 in mid‐term (203/220), and 0.93 in long‐term studies (100/106), with no significant differences between subgroups (*χ*² = 1.35, *p* = 0.509).

Supplementary Figure S39 shows limb salvage rates stratified by graft type. Most patients underwent reconstruction with autologous grafts, which showed consistently high limb salvage rates. Prosthetic and mixed graft subgroups were small and demonstrated wider confidence intervals. No statistically significant differences were observed between graft‐type subgroups (*χ*² = 5.54, *p* = 0.698). This subgroup analysis differed slightly from the primary analysis because the logit transformation did not converge due to the number of subgroups and the limited number of studies within each subgroup; therefore, we used the Freeman–Tukey double arcsine transformation for this analysis. Supplementary Figure S40 shows limb salvage rates stratified by reconstruction type. Combined arterial and venous reconstruction demonstrated numerically higher limb salvage rates compared with arterial‐only and venous‐only reconstruction; however, subgroup differences were not statistically significant (*χ*² = 1.59, *p* = 0.45).

### 3.6 Risk of Bias Assessment

**Figure 4:**
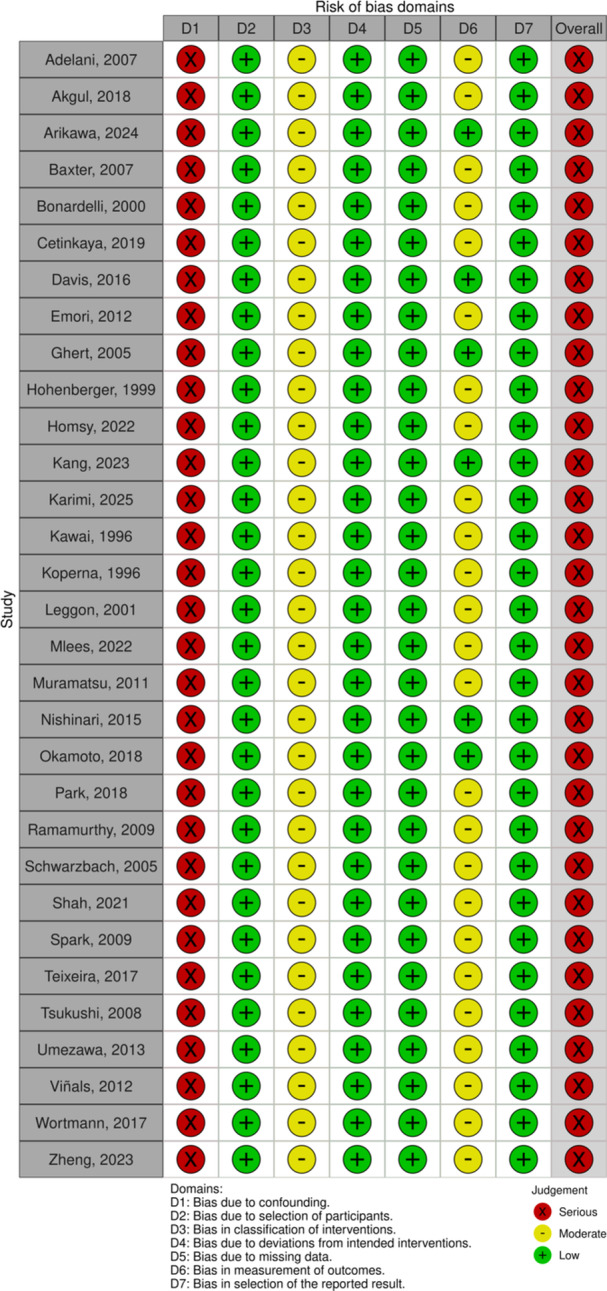
Risk of bias assessment.

All included non‐randomized studies were assessed using the ROBINS‐I V2 tool. Across the cohort, the overall risk of bias was considered serious, primarily due to the presence of uncontrolled confounding. Other domains, such as classification of interventions, deviations from intended interventions, and missing data, were consistently rated as low risk. Most studies exhibited moderate risk for selection of participants and measurement of outcomes, reflecting retrospective designs and subjective outcome assessments. The risk of bias assessment is detailed in Figure 4.

## 4 Discussion

In this systematic review and single‐arm meta‐analysis of 31 studies including 520 patients with extremity soft tissue sarcomas undergoing limb‐sparing surgery with vascular reconstruction, we found a limb salvage rate of 89% and an amputation rate of 11%. Pooled overall survival was 89% at 1 year, 78% at 2 years, 70% at 3 years, and 60% at 5 years, while disease‐free survival was 74%, 56%, 42%, and 55% at the same time points, respectively. Disease‐related mortality occurred in 36% of patients, with local recurrence in 14% and distant metastasis in 31%. The overall graft thrombosis rate was 19%, with early and late thrombosis rates of 14% and 8%. Graft patency was 81% at 1 year, 72% at 2 and 3 years, and 69% at 5 years. Wound complications and wound infections occurred in 29% and 22% of patients, respectively.

The high limb salvage rate observed in our study is consistent with rates reported in previous series of limb‐sparing resections [42]. This suggests that the necessity for vascular reconstruction does not substantially compromise the ability to preserve the limb when compared to less complex resections. The observed amputation rate of 11% in this meta‐analysis, although relatively low, underscores the ongoing challenges of limb‐sparing surgery with vascular reconstruction in patients with extremity soft tissue sarcomas. Amputations were primarily related to vascular complications, local tumor recurrence, and postoperative infections. Achieving radical excision is often difficult due to anatomical constraints, tumor size, and proximity to major neurovascular structures, which can increase the risk of inadequate margins and local recurrence, ultimately leading to limb loss. Notably, soft tissue sarcomas have a recognized propensity for local recurrence when margins are insufficient, further complicating limb preservation efforts [43].

Overall survival declined from 89% at 1 year to 60% at 5 years, while disease‐free survival decreased from 74% to 55%. This substantial decrease over time underscores the continued impact of early recurrence and distant relapse on long‐term outcomes in extremity soft tissue sarcomas, as also demonstrated by a large, prospective, multicenter study, which demonstrated that recurrence and metastatic spread are the main factors limiting long‐term survival in patients with extremity soft tissue sarcomas [43].

The significant rates of local recurrence (14%) and distant metastasis (31%) in our cohort underscore the aggressive behavior of extremity soft tissue sarcomas and the persistent challenge of achieving durable oncologic control. While recent studies have suggested that routine imaging may have limited utility in detecting local recurrence, chest imaging remains essential for the identification of pulmonary metastases, especially in high‐grade sarcomas. This approach aligns with evidence supporting the role of lung surveillance as a critical component in managing these patients [44].

Although most vascular reconstructions remain patent in the first year after surgery, there is a gradual decline in patency over time, largely attributable to both early and late thrombotic events. Vigilant postoperative monitoring and long‐term antithrombotic strategies can minimize graft failure and its associated complications. We observed only one reperfusion injury and four compartment syndrome events in the entire cohort, indicating that these serious complications were rare. Additionally, wound complications occurred in 29% of patients, with wound infections affecting 22% of the patients. Notably, neoadjuvant radiotherapy is a recognized risk factor for wound infection, as preoperative radiation can impair tissue healing [45]. A considerable proportion of patients in our sample underwent this treatment modality, which may have contributed to the observed rates of wound morbidity.

This study has several major limitations. First, the included studies span a wide time period, during which significant advances in surgical technique, perioperative management, and oncologic therapies likely occurred, introducing substantial temporal and methodological heterogeneity. There was marked inconsistency in the use and reporting of adjuvant and neoadjuvant treatments, with little to no information on specific drugs, regimens, or dosages, making it impossible to analyze the impact of these therapies. Critically, data on surgical margin status were frequently absent or incompletely reported, precluding any meaningful pooled analysis or robust assessment of the fundamental oncologic principle of margin negativity, which remains one of the strongest predictors of local recurrence and long‐term survival in sarcoma care.

Follow‐up duration was highly variable both within and between studies, further complicating interpretation of survival and recurrence outcomes. Several pooled outcomes showed substantial heterogeneity despite the use of random‐effects models and sensitivity and subgroup analyses. This residual variability likely reflects intrinsic clinical and methodological differences among studies. Therefore, pooled estimates should be interpreted as indicative of a range of effects, and conclusions should focus on overall consistency and feasibility rather than precise effect sizes. Importantly, high I² values reduce confidence in the precision of individual pooled estimates and indicate that true effects may differ substantially across clinical settings. Most included studies were retrospective, single‐center series subject to selection and reporting biases. The lack of standardized definitions for complications, recurrence, and other key outcomes also contributed to inconsistent data capture. Finally, the risk of publication bias remains high in this literature, and conventional tools for assessing it are unreliable in single‐arm meta‐analyses. Together, these limitations significantly restrict the strength and generalizability of our conclusions, highlighting the urgent need for high‐quality, prospective multicenter research with comprehensive and standardized data collection.

## 5 Conclusion

In conclusion, limb‐sparing surgery with vascular reconstruction appears to be a feasible and reasonable option for selected patients with extremity soft tissue sarcomas involving major vessels. This strategy may achieve high limb salvage and encouraging long‐term survival rates, while maintaining oncologic rigor. Although perioperative morbidity remains a concern, outcomes can be optimized through careful patient selection, surgical expertise, and multidisciplinary management. Moving forward, high‐quality, multicenter prospective studies are needed to address the limitations of the current evidence base, with standardized reporting of data, follow‐up, and outcomes. In particular, future studies should consistently report surgical margin status, neoadjuvant and adjuvant treatment regimens, and patient‐reported quality‐of‐life outcomes, and should apply uniform definitions for complications, recurrence, and survival to enable robust comparisons and strengthen clinical guidance for this challenging population.

## Conflicts of Interest

The authors declare no conflicts of interest.

## Synopsis

This systematic review and single‐arm meta‐analysis evaluated outcomes of limb sparing surgery with vascular reconstruction in patients with extremity soft tissue sarcomas involving major vessels. Across 31 studies and 520 patients, limb salvage was achieved in 89%, with acceptable long‐term survival and manageable morbidity. The findings support vascular reconstruction as a safe and effective strategy to preserve limb function without compromising oncologic outcomes.

## Supporting information

Supplementary Table S1. Preferred Reporting Items for Systematic Reviews and Meta‐Analysis (PRISMA) checklist. Supplementary Table S2. List of excluded studies at the full‐text screening stage and the reasons for exclusion.

## Data Availability

Data sharing not applicable to this article as no datasets were generated or analysed during the current study.
