# Supplementary material for: Vascular Reconstruction in Extremity Soft Tissue Sarcomas: A Systematic Review and Single‐Arm Meta‐Analysis
Source: J Surg Oncol. 2026 Jan 8;133(4):486–510. doi: 10.1002/jso.70194 (PMC12989194; doi:10.1002/jso.70194)
Supplement: Supplementary file 1 — Supplementary Table S1. Preferred Reporting Items for Systematic Reviews and Meta‐Analysis (PRISMA) checklist. Supplementary Table S2. List of excluded studies at the full‐text screening stage and the reasons for exclusion. [file JSO-133-486-s001.docx]

**SUPPLEMENTARY MATERIAL**

**TITLE:** Vascular Reconstruction in Extremity Soft Tissue Sarcomas: A Systematic Review and Single-Arm Meta-Analysis

**SUPPLEMENTARY TABLES AND FIGURES LEGENDS:**

**Supplementary Table S1.** Preferred Reporting Items for Systematic Reviews and Meta-Analysis (PRISMA) checklist.

**Supplementary Table S2.** List of excluded studies at the full-text screening stage and the reasons for exclusion.

**Supplementary Figure S1.** Forest plot of overall survival at 1 year.

**Supplementary Figure S2.** Forest plot of overall survival at 2 years.

**Supplementary Figure S3.** Forest plot of overall survival at 3 years.

**Supplementary Figure S4.** Forest plot of overall survival at 5 years.

**Supplementary Figure S5.** Forest plot of disease-free survival at 1 year.

**Supplementary Figure S6.** Forest plot of disease-free survival at 2 years.

**Supplementary Figure S7.** Forest plot of disease-free survival at 3 years.

**Supplementary Figure S8.** Forest plot of disease-free survival at 5 years.

**Supplementary Figure S9.** Forest plot of mortality related to the disease at any follow-up.

**Supplementary Figure S10.** Forest plot of local recurrence.

**Supplementary Figure S11.** Forest plot of distant metastasis.

**Supplementary Figure S12.** Forest plot of overall graft thrombosis.

**Supplementary Figure S13.** Forest plot of early graft thrombosis.

**Supplementary Figure S14.** Forest plot of late graft thrombosis.

**Supplementary Figure S15.** Forest plot of graft patency at 1 year.

**Supplementary Figure S16.** Forest plot of graft patency at 2 years.

**Supplementary Figure S17.** Forest plot of graft patency at 3 years.

**Supplementary Figure S18.** Forest plot of graft patency at 5 years.

**Supplementary Figure S19.** Forest plot of wound complication.

**Supplementary Figure S20.** Forest plot of wound infection.

**Supplementary Figure S21.** Leave-one-out analysis of overall survival at 1 year.

**Supplementary Figure S22.** Leave-one-out analysis of overall survival at 2 years.

**Supplementary Figure S23.** Leave-one-out analysis of overall survival at 3 years.

**Supplementary Figure S24.** Leave-one-out analysis of overall survival at 5 years.

**Supplementary Figure S25.** Leave-one-out analysis of disease-free survival at 1 year.

**Supplementary Figure S26.** Leave-one-out analysis of disease-free survival at 2 years.

**Supplementary Figure S27.** Leave-one-out analysis of disease-free survival at 3 years.

**Supplementary Figure S28.** Leave-one-out analysis of disease-free survival at 5 years.

**Supplementary Figure S29.** Leave-one-out analysis of distant metastasis.

**Supplementary Figure S30.** Leave-one-out analysis of overall graft thrombosis.

**Supplementary Figure S31.** Leave-one-out analysis of early graft thrombosis.

**Supplementary Figure S32.** Leave-one-out analysis of late graft thrombosis.

**Supplementary Figure S33.** Leave-one-out analysis of graft patency at 3 years.

**Supplementary Figure S34.** Leave-one-out analysis of graft patency at 5 years.

**Supplementary Figure S35.** Leave-one-out analysis of wound complication.

**Supplementary Figure S36.** Leave-one-out analysis of wound infection.

**Supplementary Figure S37.** Subgroup analysis for amputation based on cause.

**Supplementary Figure S38.** Subgroup analysis for limb salvage based on follow-up time.

**Supplementary Figure S39.** Subgroup analysis for limb salvage based on graft type.

**Supplementary Figure S40.** Subgroup analysis for limb salvage based on reconstruction type.

**Supplementary Table S1.** Preferred Reporting Items for Systematic Reviews and Meta-Analysis (PRISMA) checklist.

| **Section and Topic** | **Item #** | **Checklist item** | **Location where item is reported** |
| --- | --- | --- | --- |
| **TITLE** | | |  |
| Title | 1 | Identify the report as a systematic review. | Page 1 |
| **ABSTRACT** | | |  |
| Abstract | 2 | See the PRISMA 2020 for Abstracts checklist. | - |
| **INTRODUCTION** | | |  |
| Rationale | 3 | Describe the rationale for the review in the context of existing knowledge. | Page 4 |
| Objectives | 4 | Provide an explicit statement of the objective(s) or question(s) the review addresses. | Page 4 |
| **METHODS** | | |  |
| Eligibility criteria | 5 | Specify the inclusion and exclusion criteria for the review and how studies were grouped for the syntheses. | Page 5 |
| Information sources | 6 | Specify all databases, registers, websites, organisations, reference lists and other sources searched or consulted to identify studies. Specify the date when each source was last searched or consulted. | Pages 5-6 |
| Search strategy | 7 | Present the full search strategies for all databases, registers and websites, including any filters and limits used. | Pages 5-6 |
| Selection process | 8 | Specify the methods used to decide whether a study met the inclusion criteria of the review, including how many reviewers screened each record and each report retrieved, whether they worked independently, and if applicable, details of automation tools used in the process. | Pages 5-6 |
| Data collection process | 9 | Specify the methods used to collect data from reports, including how many reviewers collected data from each report, whether they worked independently, any processes for obtaining or confirming data from study investigators, and if applicable, details of automation tools used in the process. | Page 6 |
| Data items | 10a | List and define all outcomes for which data were sought. Specify whether all results that were compatible with each outcome domain in each study were sought (e.g. for all measures, time points, analyses), and if not, the methods used to decide which results to collect. | Page 5 |
|  | 10b | List and define all other variables for which data were sought (e.g. participant and intervention characteristics, funding sources). Describe any assumptions made about any missing or unclear information. | Page 7 |
| Study risk of bias assessment | 11 | Specify the methods used to assess risk of bias in the included studies, including details of the tool(s) used, how many reviewers assessed each study and whether they worked independently, and if applicable, details of automation tools used in the process. | Page 6 |
| Effect measures | 12 | Specify for each outcome the effect measure(s) (e.g. risk ratio, mean difference) used in the synthesis or presentation of results. | Pages 6-7 |
| Synthesis methods | 13a | Describe the processes used to decide which studies were eligible for each synthesis (e.g. tabulating the study intervention characteristics and comparing against the planned groups for each synthesis (item #5)). | Page 5 |
|  | 13b | Describe any methods required to prepare the data for presentation or synthesis, such as handling of missing summary statistics, or data conversions. | Pages 6-7 |
|  | 13c | Describe any methods used to tabulate or visually display results of individual studies and syntheses. | Pages 6-7 |
|  | 13d | Describe any methods used to synthesize results and provide a rationale for the choice(s). If meta-analysis was performed, describe the model(s), method(s) to identify the presence and extent of statistical heterogeneity, and software package(s) used. | Pages 6-7 |
|  | 13e | Describe any methods used to explore possible causes of heterogeneity among study results (e.g. subgroup analysis, meta-regression). | Pages 6-7 |
|  | 13f | Describe any sensitivity analyses conducted to assess robustness of the synthesized results. | Pages 6-7 |
| Reporting bias assessment | 14 | Describe any methods used to assess risk of bias due to missing results in a synthesis (arising from reporting biases). | Page 6 |
| Certainty assessment | 15 | Describe any methods used to assess certainty (or confidence) in the body of evidence for an outcome. | Page 6 |
| **RESULTS** | | |  |
| Study selection | 16a | Describe the results of the search and selection process, from the number of records identified in the search to the number of studies included in the review, ideally using a flow diagram. | Page 7 |
|  | 16b | Cite studies that might appear to meet the inclusion criteria, but which were excluded, and explain why they were excluded. | Figure 1 |
| Study characteristics | 17 | Cite each included study and present its characteristics. | Table 1 |
| Risk of bias in studies | 18 | Present assessments of risk of bias for each included study. | Supplementary Table S3 |
| Results of individual studies | 19 | For all outcomes, present, for each study: (a) summary statistics for each group (where appropriate) and (b) an effect estimate and its precision (e.g. confidence/credible interval), ideally using structured tables or plots. | Pages 8-9 |
| Results of syntheses | 20a | For each synthesis, briefly summarise the characteristics and risk of bias among contributing studies. | Pages 8-9 |
|  | 20b | Present results of all statistical syntheses conducted. If meta-analysis was done, present for each the summary estimate and its precision (e.g. confidence/credible interval) and measures of statistical heterogeneity. If comparing groups, describe the direction of the effect. | Pages 8-9 |
|  | 20c | Present results of all investigations of possible causes of heterogeneity among study results. | Pages 8-9 |
|  | 20d | Present results of all sensitivity analyses conducted to assess the robustness of the synthesized results. | Supplementary Material |
| Reporting biases | 21 | Present assessments of risk of bias due to missing results (arising from reporting biases) for each synthesis assessed. | - |
| Certainty of evidence | 22 | Present assessments of certainty (or confidence) in the body of evidence for each outcome assessed. | Table 2 |
| **DISCUSSION** | | |  |
| Discussion | 23a | Provide a general interpretation of the results in the context of other evidence. | Pages 9-11 |
|  | 23b | Discuss any limitations of the evidence included in the review. | Pages 9-11 |
|  | 23c | Discuss any limitations of the review processes used. | Pages 9-11 |
|  | 23d | Discuss implications of the results for practice, policy, and future research. | Pages 9-11 |
| **OTHER INFORMATION** | | |  |
| Registration and protocol | 24a | Provide registration information for the review, including register name and registration number, or state that the review was not registered. | - |
|  | 24b | Indicate where the review protocol can be accessed, or state that a protocol was not prepared. | - |
|  | 24c | Describe and explain any amendments to information provided at registration or in the protocol. | - |
| Support | 25 | Describe sources of financial or non-financial support for the review, and the role  of the funders or sponsors in the review. | Page 18 |
| Competing interests | 26 | Declare any competing interests of review authors. | Page 18 |
| Availability of data, code and other materials | 27 | Report which of the following are publicly available and where they can be found: template data collection forms; data extracted from included studies; data used for all analyses; analytic code; any other materials used in the review. | Page 18 |

*From:*  Page MJ, McKenzie JE, Bossuyt PM, Boutron I, Hoffmann TC, Mulrow CD, et al. The PRISMA 2020 statement: an updated guideline for reporting systematic reviews. BMJ 2021;372:n71. doi: 10.1136/bmj.n71

**Supplementary Table S2.** List of excluded studies at the full-text screening stage and the reasons for exclusion.

| **First author, year [ref]** | **Reason for exclusion** |
| --- | --- |
| Bertrand, 2016 [1] | Only retroperitoneal sarcomas |
| Radaelli, 2016 [2] | Retroperitoneal and extremity sarcomas not analyzed separately |
| Song, 2009 [3] | Retroperitoneal and extremity sarcomas not analyzed separately |
| Angelini, 2021 [4] | Not all patients underwent vascular reconstruction or outcomes not stratified |
| Sternheim, 2009 [5] | Not all patients underwent vascular reconstruction or outcomes not stratified |
| Gallaway, 2020 [6] | Data not clearly separated for vascular reconstruction cases |
| Kekeç, 2013 [7] | Duplicate publication |
| Imparato, 1978 [8] | Full text not retrievable |
| Nambisan, 1987 [9] | Full text not retrievable |
| Steed, 1987 [10] | Full text not retrievable |
| El-Zohairy, 2008 [11] | Full text not retrievable |

1. Bertrand MM, Carrère S, Delmond L, Mehta S, Rouanet P, Canaud L, Alric P, Quénet F. Oncovascular compartmental resection for retroperitoneal soft tissue sarcoma with vascular involvement. J Vasc Surg. 2016 Oct;64(4):1033-41. doi: 10.1016/j.jvs.2016.04.006. Epub 2016 Jun 30. PMID: 27374069.
2. Radaelli S, Fiore M, Colombo C, Ford S, Palassini E, Sanfilippo R, Stacchiotti S, Sangalli C, Morosi C, Casali PG, Gronchi A. Vascular resection en-bloc with tumor removal and graft reconstruction is safe and effective in soft tissue sarcoma (STS) of the extremities and retroperitoneum. Surg Oncol. 2016 Sep;25(3):125-31. doi: 10.1016/j.suronc.2016.05.002. Epub 2016 May 7. PMID: 27566012.
3. Song TK, Harris EJ Jr, Raghavan S, Norton JA. Major blood vessel reconstruction during sarcoma surgery. Arch Surg. 2009 Sep;144(9):817-22. doi: 10.1001/archsurg.2009.149. PMID: 19797105.
4. Angelini A, Piazza M, Pagliarini E, Trovarelli G, Spertino A, Ruggieri P. The Orthopedic-Vascular Multidisciplinary Approach Improves Patient Safety in Surgery for Musculoskeletal Tumors: A Large-Volume Center Experience. J Pers Med. 2021 May 24;11(6):462. doi: 10.3390/jpm11060462. PMID: 34073954; PMCID: PMC8225121.
5. Sternheim, Amira; Bickels, Jacoba; Ben-Tov, Tomera; Malawer, Martin Ma,b,c. Primary tumors of the sartorial canal: limb sparing resection of soft-tissue sarcomas arising in a unique location. Current Orthopaedic Practice 20(4):p 416-422, August 2009. | DOI: 10.1097/BCO.0b013e318199ccfa
6. Gallaway KE, Ahn J, Callan AK. Thirty-Day Outcomes after Surgery for Primary Sarcomas of the Extremities: An Analysis of the NSQIP Database. J Oncol. 2020 Jan 13;2020:7282846. doi: 10.1155/2020/7282846. PMID: 32411242; PMCID: PMC7201584.
7. Zheng C, Xu G, Li W, Weng X, Yang H, Wang Z, Zhang S. Clinical outcomes after vascular reconstruction using synthetic grafts for limb salvage in patients with lower extremity sarcoma: a single-center retrospective experience. Front Oncol. 2023 Aug 17;13:1199556. doi: 10.3389/fonc.2023.1199556. PMID: 37664022; PMCID: PMC10471183.
8. Imparato AM, Roses DF, Francis KC, Lewis MM. Major vascular reconstruction for limb salvage in patients with soft tissue and skeletal sarcomas of the extremities. Surg Gynecol Obstet. 1978 Dec;147(6):891-6. PMID: 280963.
9. Nambisan RN, Karakousis CP. Vascular reconstruction for limb salvage in soft tissue sarcomas. Surgery. 1987 Jun;101(6):668-77. PMID: 3589963.
10. Steed DL, Peitzman AB, Webster MW Jr, Ramasastry SS, Goodman MA. Limb sparing operations for sarcomas of the extremities involving critical arterial circulation. Surg Gynecol Obstet. 1987 Jun;164(6):493-8. PMID: 3473704.
11. El-Zohairy MA, El-Samadoni A, El-Sherbieny ME, Anwar H. Limb-sparing surgery with vascular reconstruction for extremity soft tissue sarcomas: surgical, oncological and functional outcomes. J Egypt Natl Canc Inst. 2008 Sep;20(3):244-52. PMID: 20424655.

**
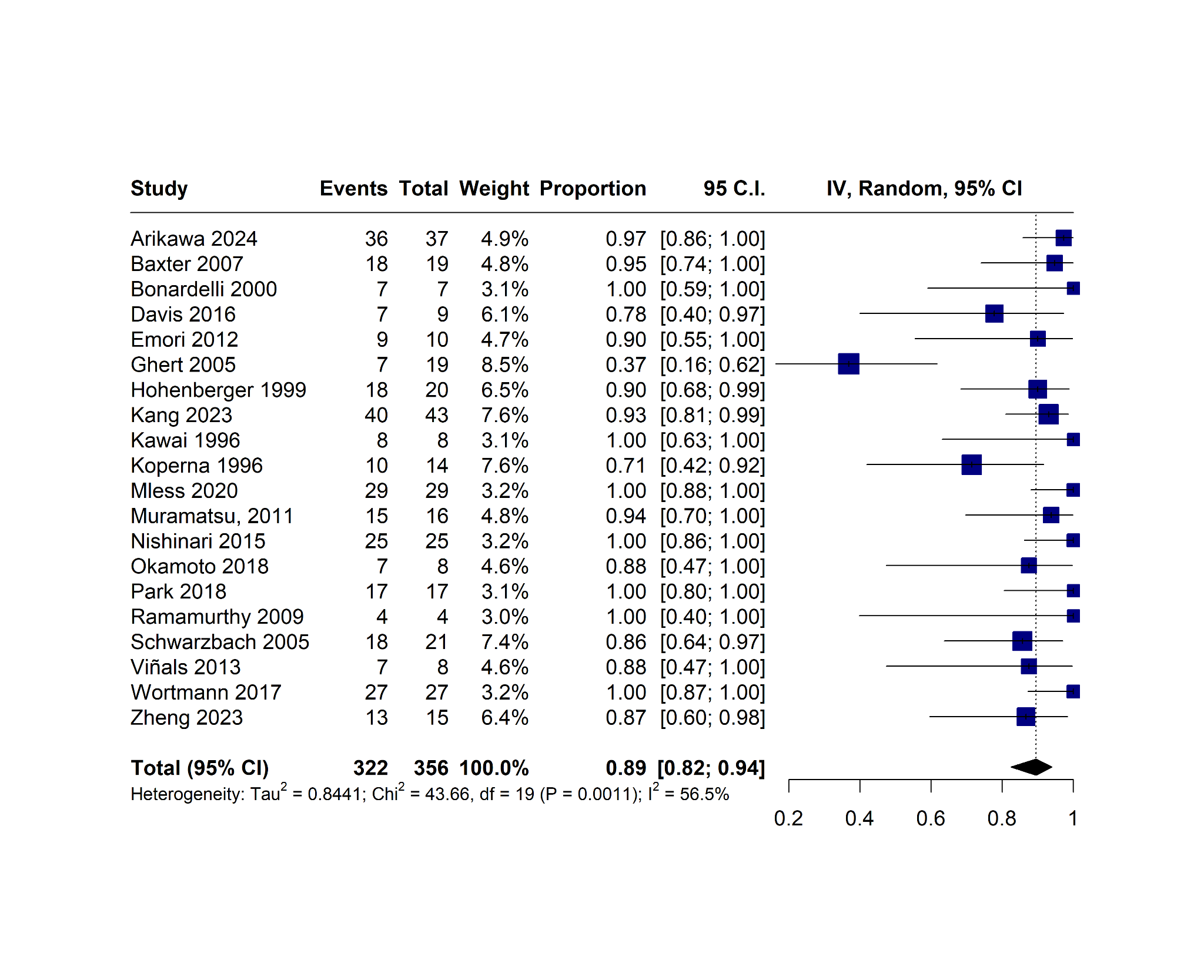
**

**Supplementary Figure S1.** Forest plot of overall survival at 1 year.

**
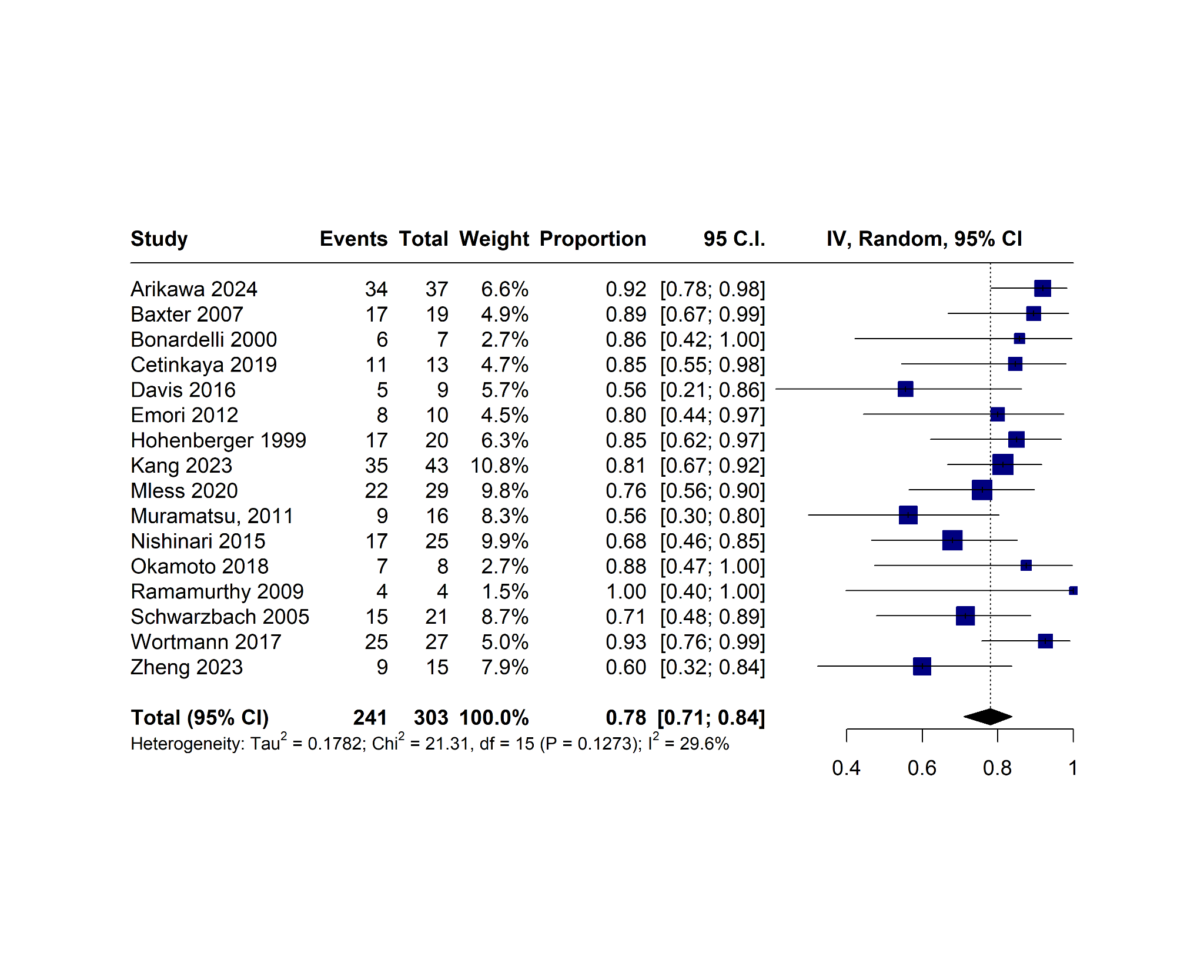
**

**Supplementary Figure S2.** Forest plot of overall survival at 2 years.

**
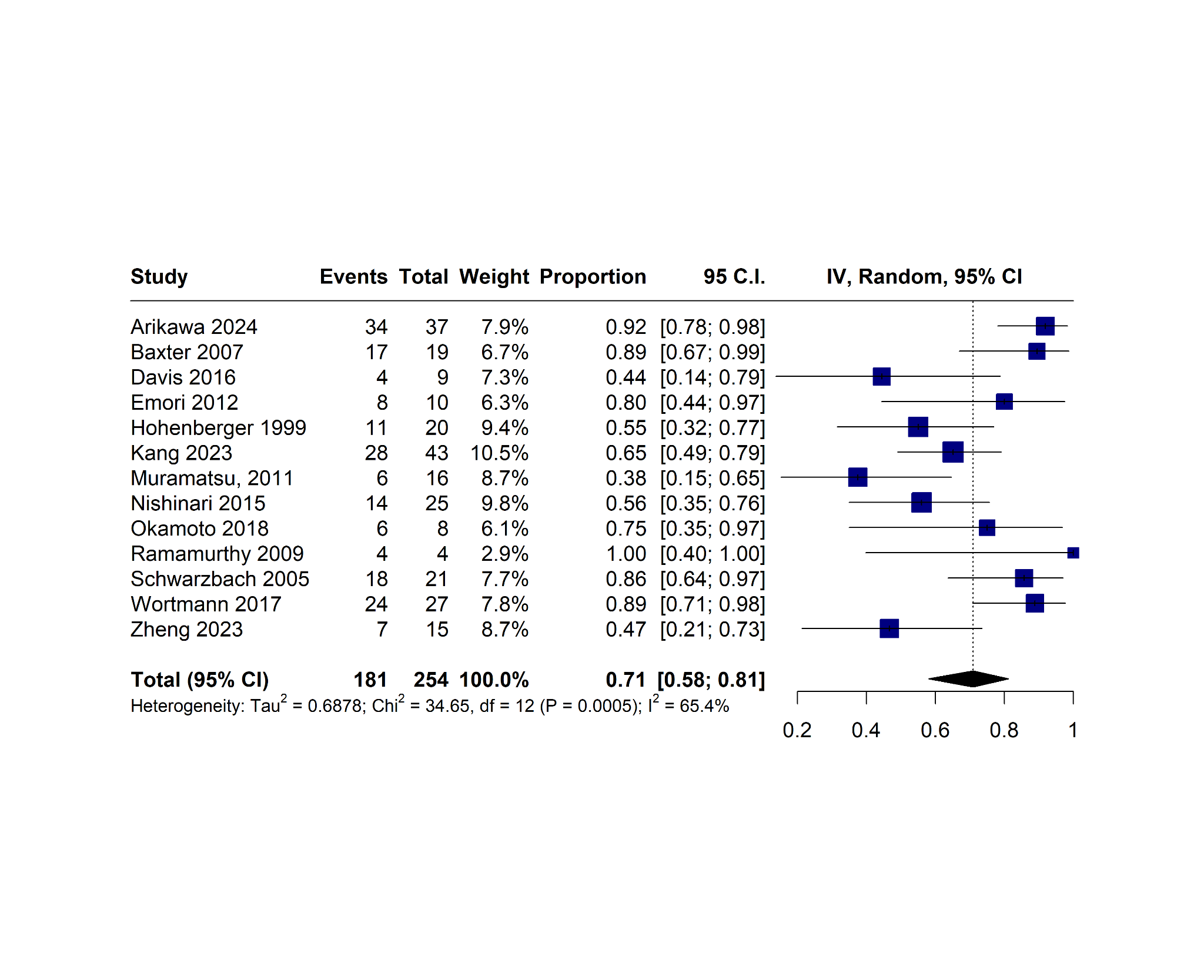
**

**Supplementary Figure S3.** Forest plot of overall survival at 3 years.

**
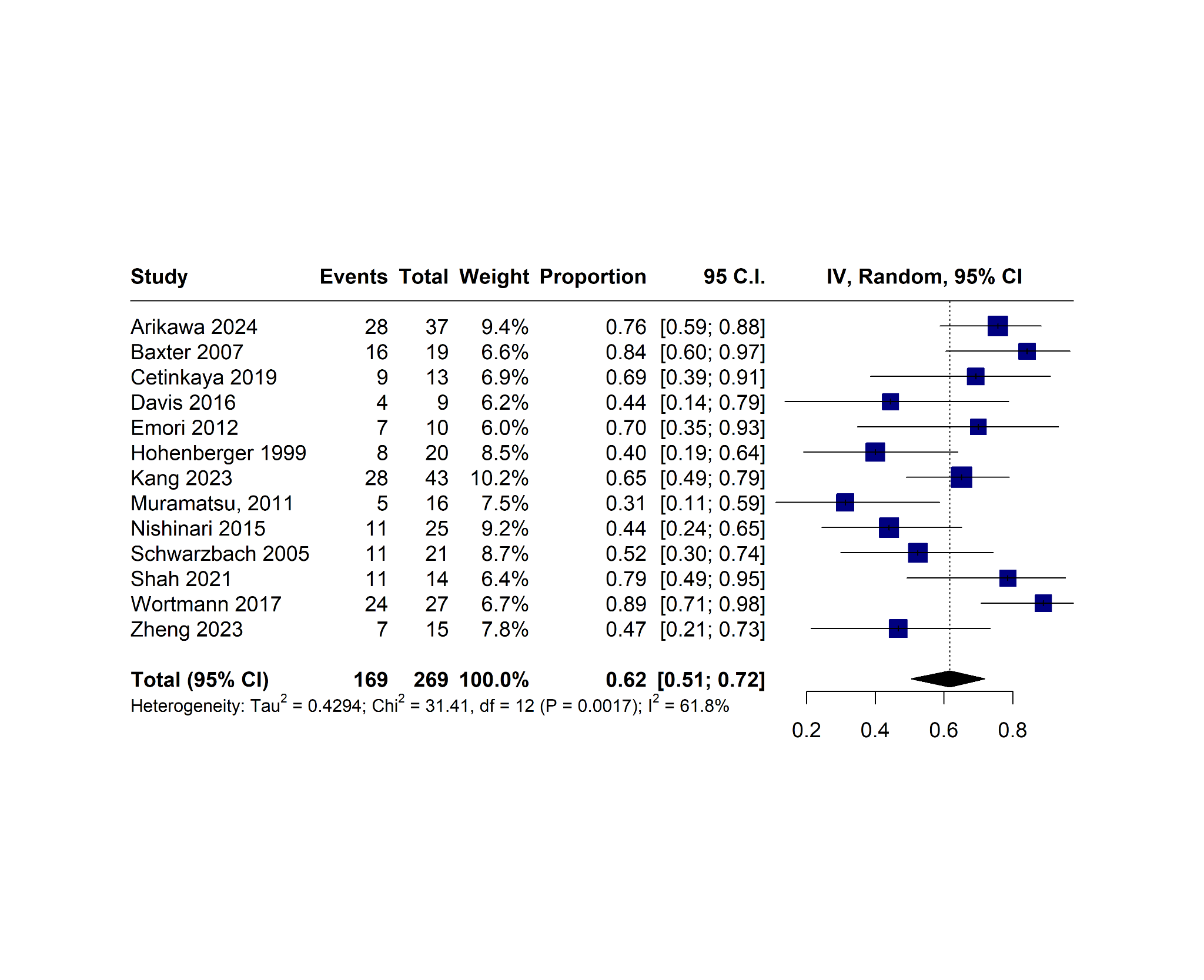
**

**Supplementary Figure S4.** Forest plot of overall survival at 5 years.

**
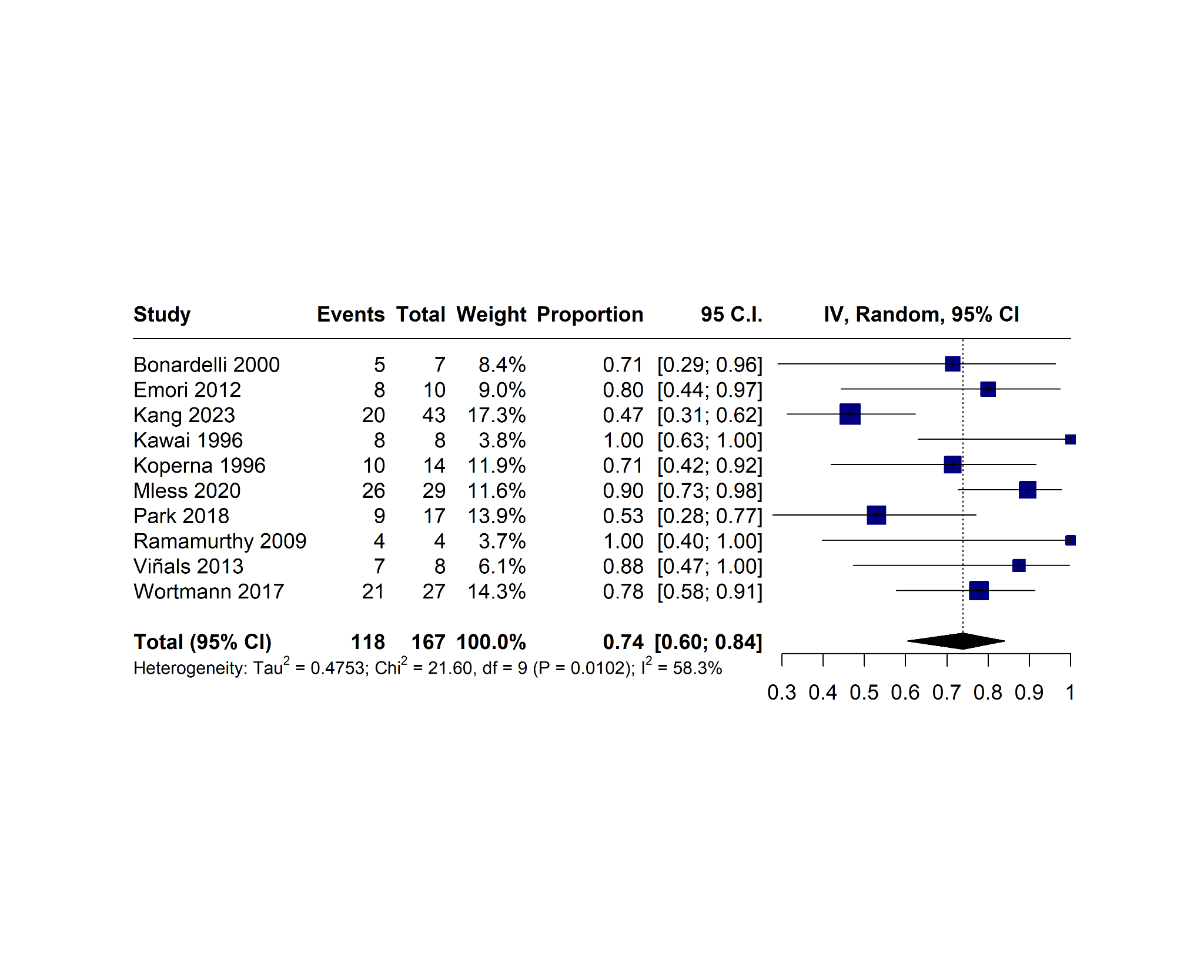
**

**Supplementary Figure S5.** Forest plot of disease-free survival at 1 year.

**
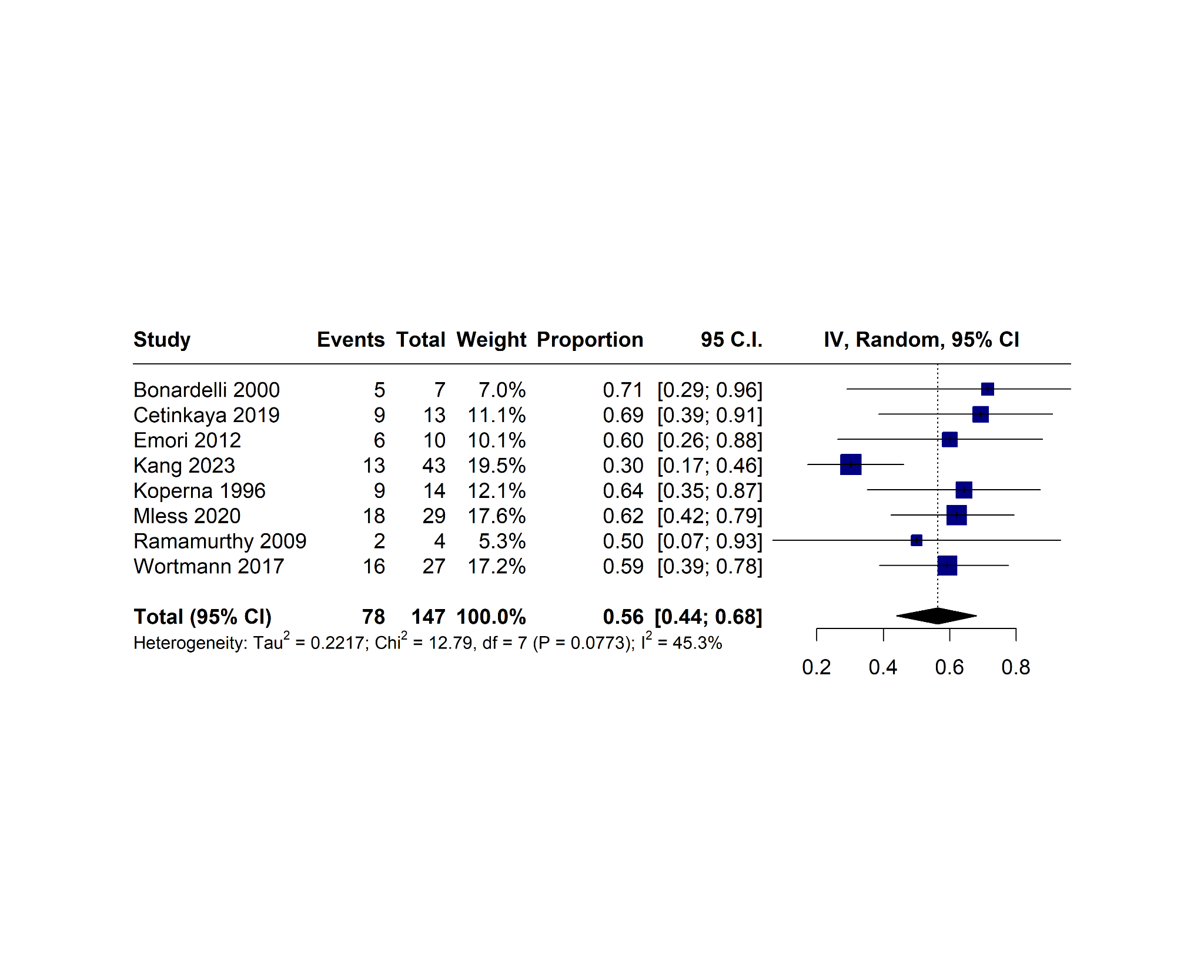
**

**Supplementary Figure S6.** Forest plot of disease-free survival at 2 years.

**
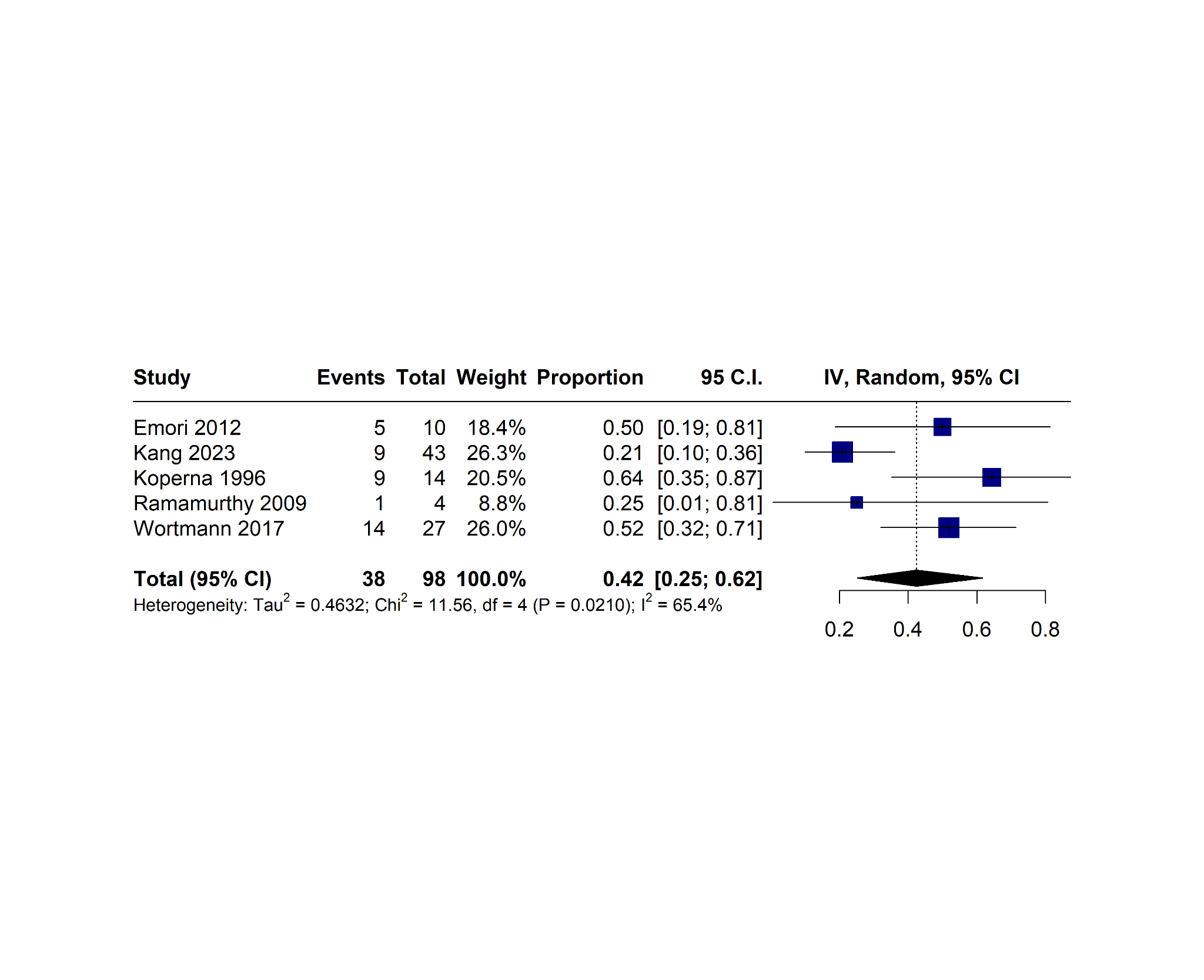
**

**Supplementary Figure S7.** Forest plot of disease-free survival at 3 years.

**
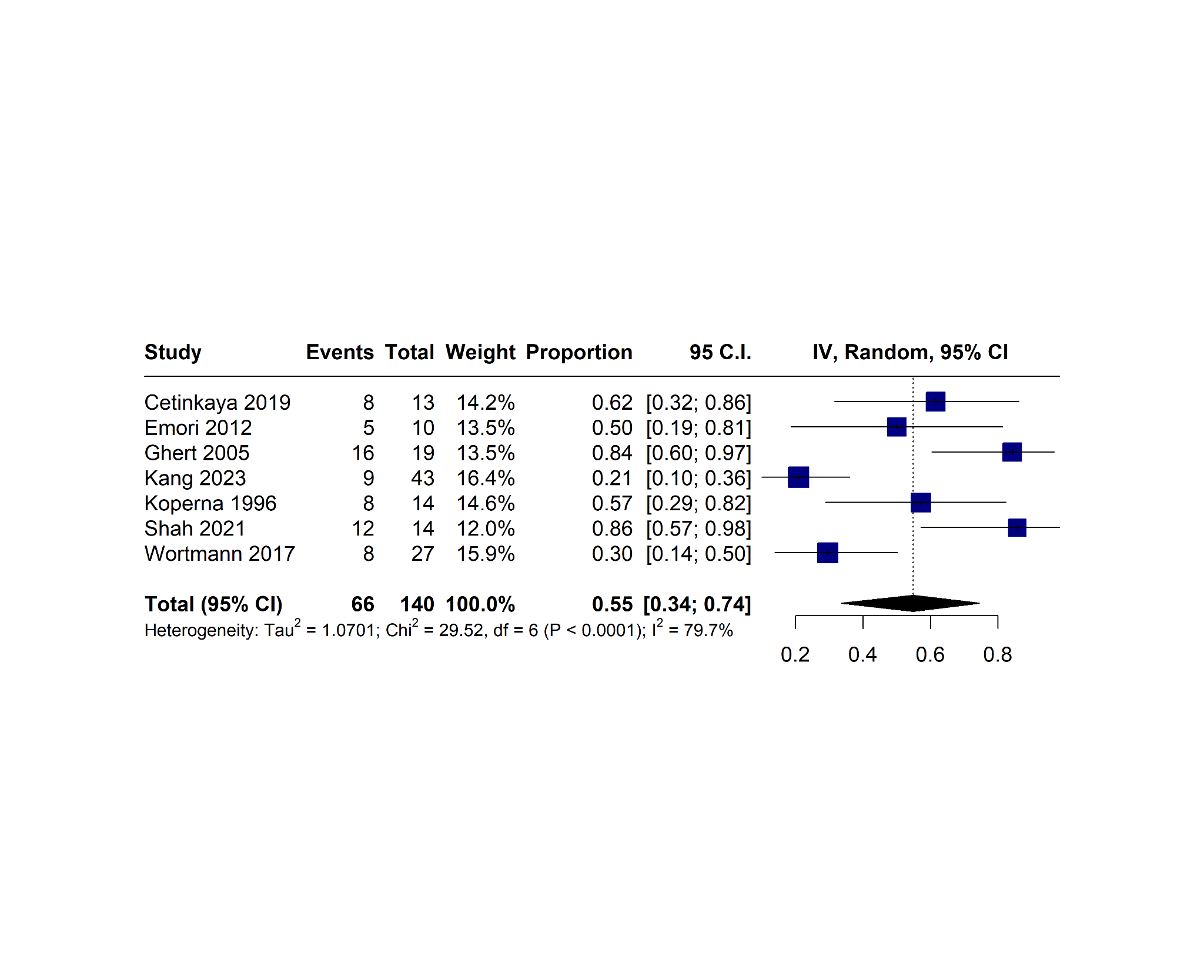
**

**Supplementary Figure S8.** Forest plot of disease-free survival at 5 years.

**
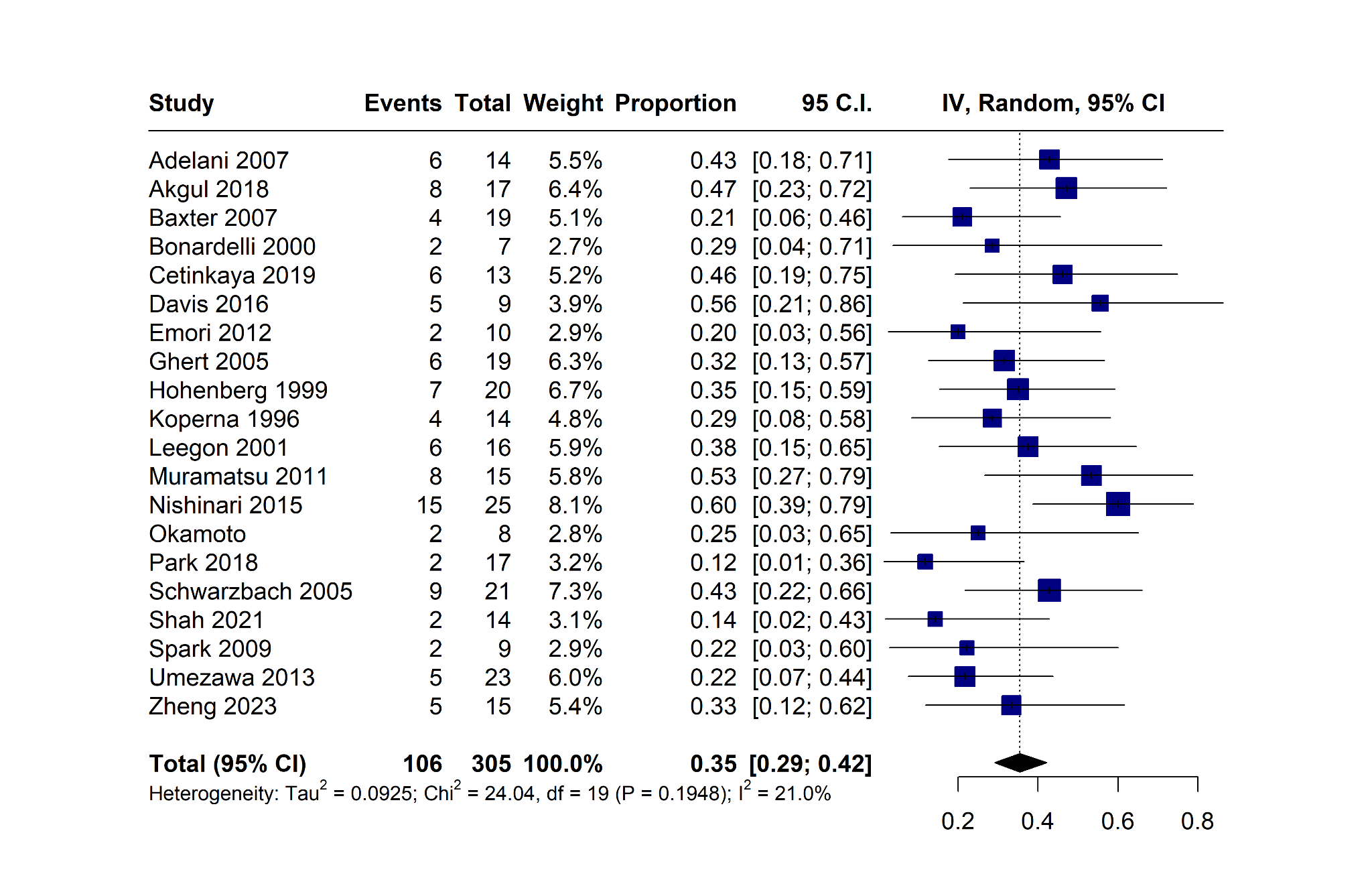
**

**Supplementary Figure S9.** Forest plot of mortality related to the disease at any follow-up.

**
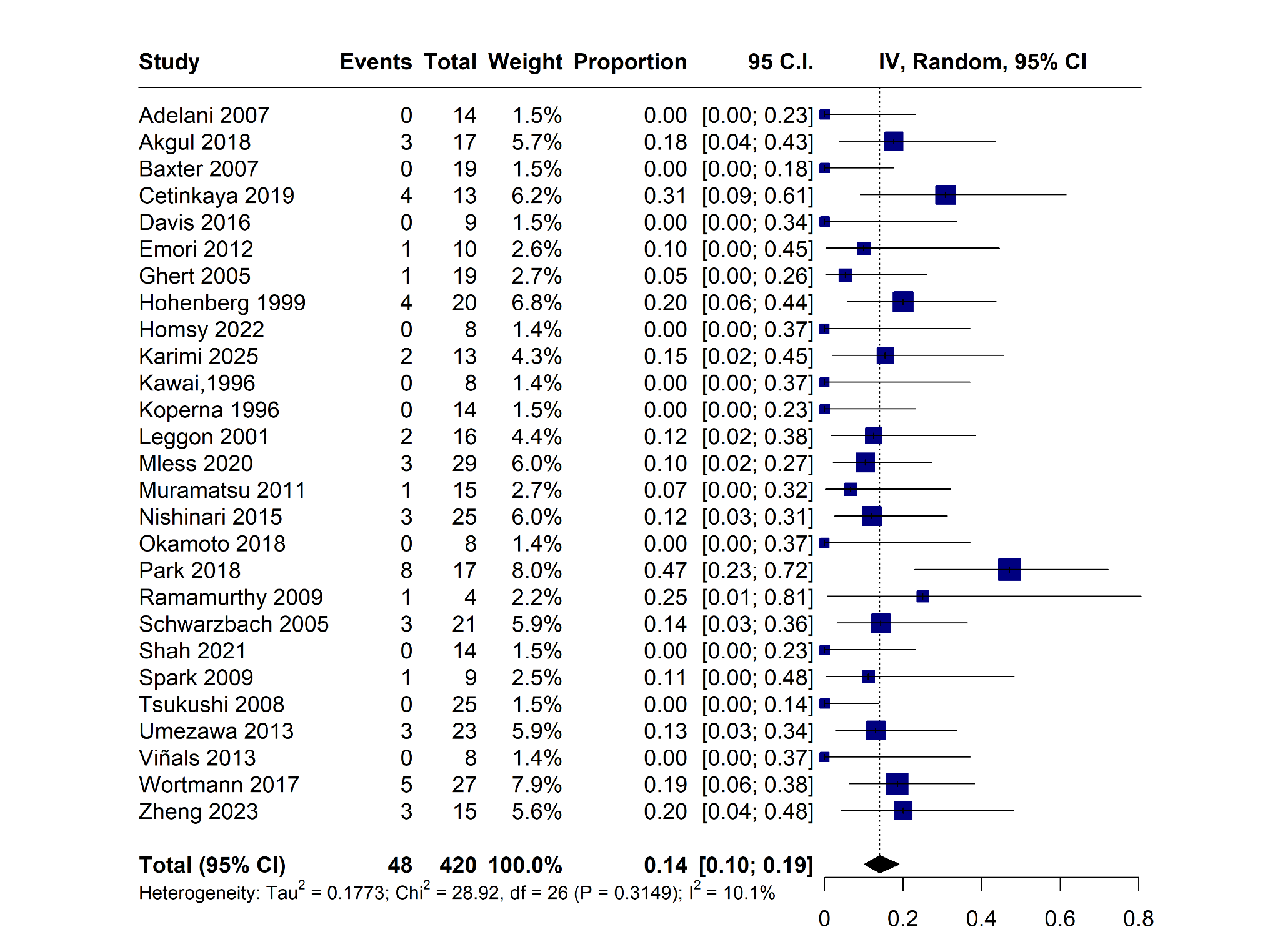
**

**Supplementary Figure S10.** Forest plot of local recurrence.

**
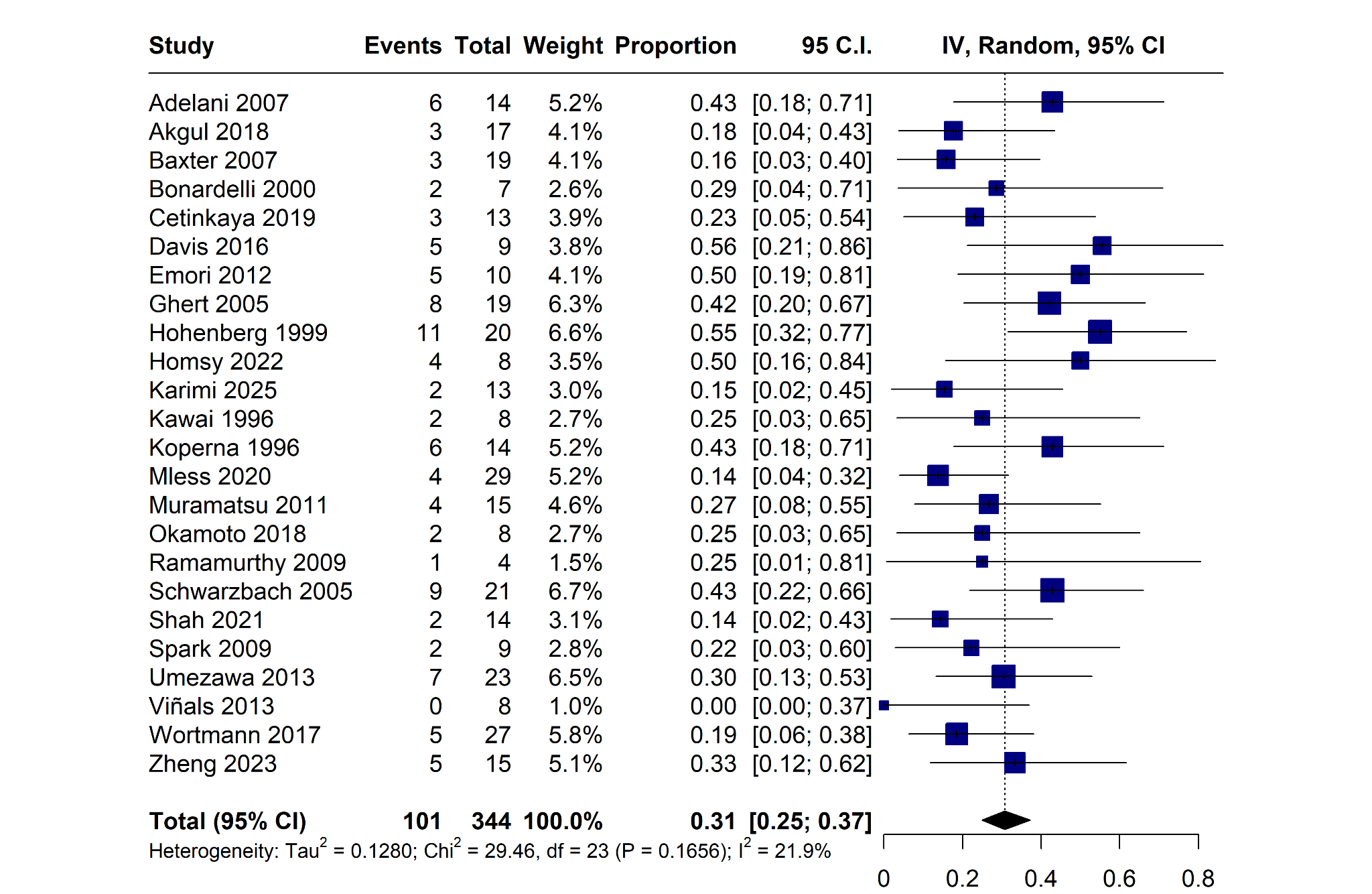
**

**Supplementary Figure S11.** Forest plot of distant metastasis.

**
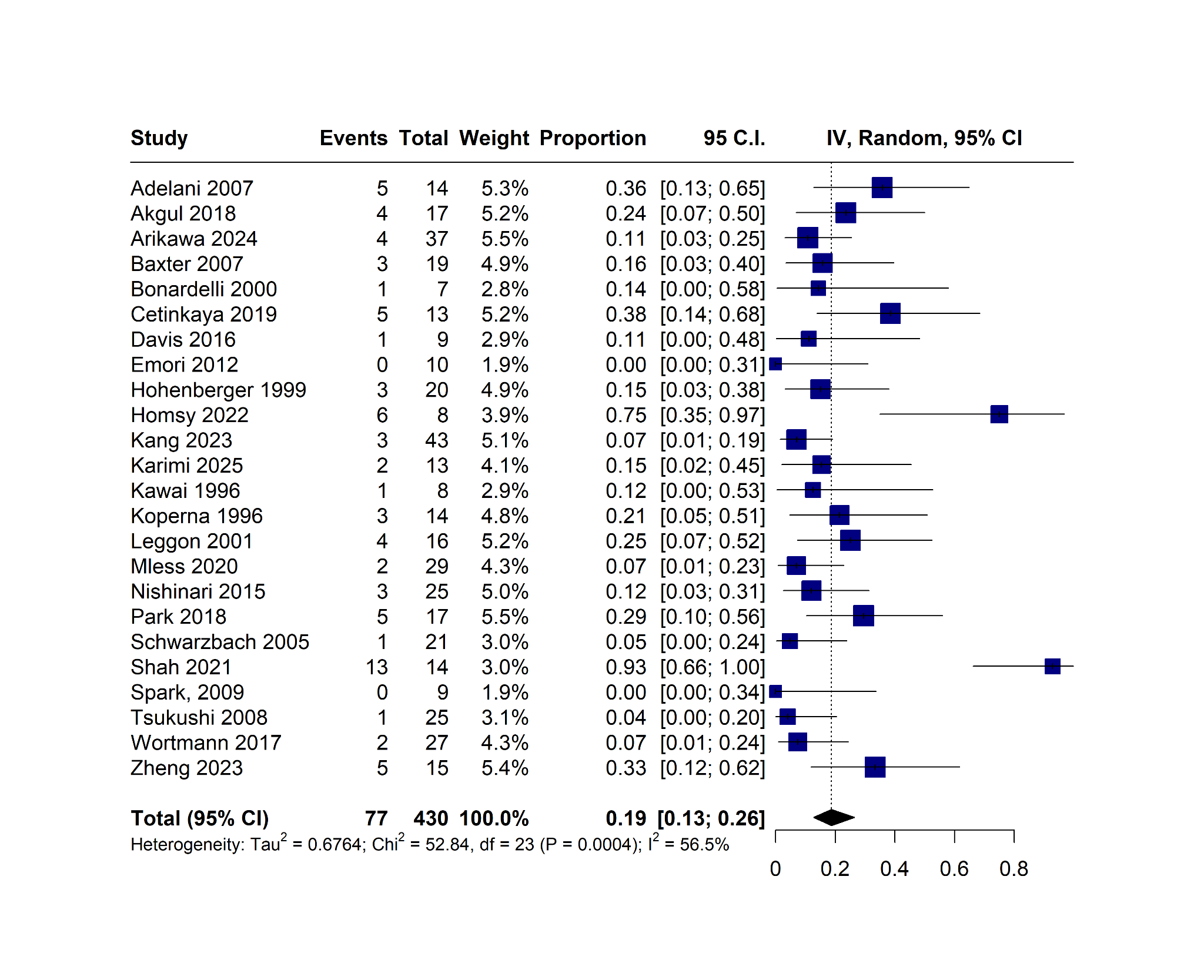
**

**Supplementary Figure S12.** Forest plot of overall graft thrombosis.


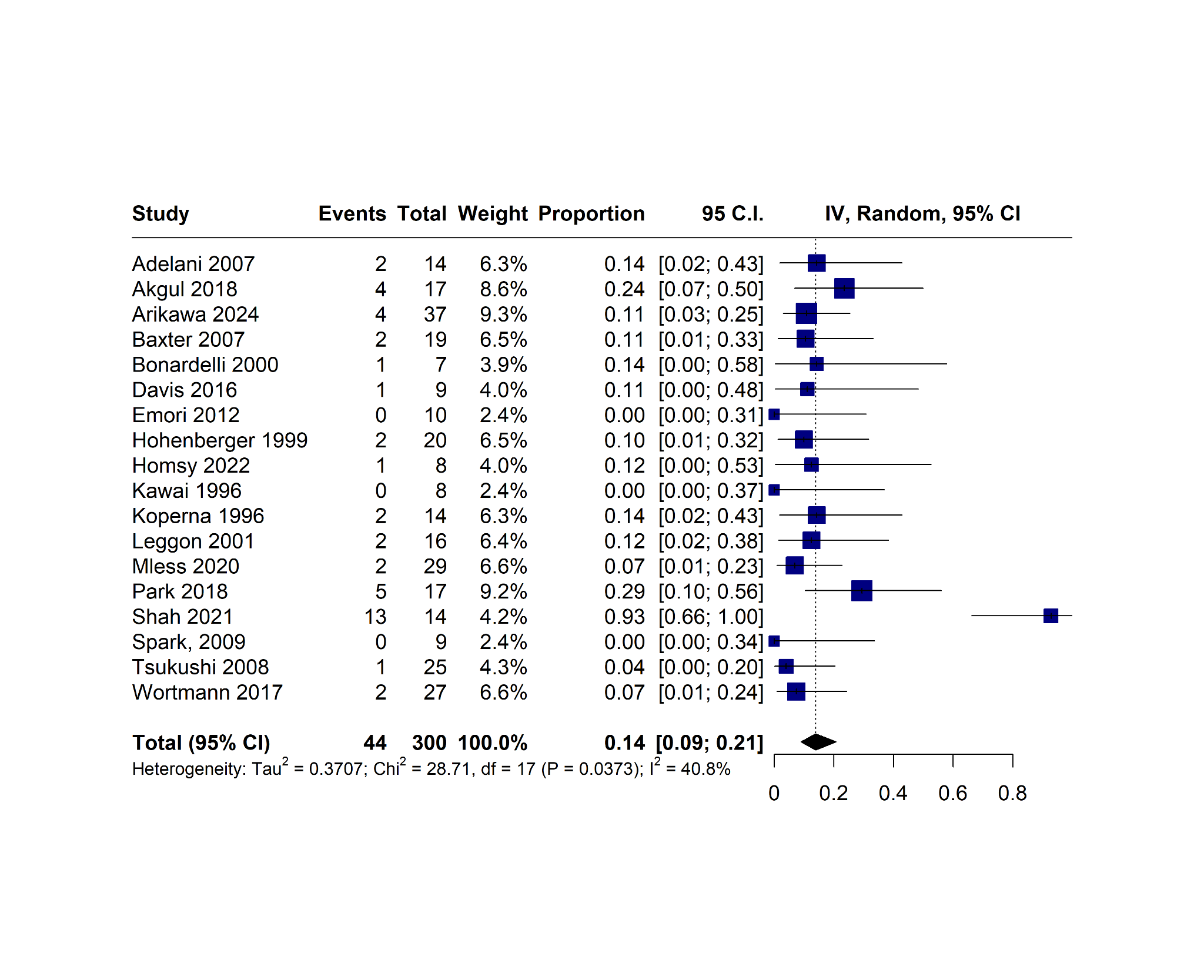


**Supplementary Figure S13.** Forest plot of early graft thrombosis.

**
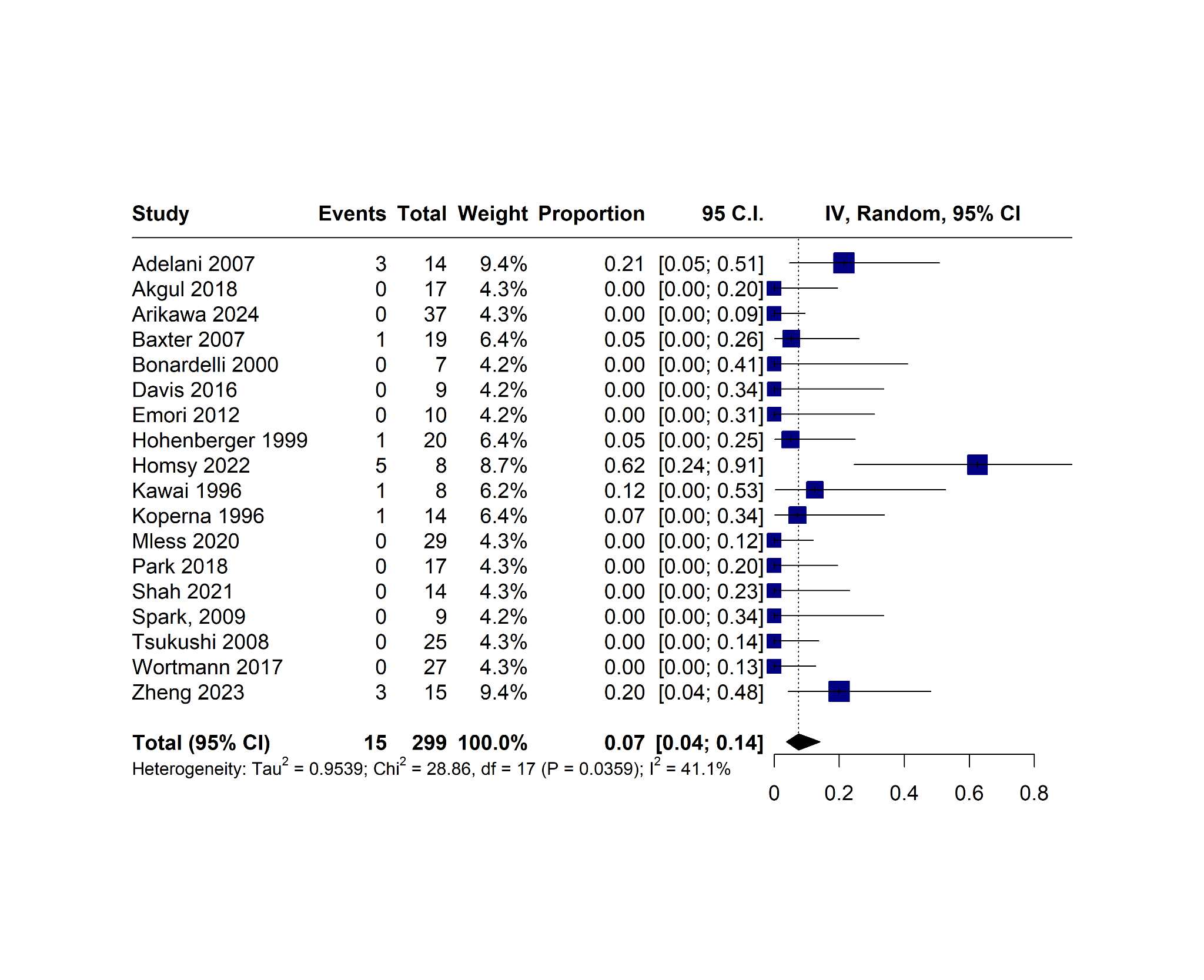
**

**Supplementary Figure S14.** Forest plot of late graft thrombosis.


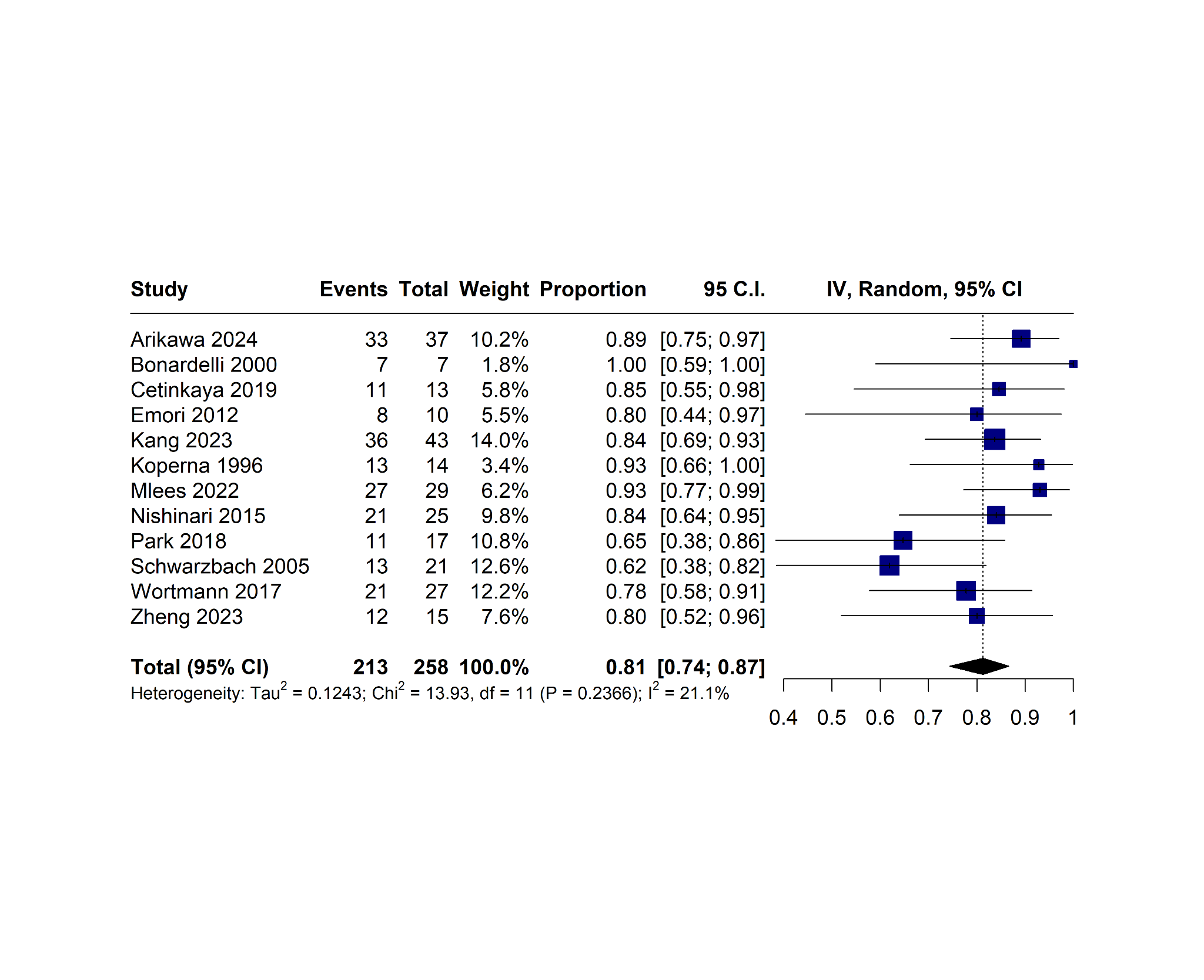


**Supplementary Figure S15.** Forest plot of graft patency at 1 year.


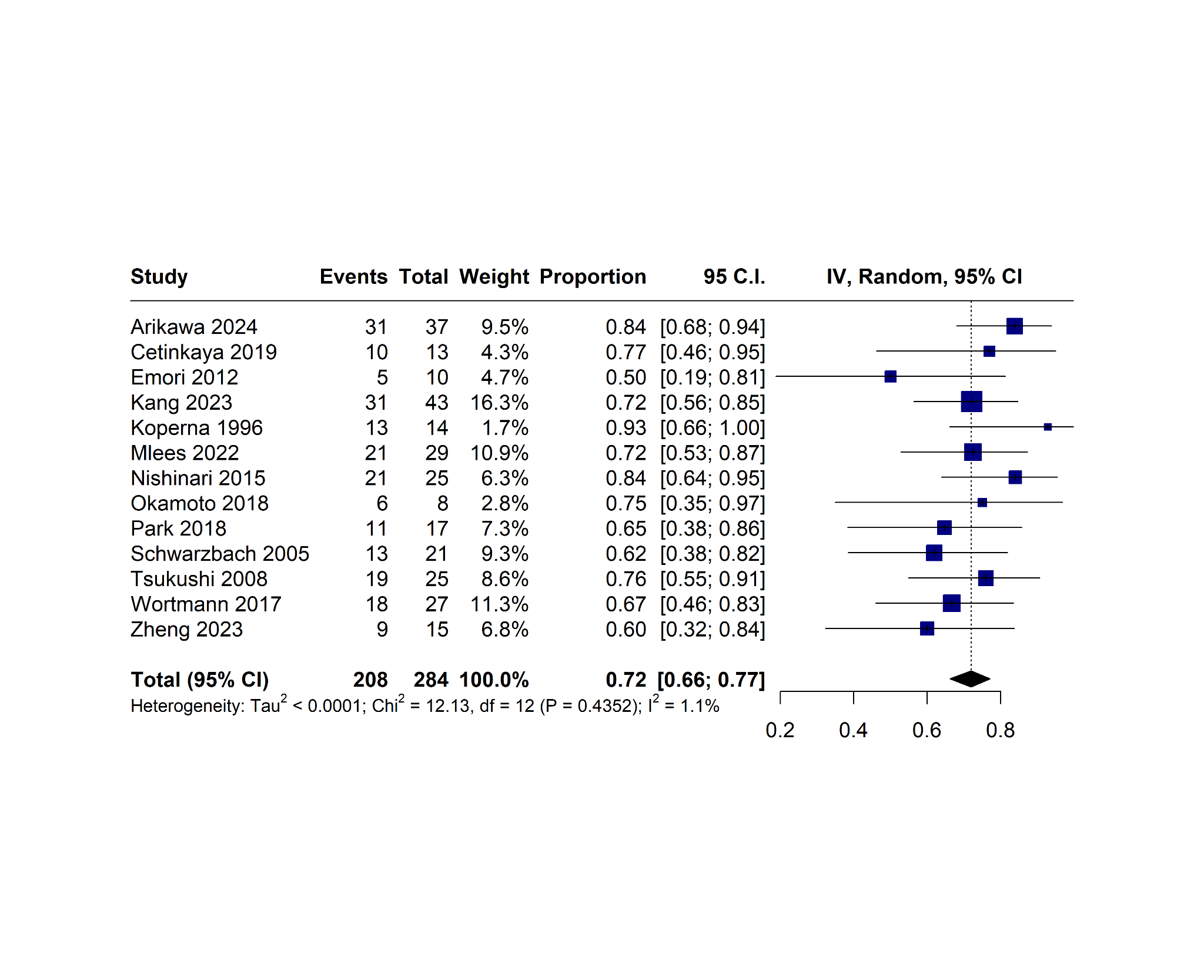


**Supplementary Figure S16.** Forest plot of graft patency at 2 years.


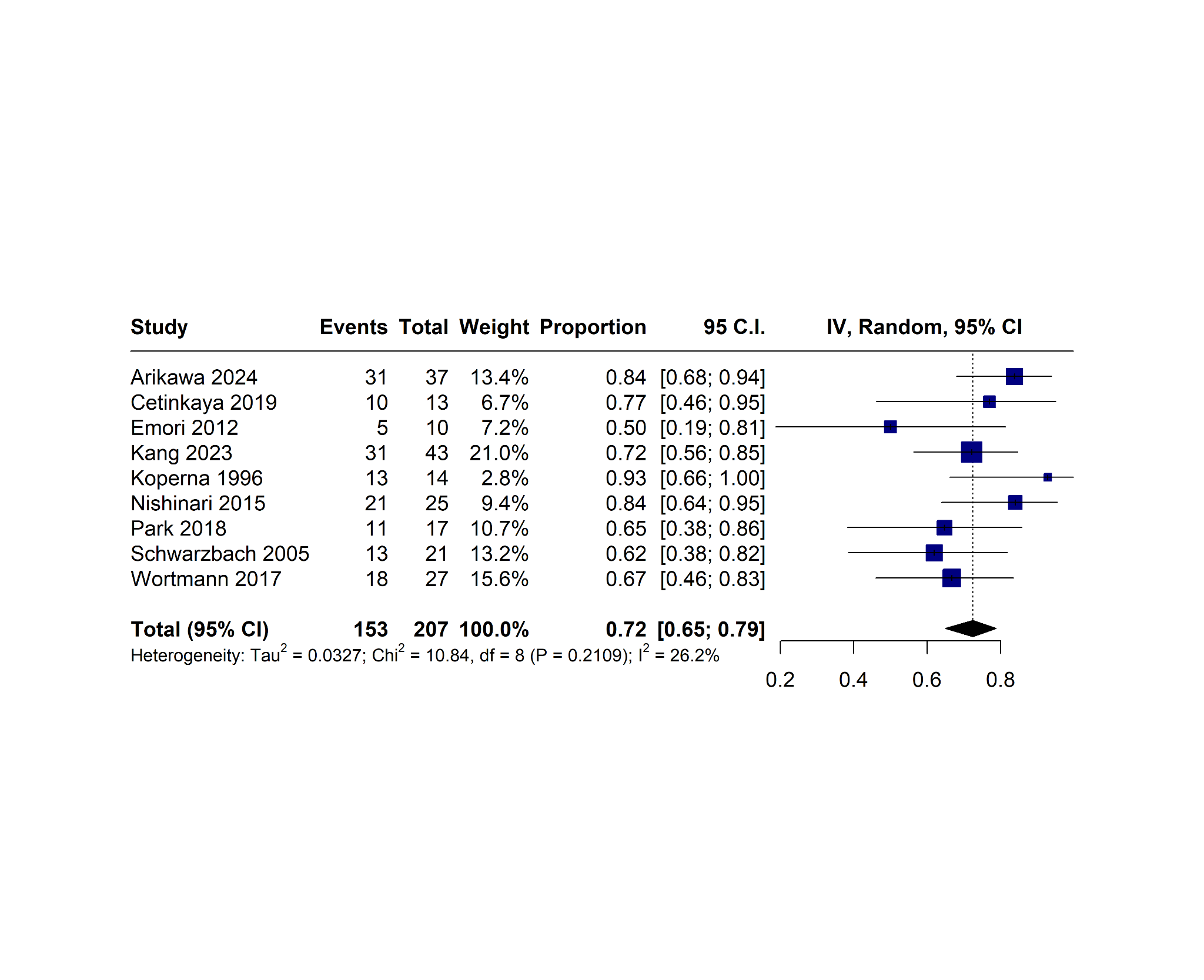


**Supplementary Figure S17.** Forest plot of graft patency at 3 years.


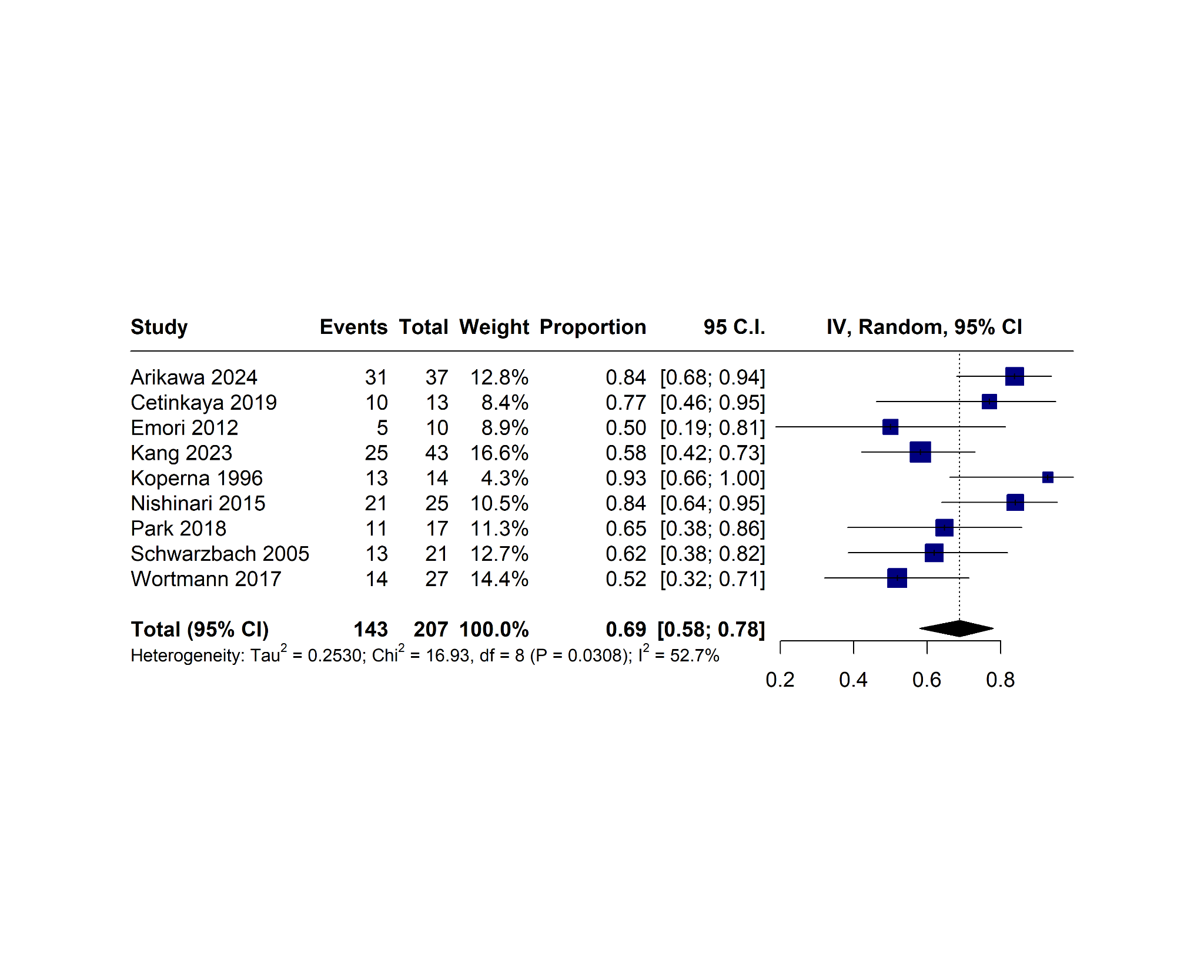


**Supplementary Figure S18.** Forest plot of graft patency at 5 years.


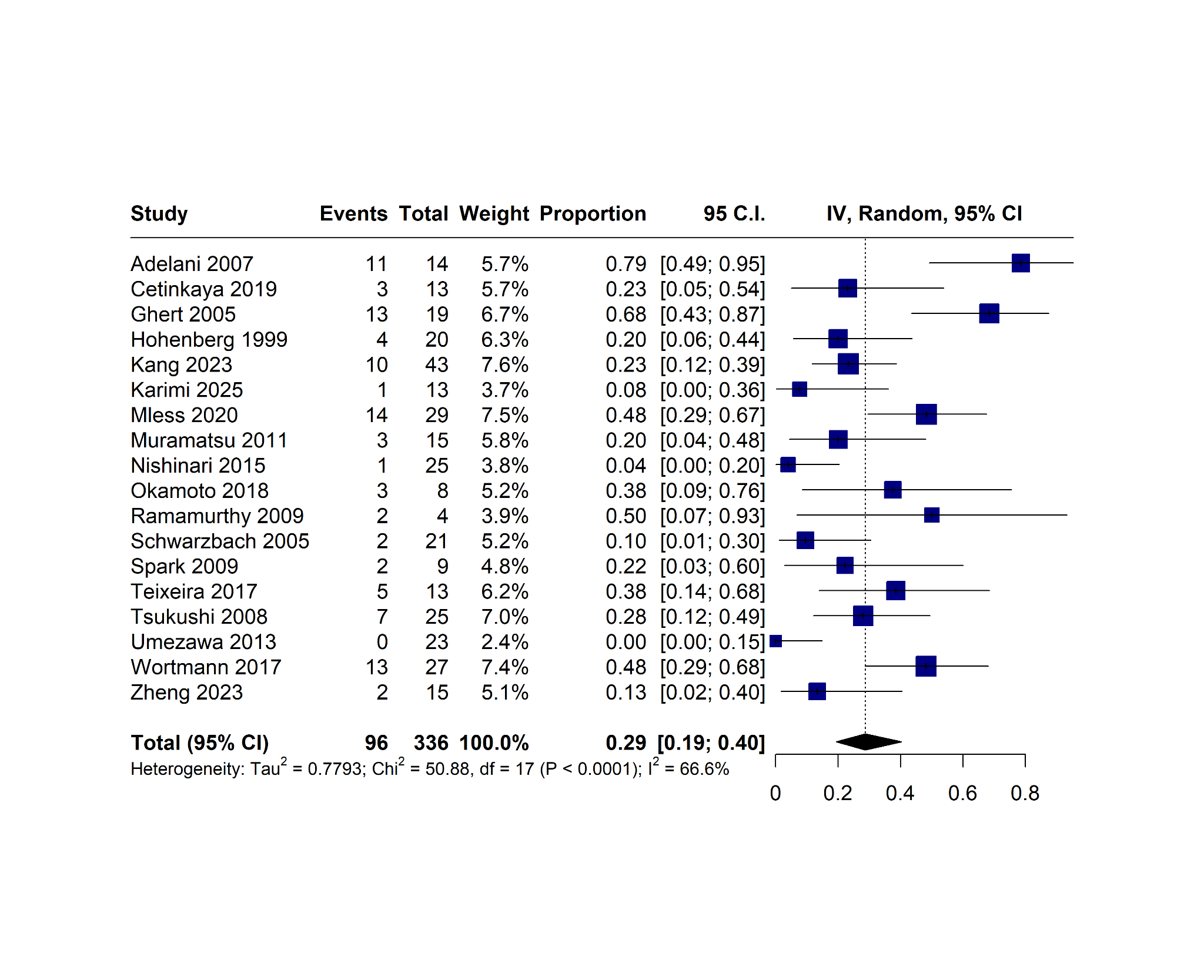


**Supplementary Figure S19.** Forest plot of wound complication.


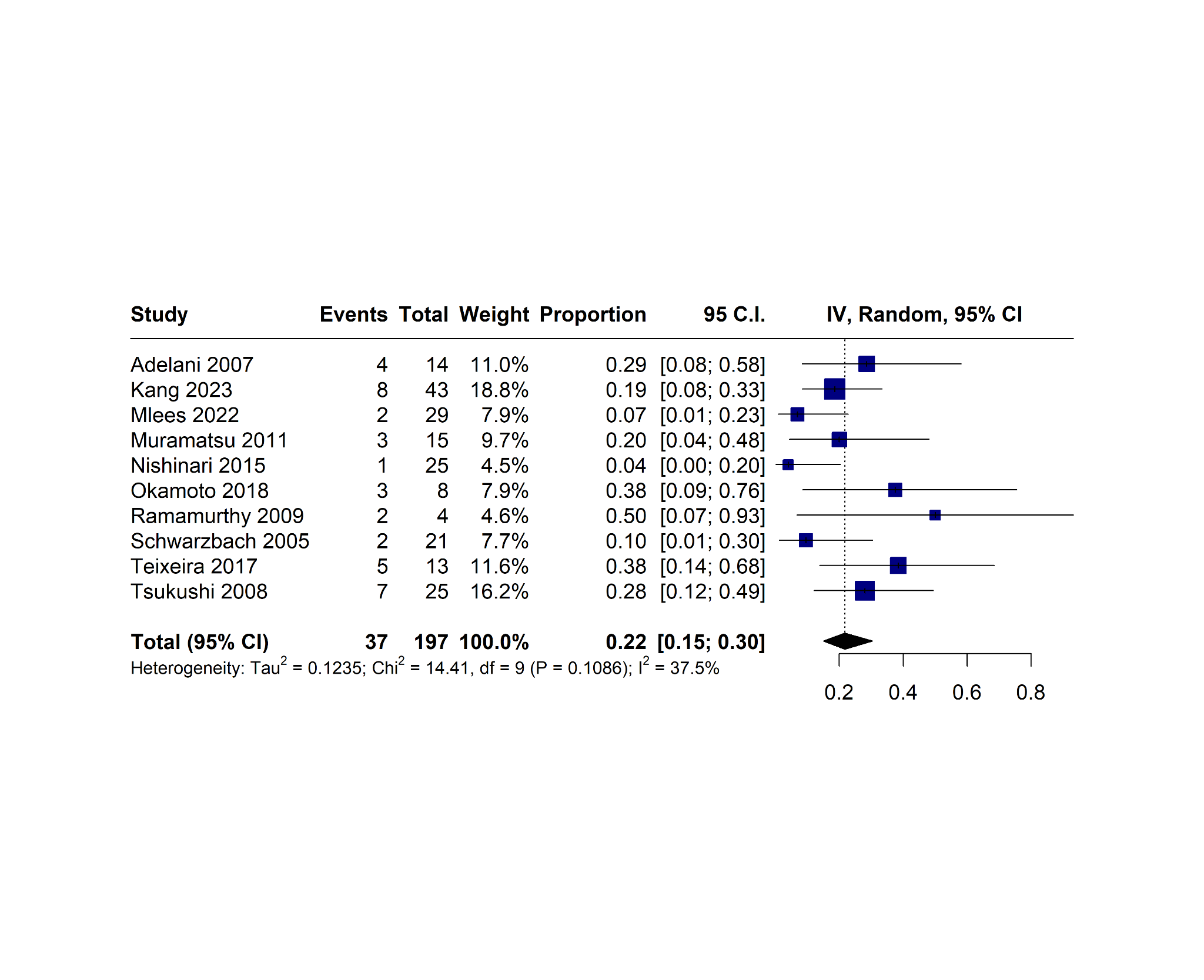


**Supplementary Figure S20.** Forest plot of wound infection.


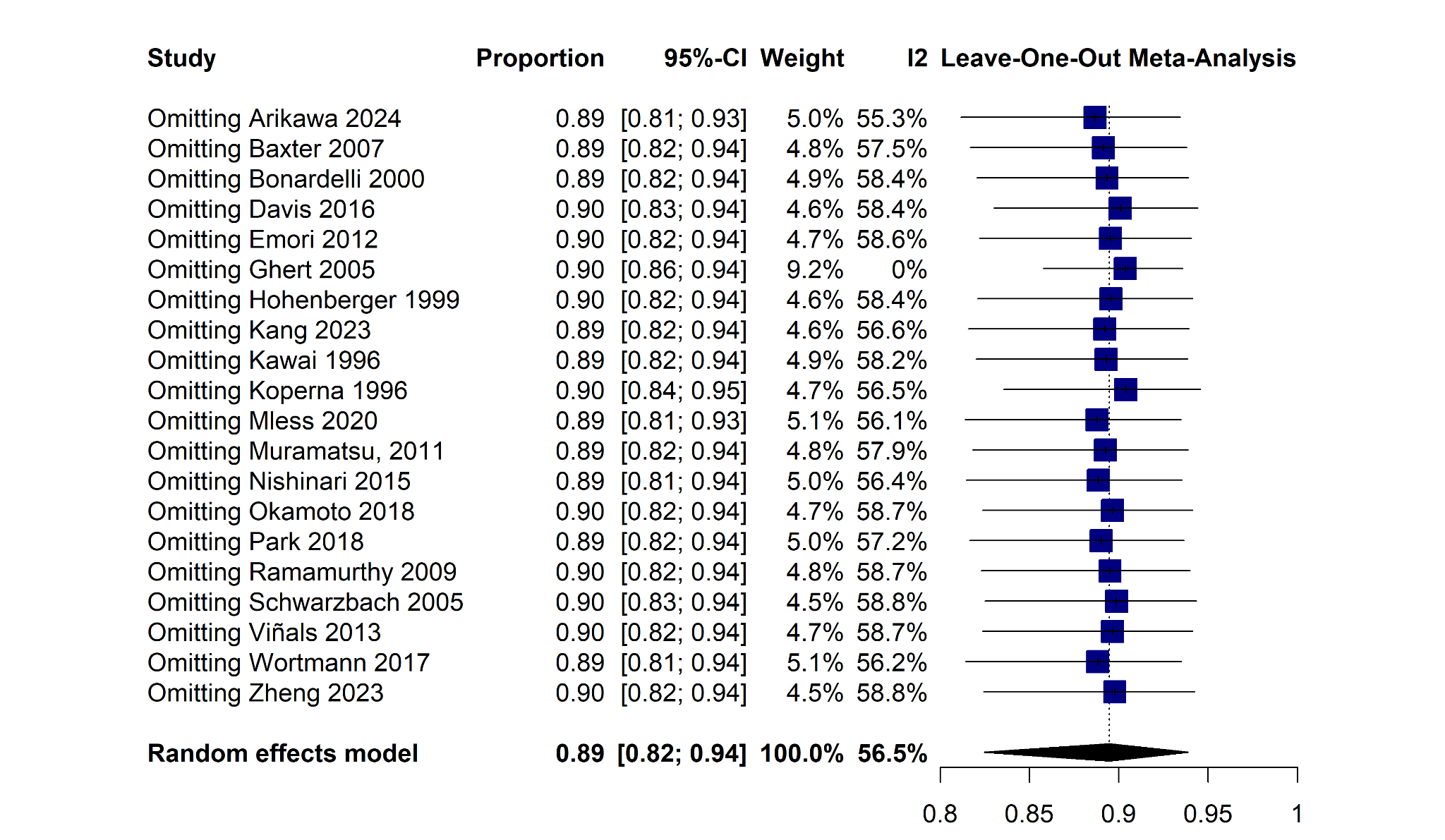


**Supplementary Figure S21.** Leave-one-out analysis of overall survival at 1 year.


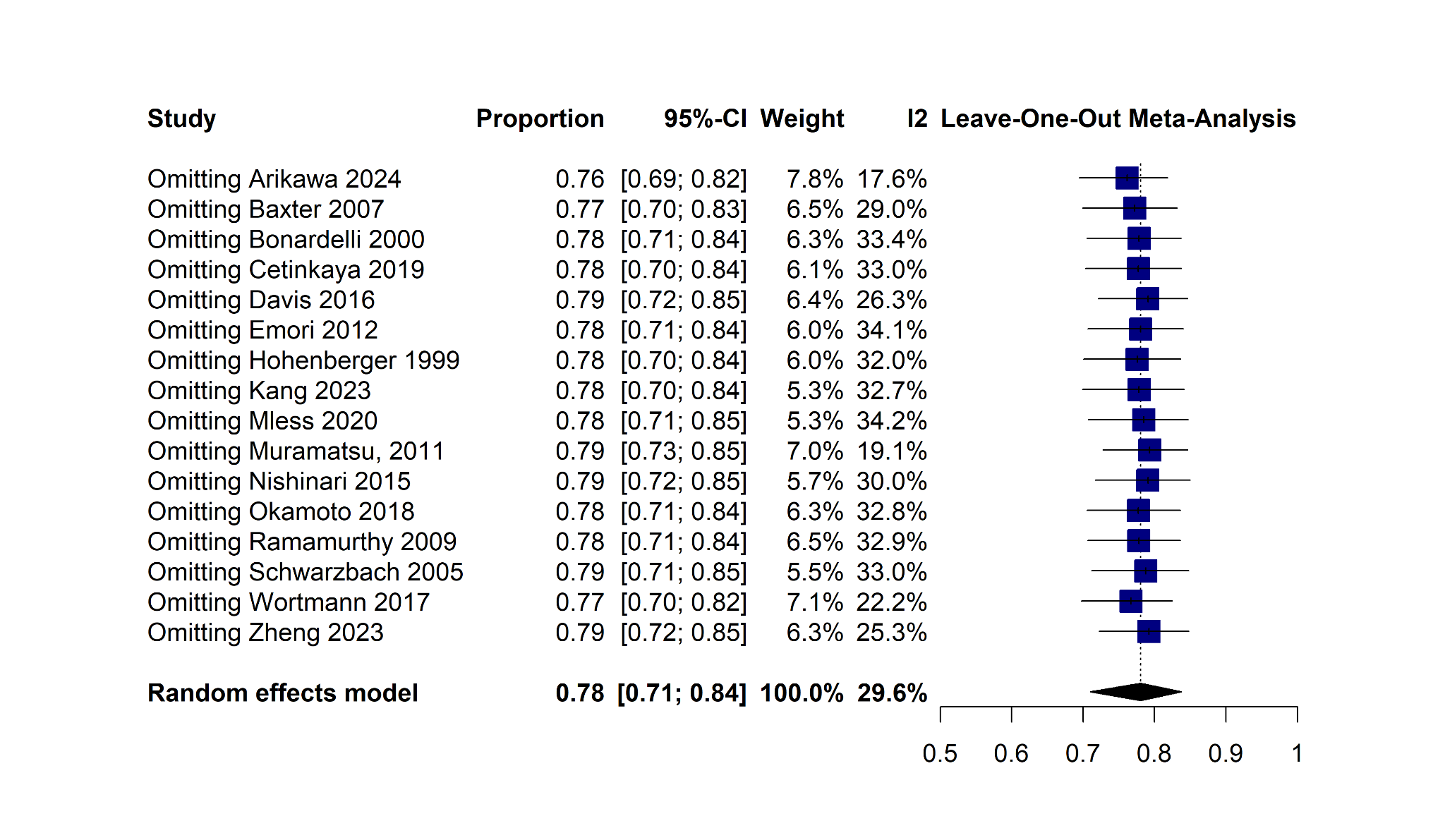


**Supplementary Figure S22.** Leave-one-out analysis of overall survival at 2 years.


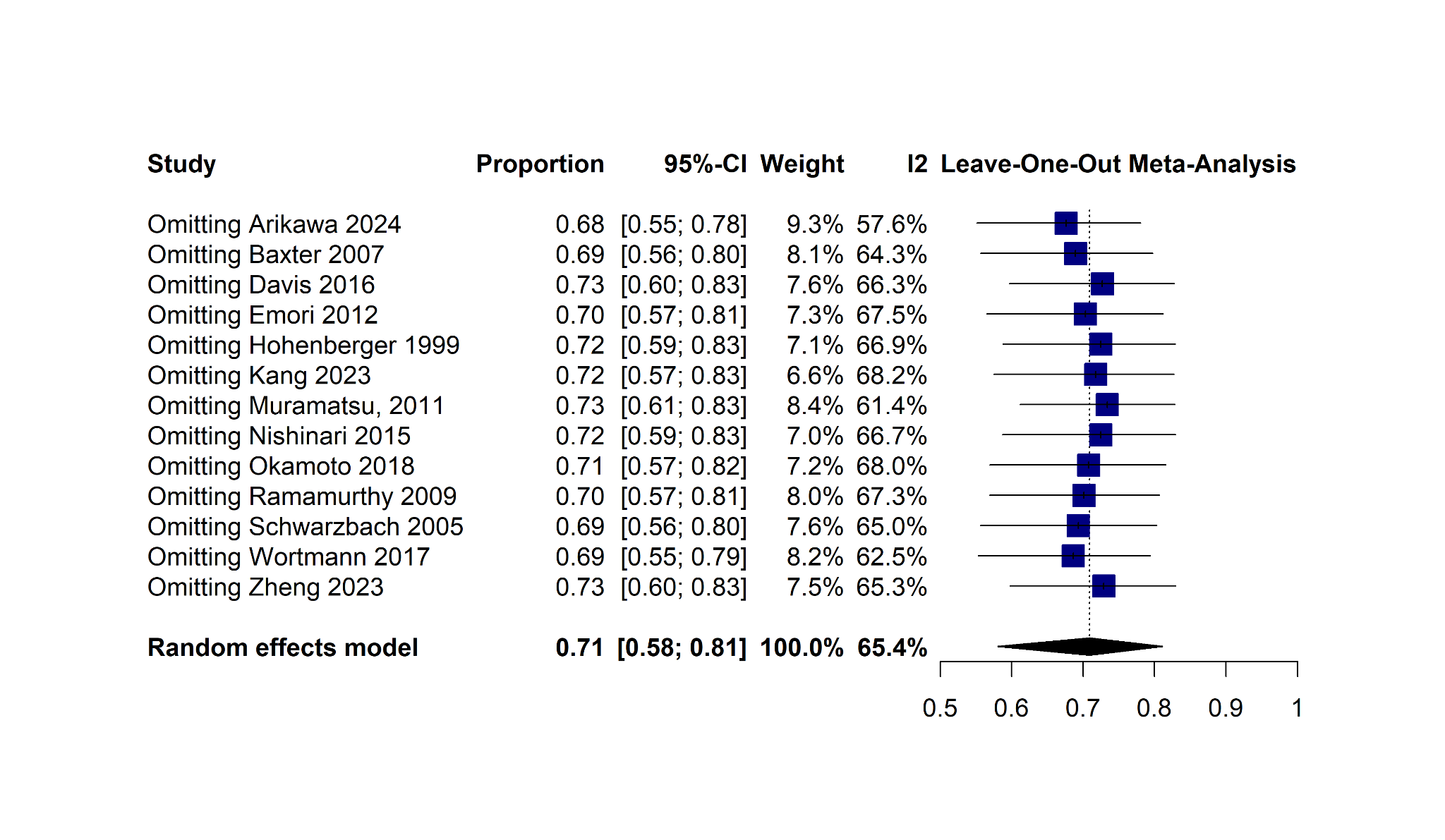


**Supplementary Figure S23.** Leave-one-out analysis of overall survival at 3 years.


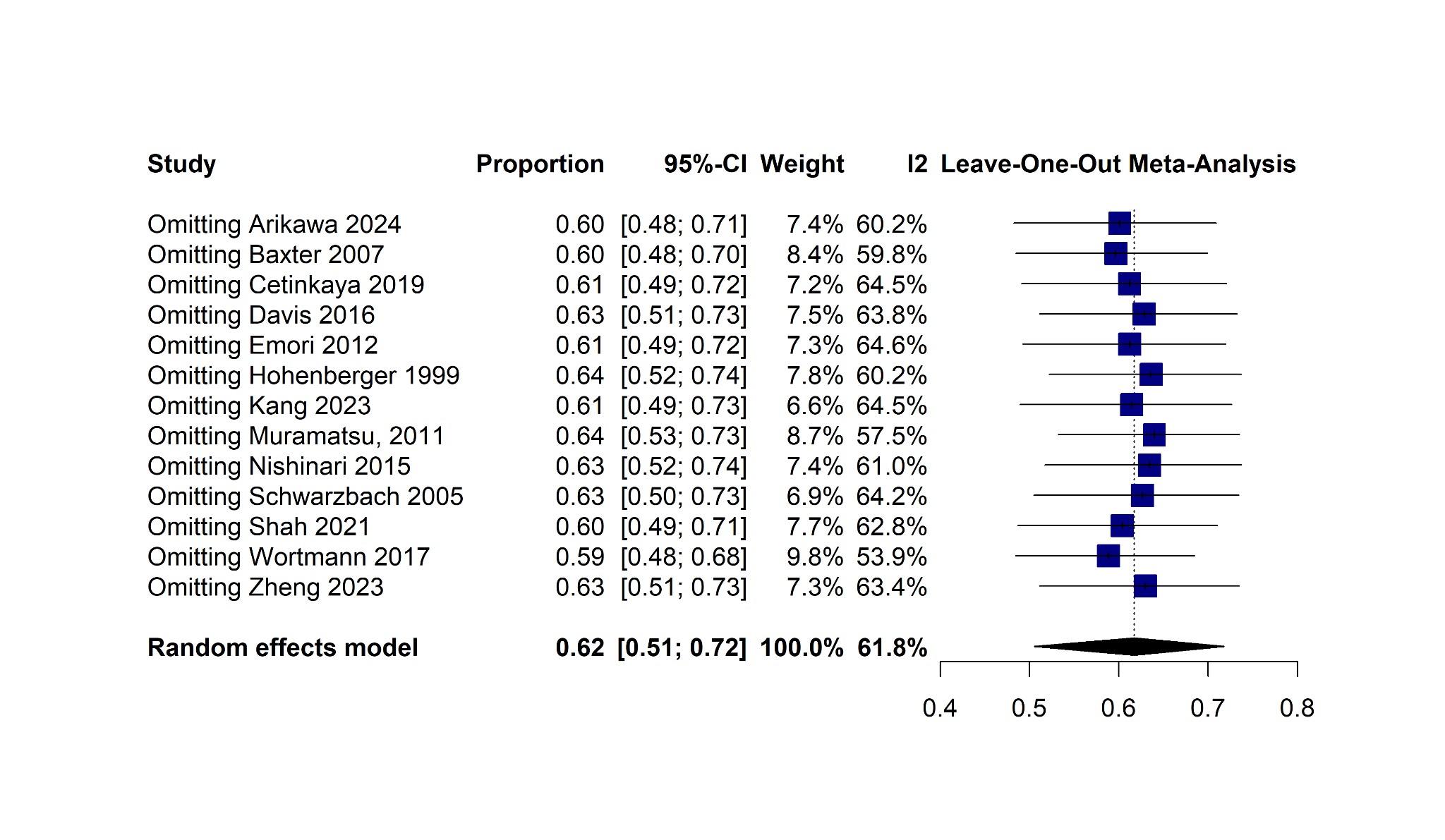


**Supplementary Figure S24.** Leave-one-out analysis of overall survival at 5 years.


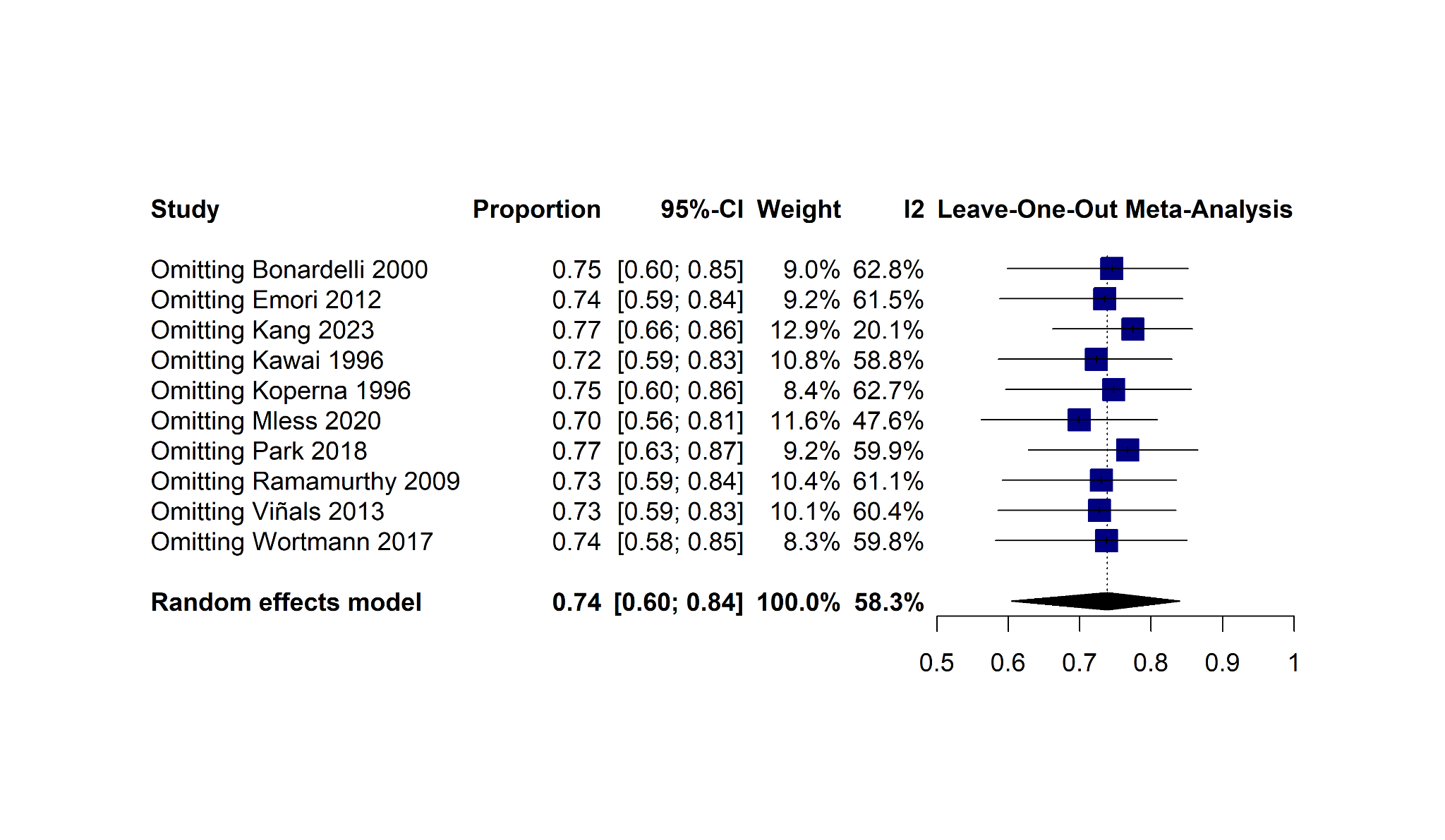


**Supplementary Figure S25.** Leave-one-out analysis of disease-free survival at 1 year.

**
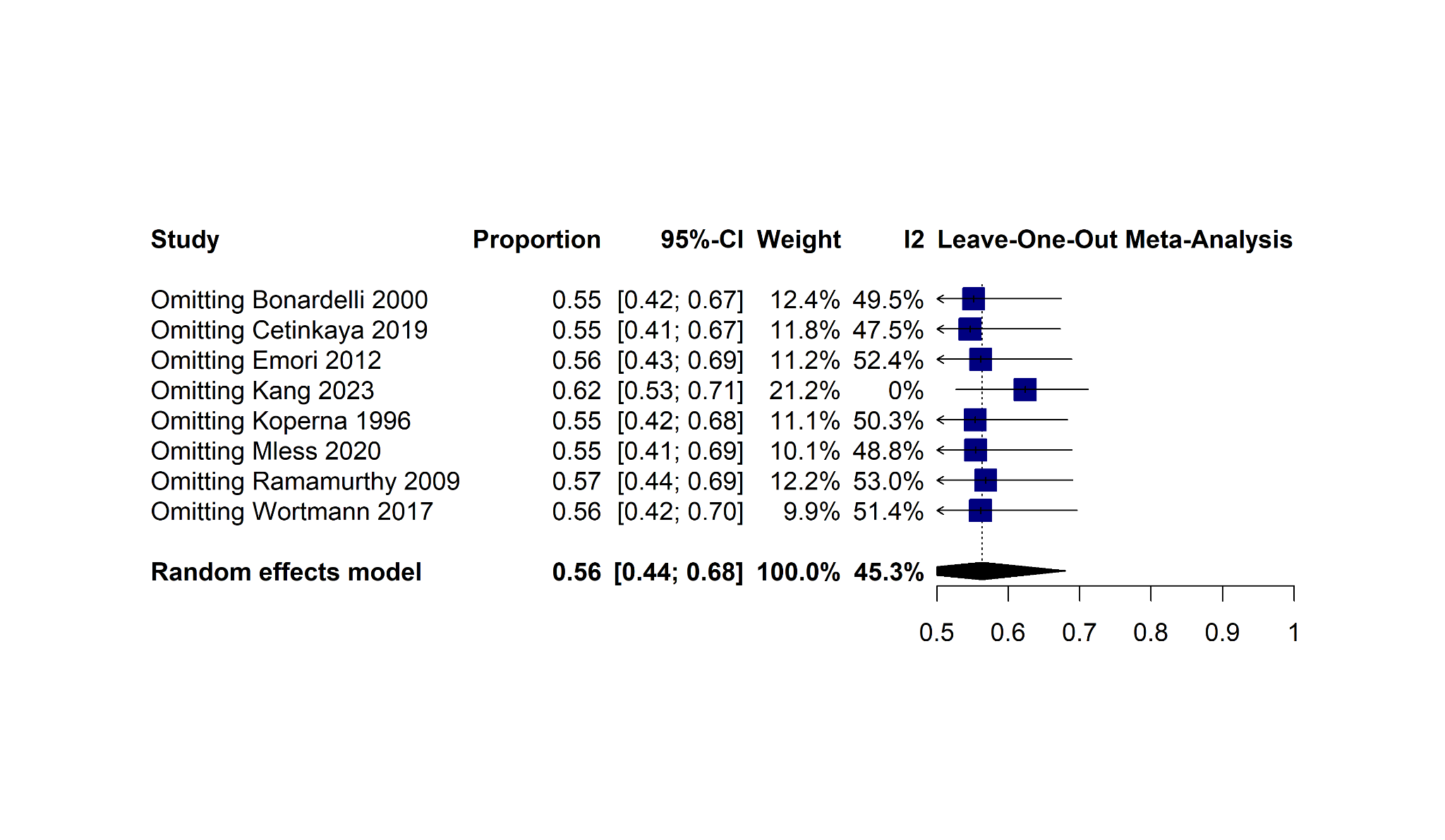
**

**Supplementary Figure S26.** Leave-one-out analysis of disease-free survival at 2 years.


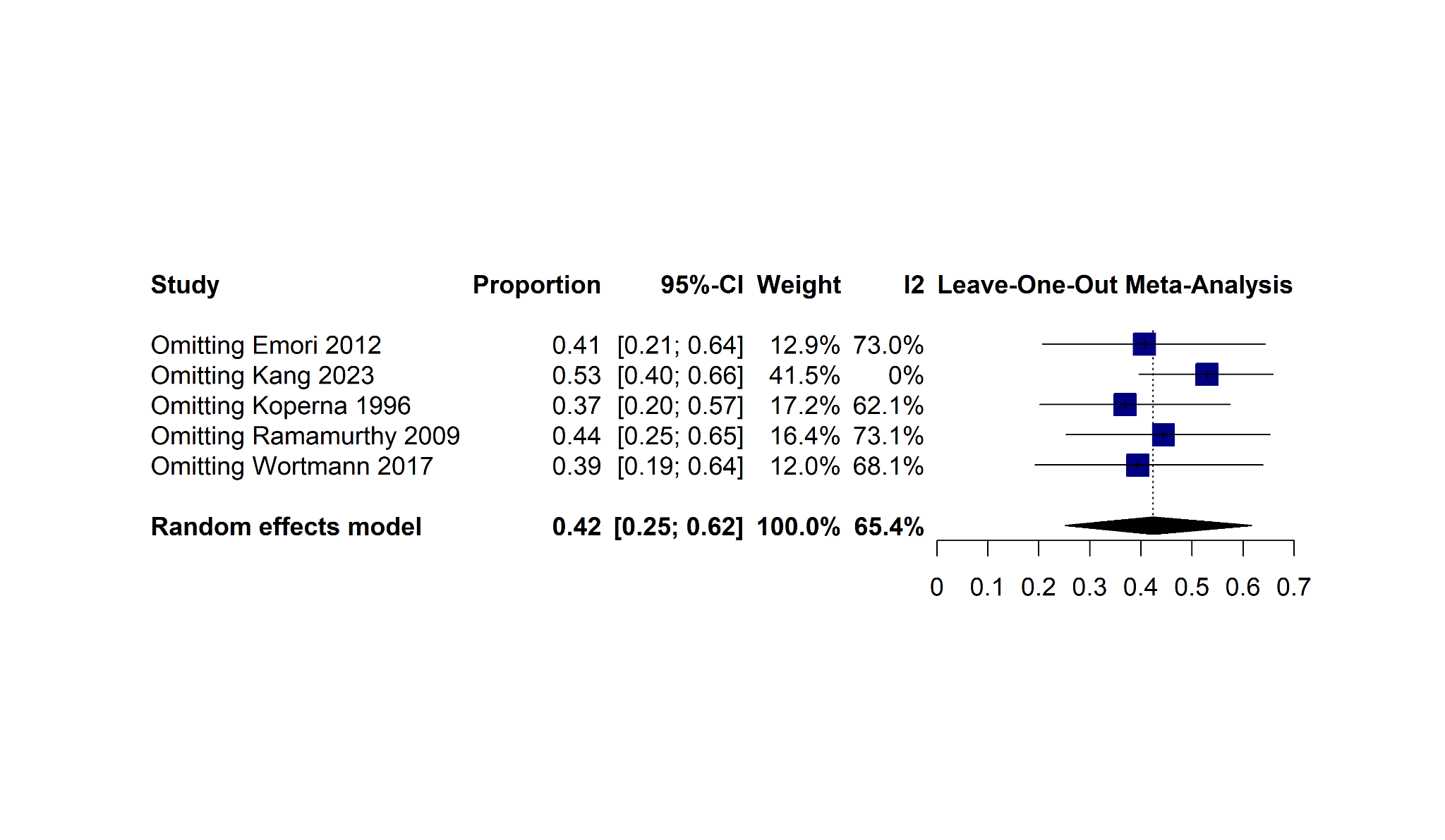


**Supplementary Figure S27.** Leave-one-out analysis of disease-free survival at 3 years.


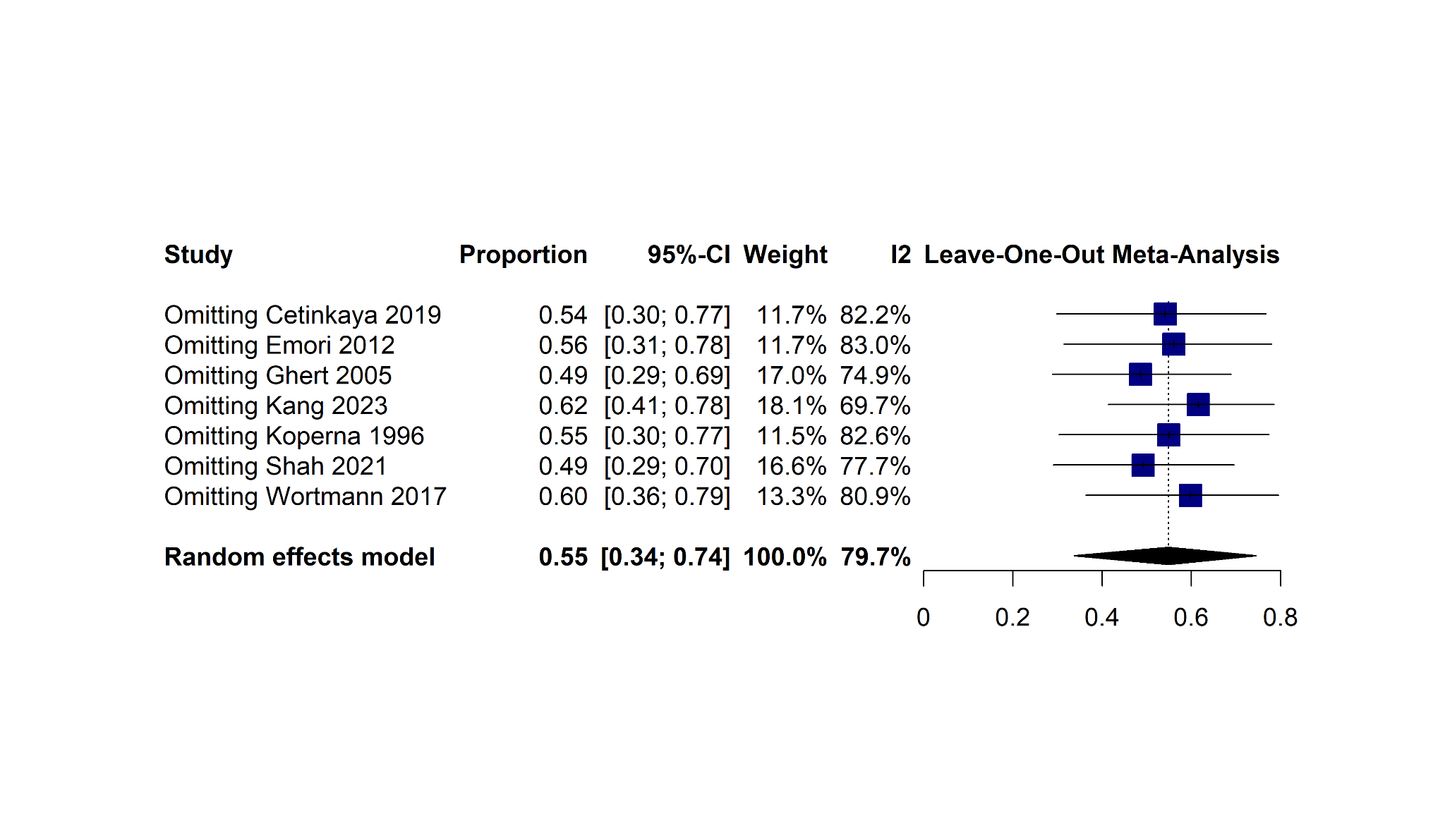


**Supplementary Figure S28.** Leave-one-out analysis of disease-free survival at 5 years.


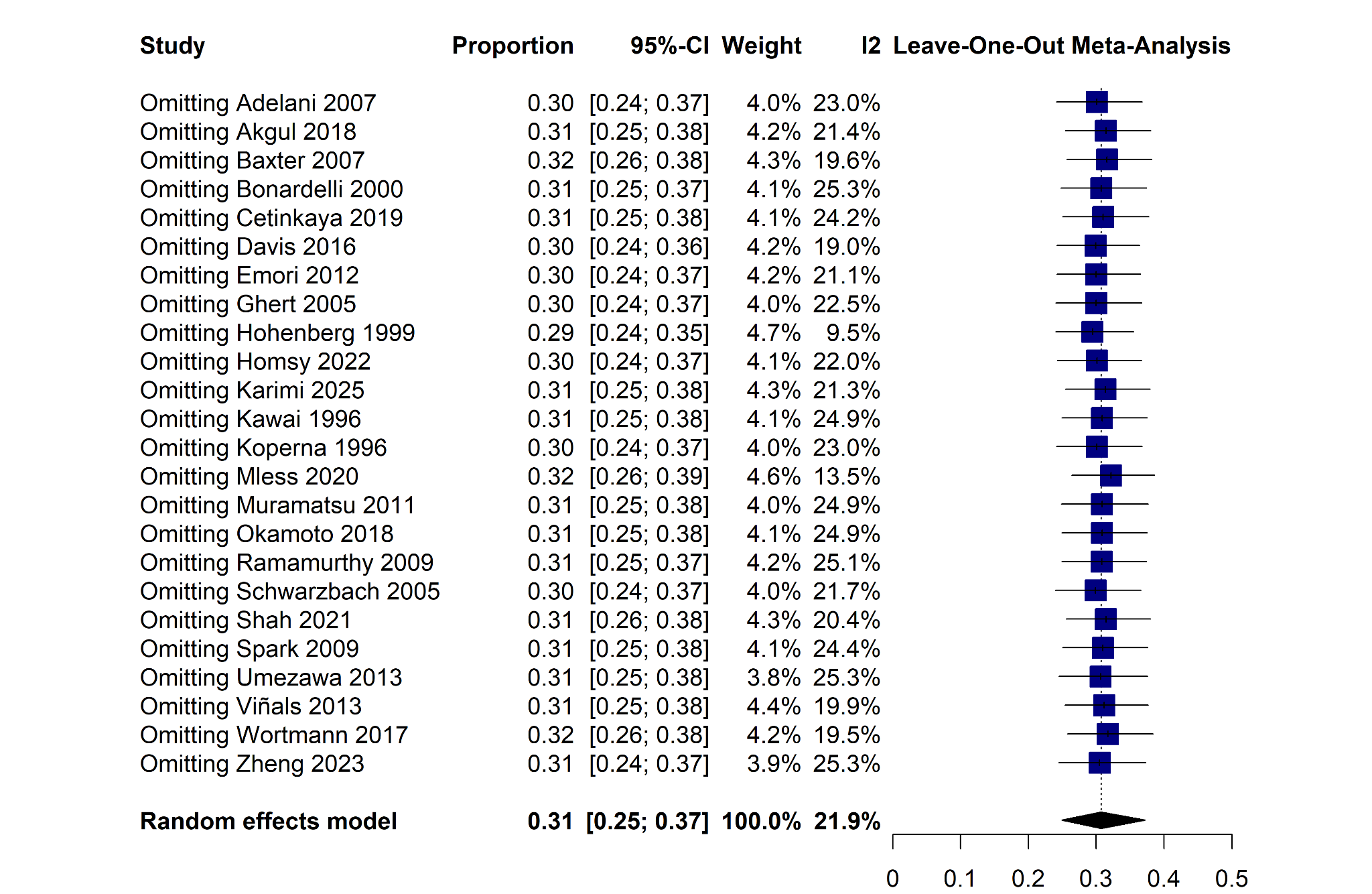


**Supplementary Figure S29.** Leave-one-out analysis of distant metastasis.


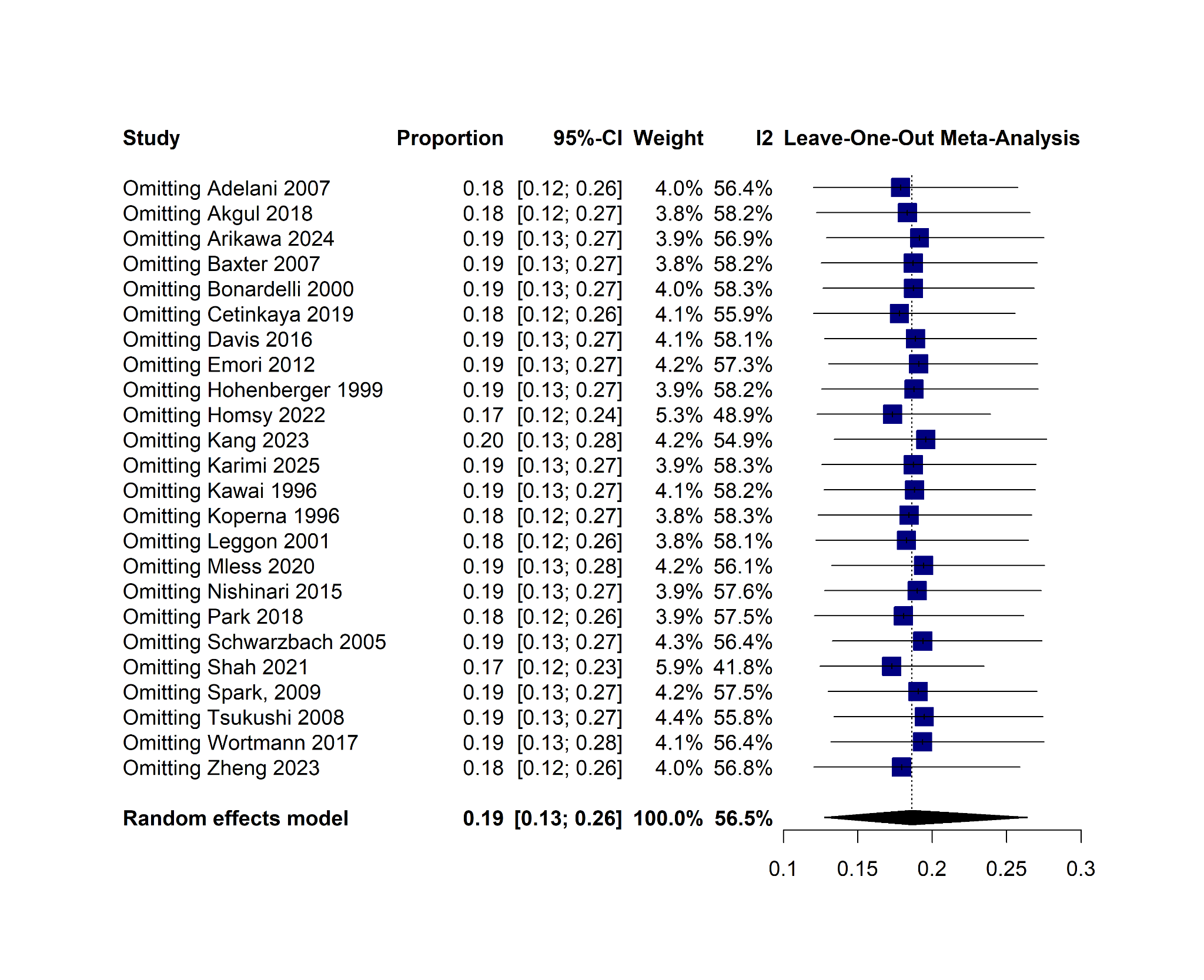


**Supplementary Figure S30.** Leave-one-out analysis of overall graft thrombosis.


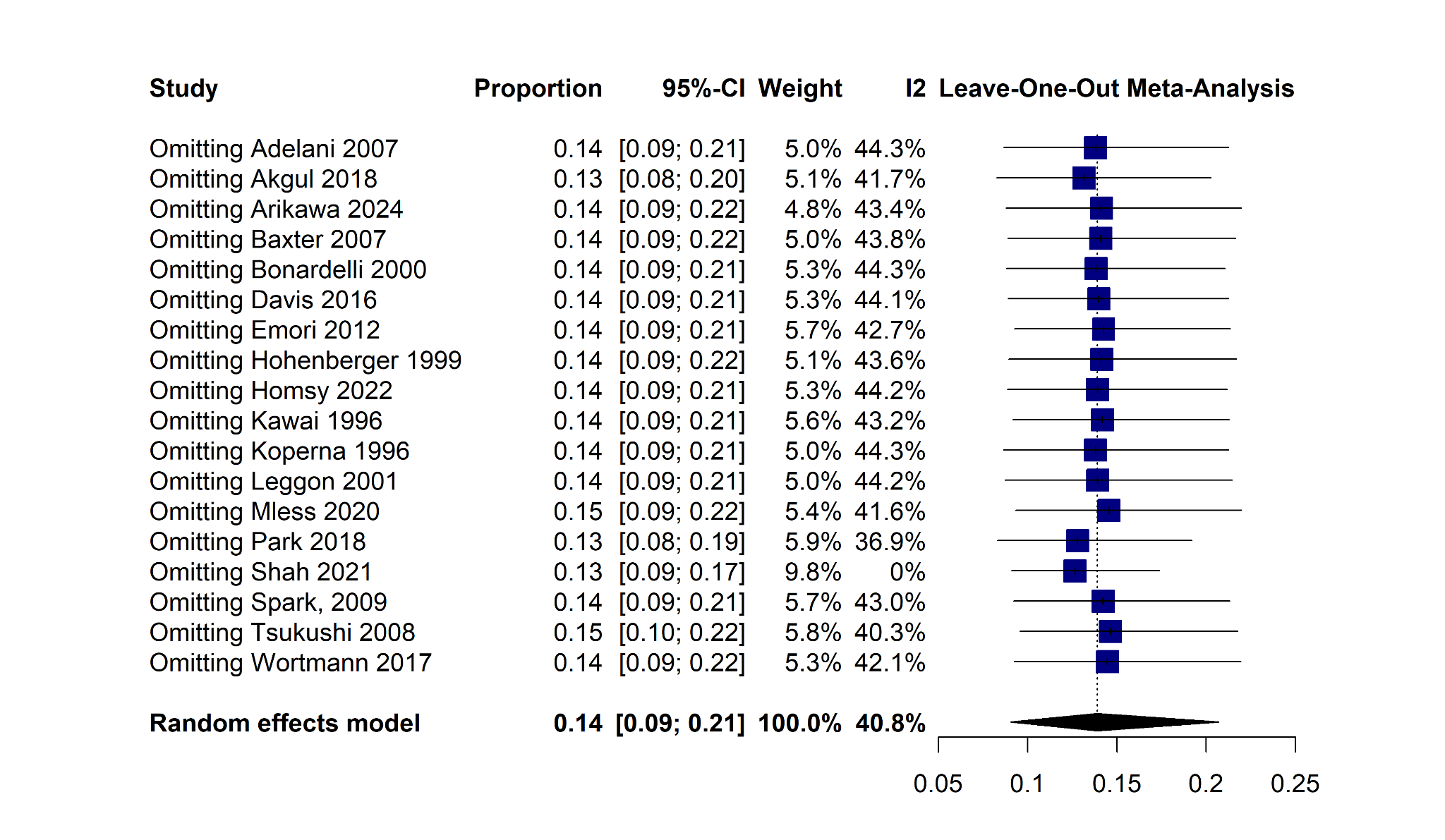


**Supplementary Figure S31.** Leave-one-out analysis of early graft thrombosis.


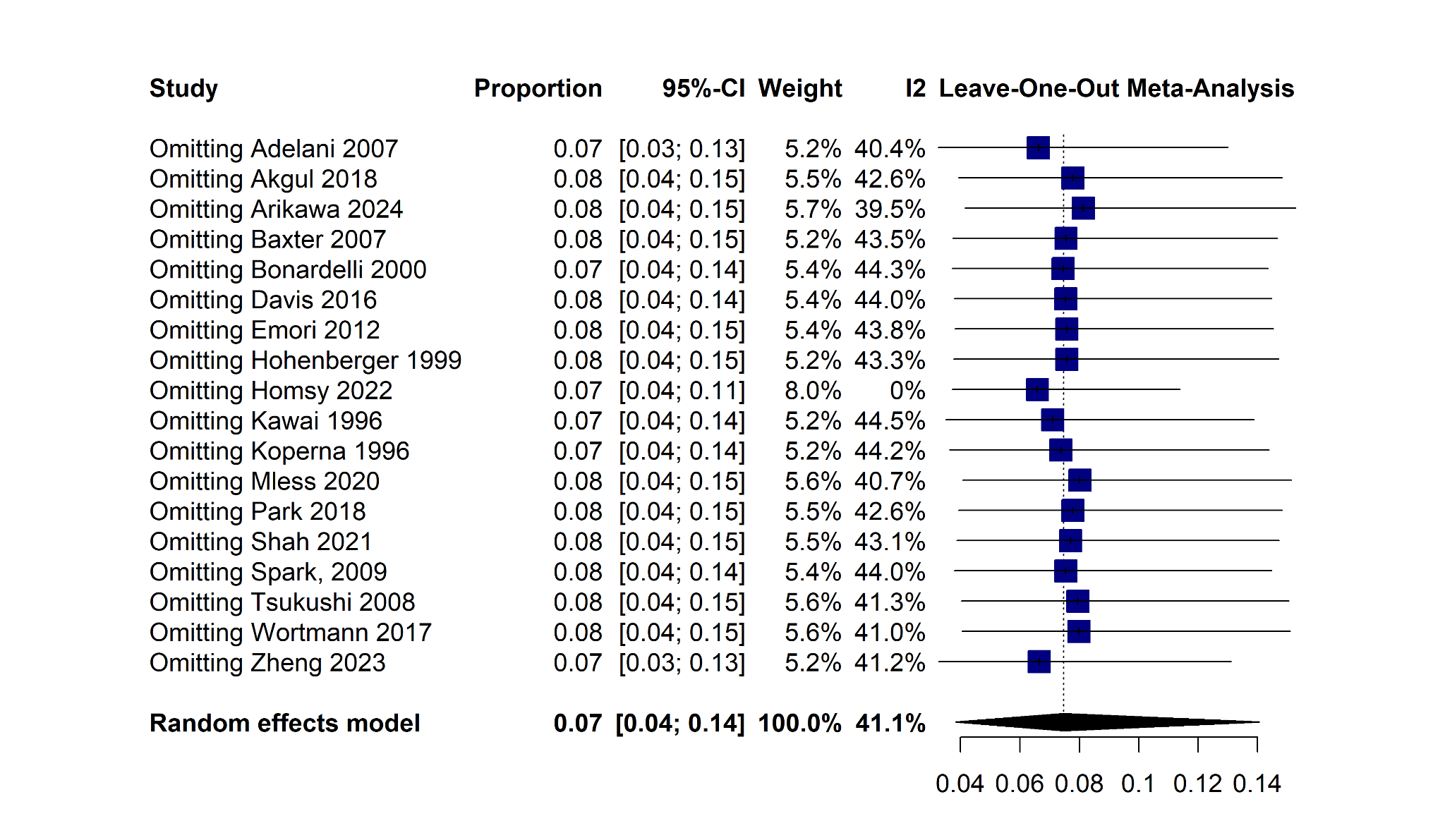


**Supplementary Figure S32.** Leave-one-out analysis of late graft thrombosis.

**
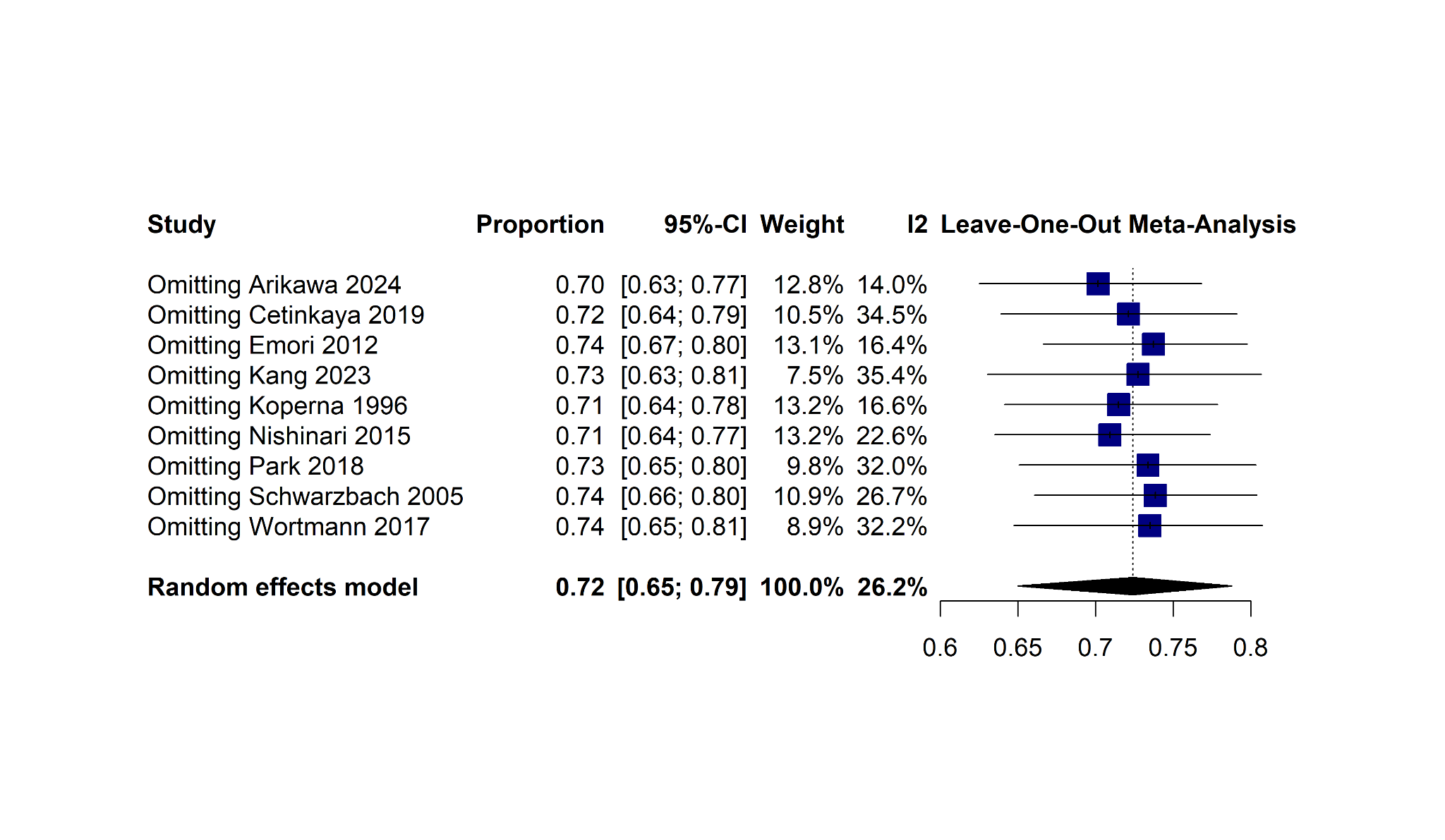
**

**Supplementary Figure S33.** Leave-one-out analysis of graft patency at 3 years.


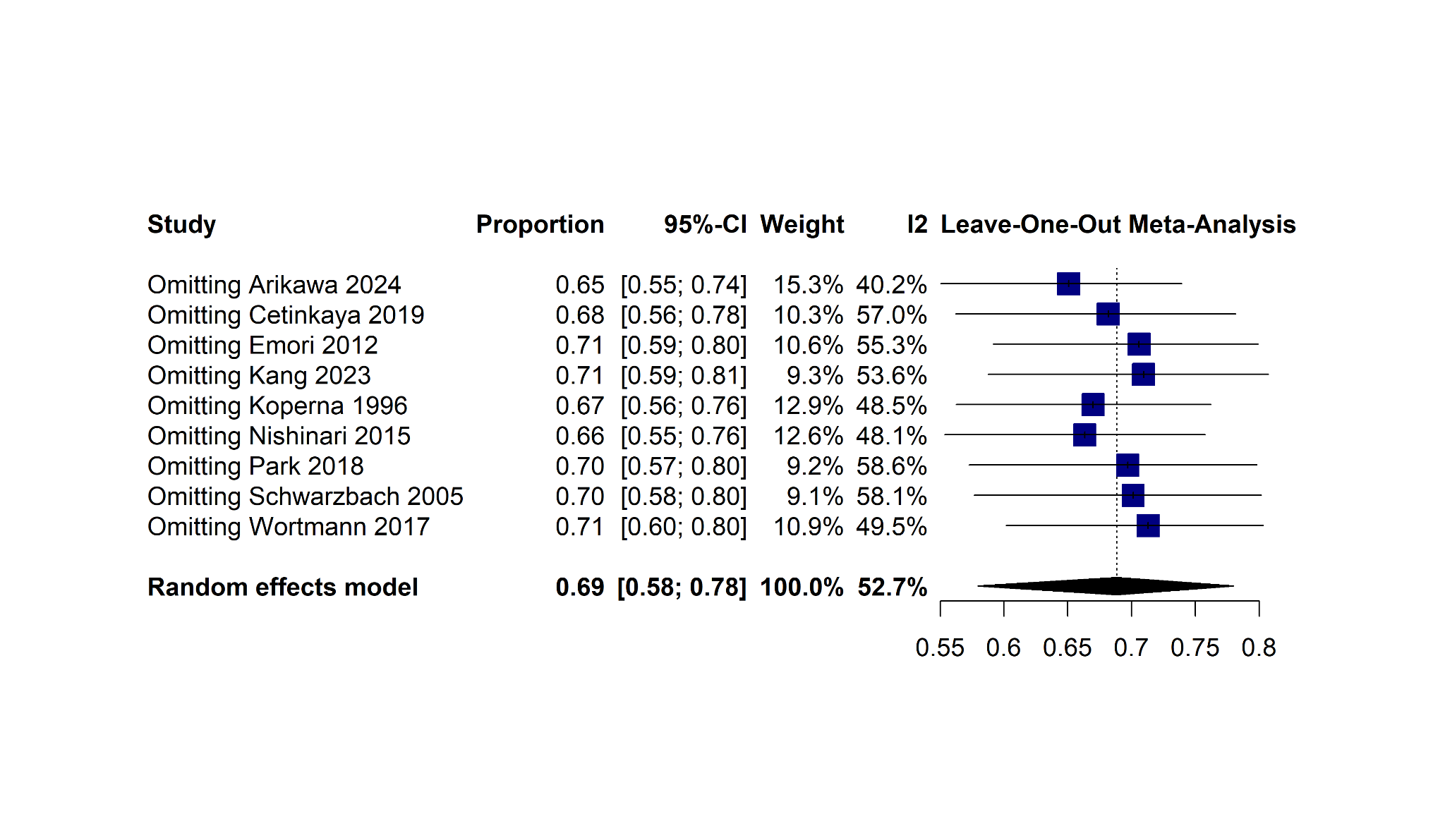


**Supplementary Figure S34.** Leave-one-out analysis of graft patency at 5 years.


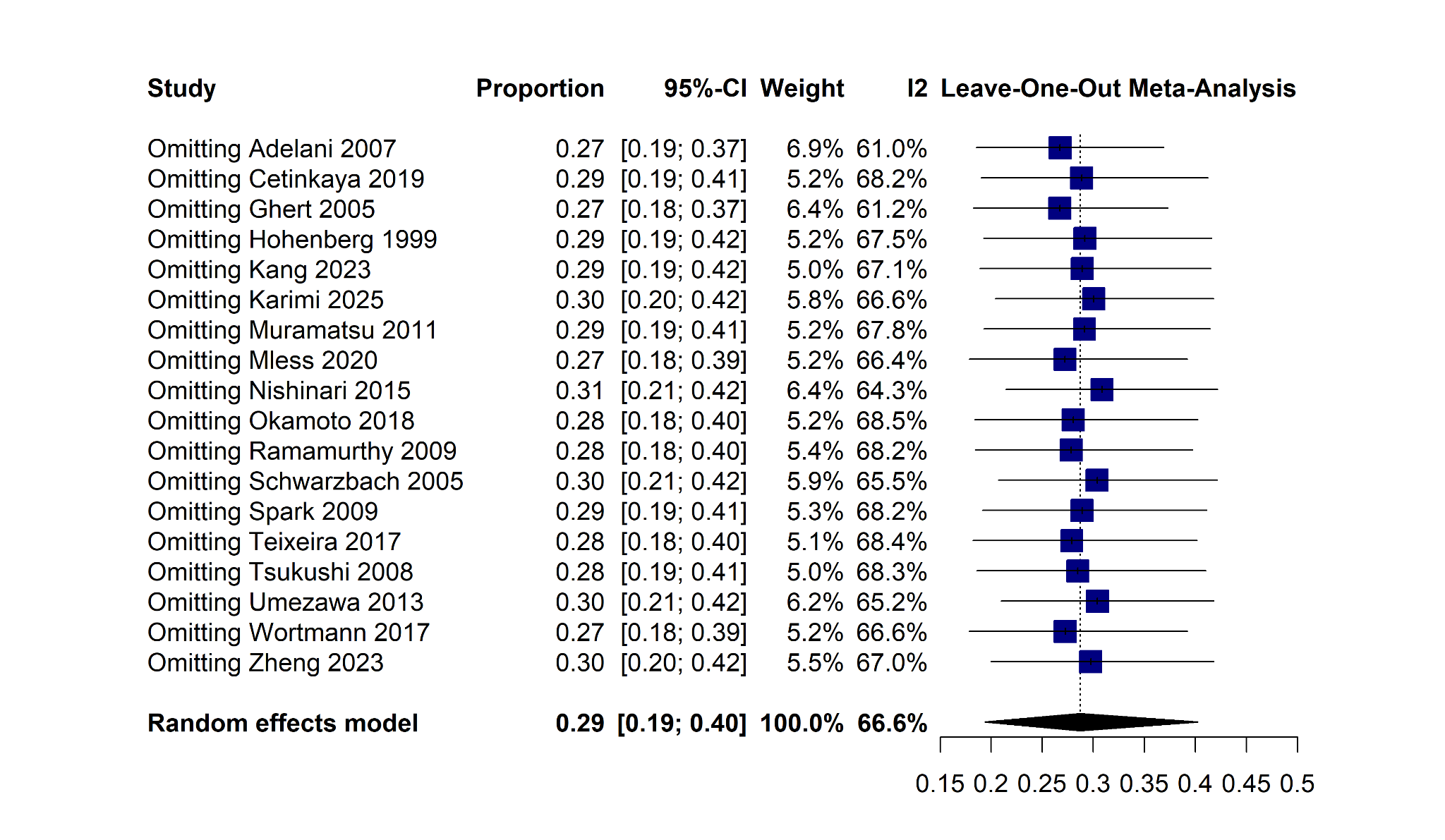


**Supplementary Figure S35.** Leave-one-out analysis of wound complication.


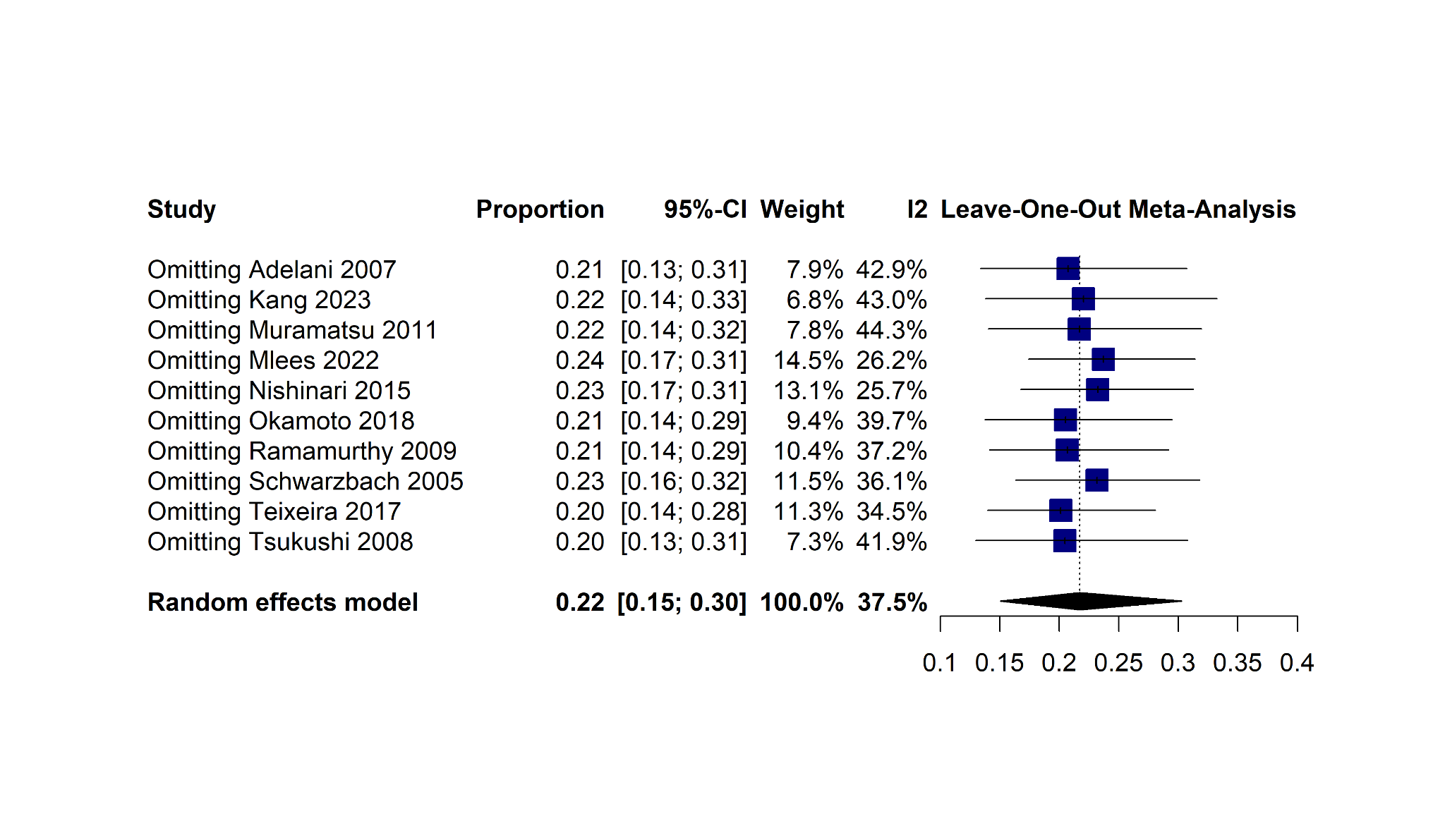


**Supplementary Figure S36.** Leave-one-out analysis of wound infection.


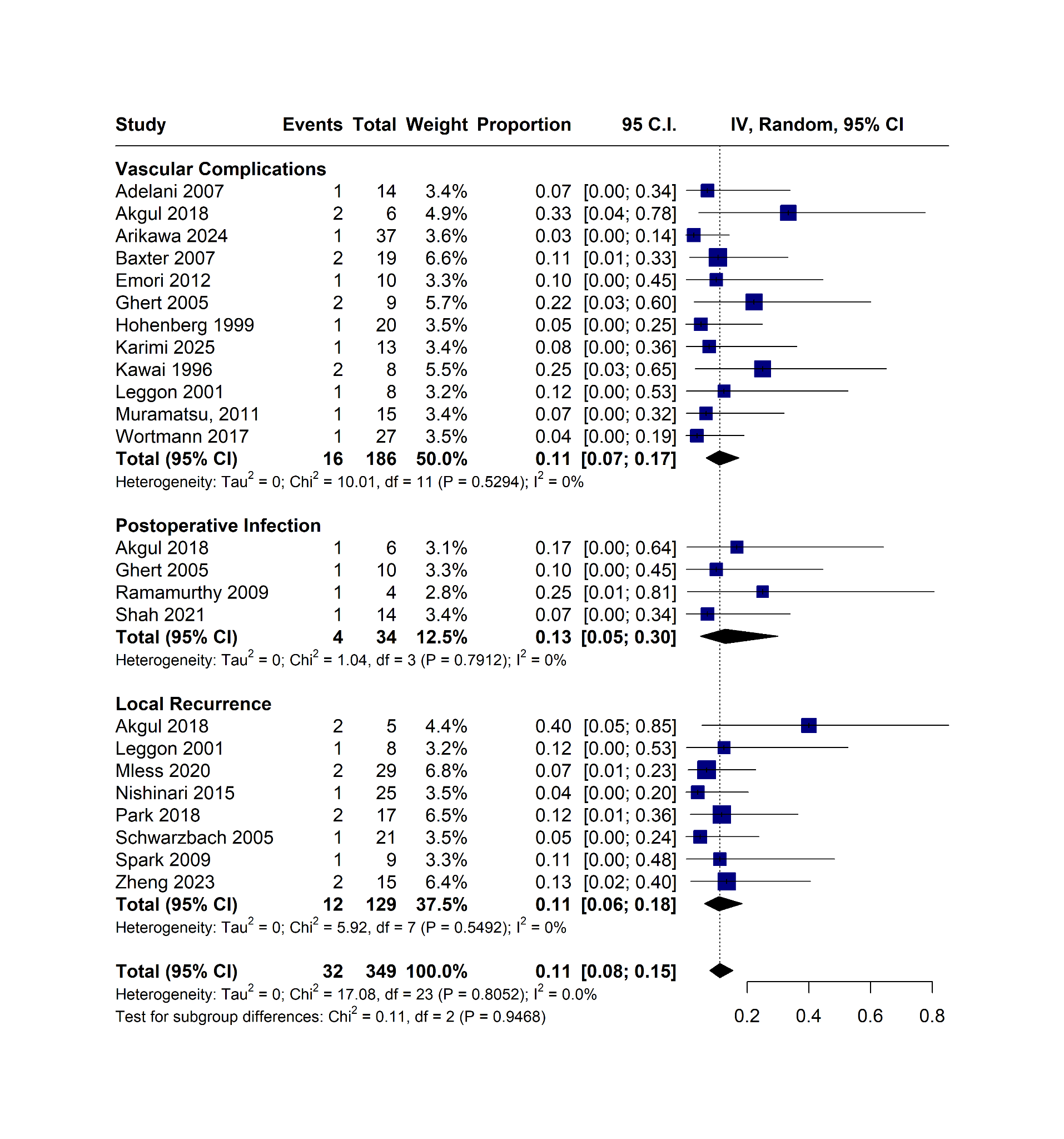


**Supplementary Figure S37.** Subgroup analysis for amputation based on cause.

**
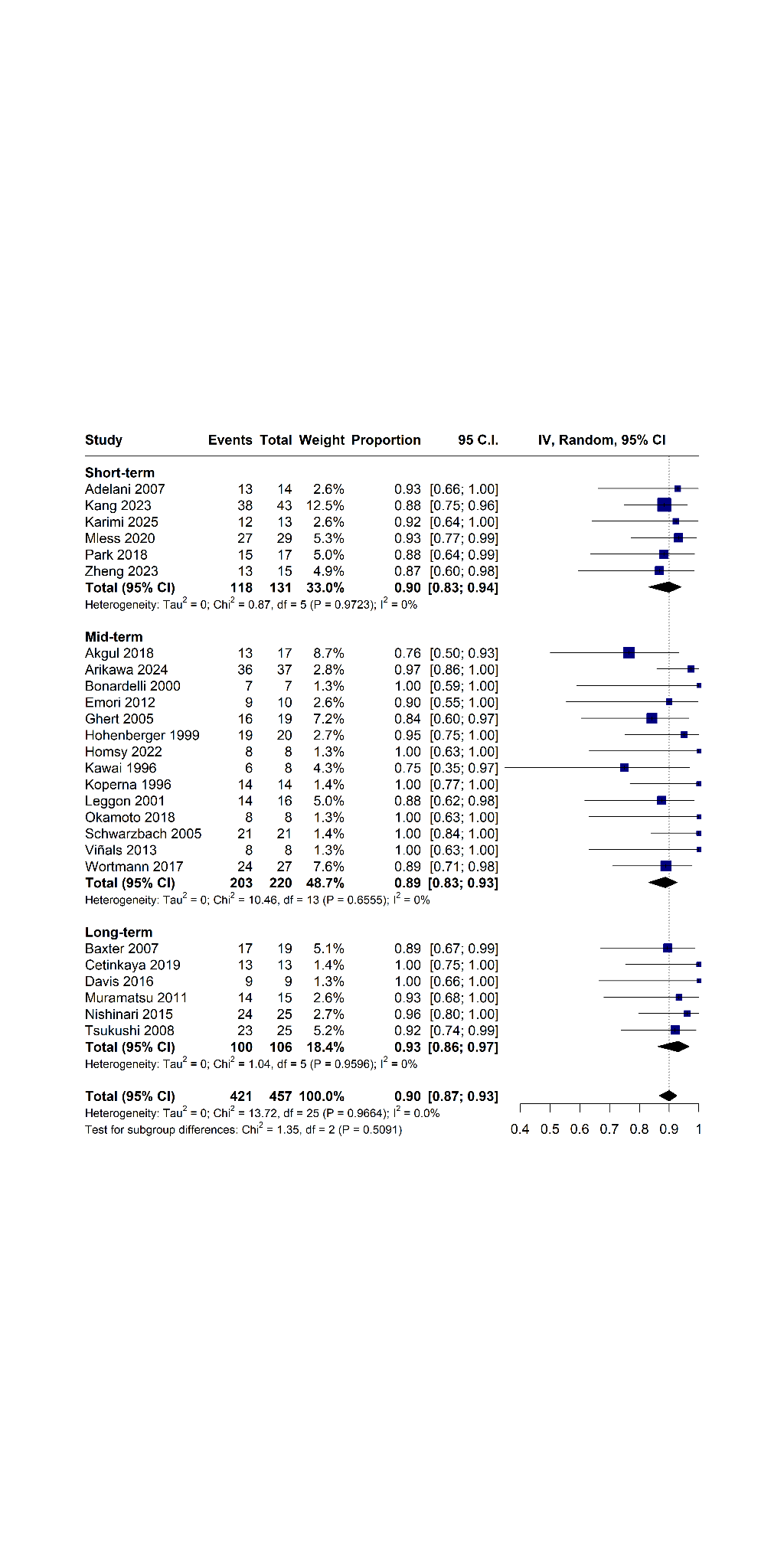
**

**Supplementary Figure S38.** Subgroup analysis for limb salvage based on follow-up time.


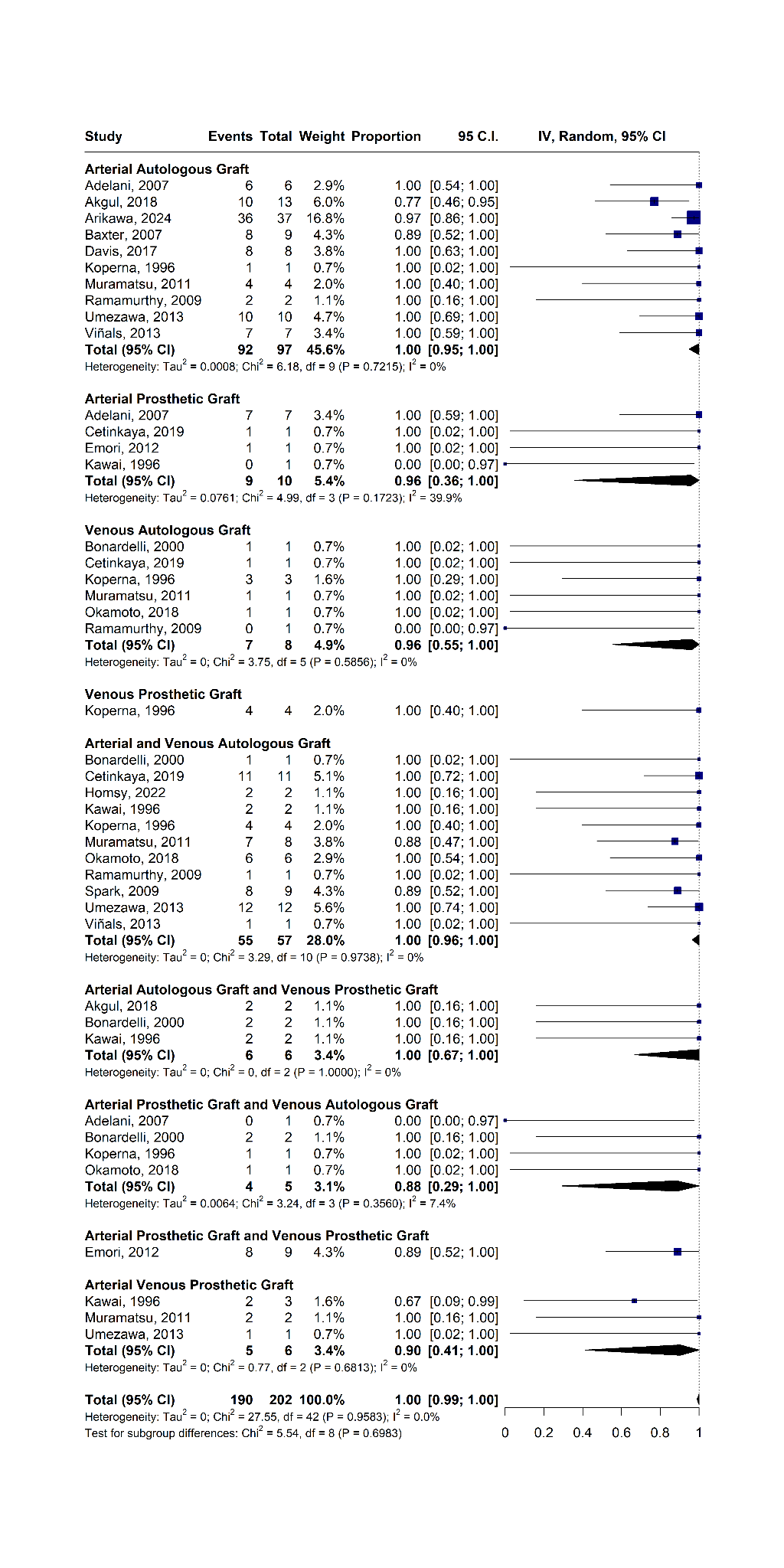


**Supplementary Figure S39.** Subgroup analysis for limb salvage based on graft type.

**
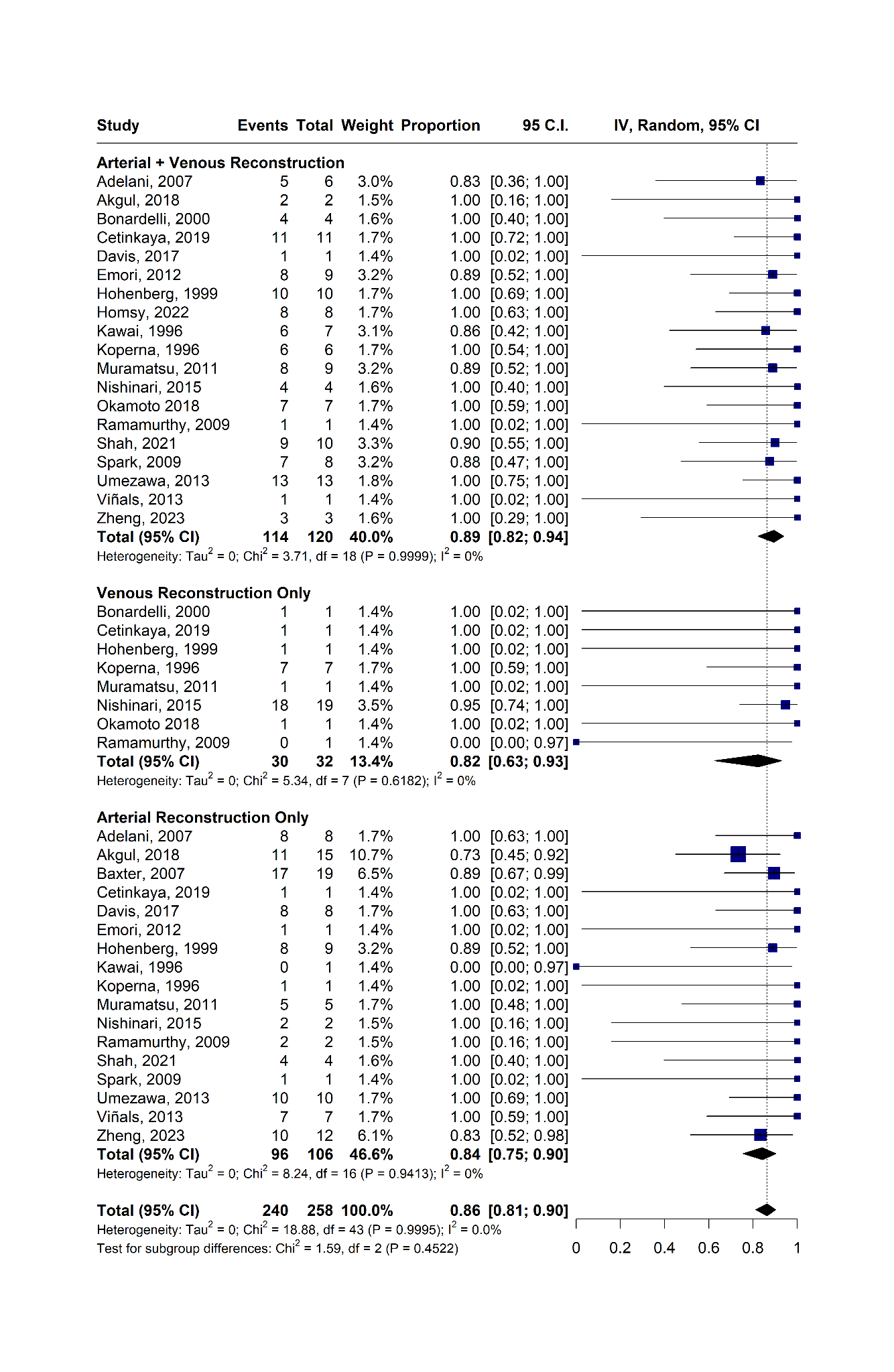
**

**Supplementary Figure S40.** Subgroup analysis for limb salvage based on reconstruction type.
